# Web-Based Personalized Machine Learning Recommendations to Enhance Shared Decision-Making in Prostate-Specific Antigen Screening: Randomized Controlled Trial

**DOI:** 10.2196/83238

**Published:** 2026-04-13

**Authors:** Yi-Ting Lin, Yen Chun Huang, Chih Kuang Liu, Hsiao-Yun Cho, Mingchih Chen

**Affiliations:** 1 Department of Urology, St. Joseph's Hospital, Yunlin, Taiwan; 2 College of Nursing, Meiho University, Pingtung, Taiwan; 3 Department of Artificial Intelligence, Tamkang University, New Taipei City, Taiwan; 4 Graduate Institute of Business Administration, College of Management, Fu Jen Catholic University, No.510, Zhongzheng Rd., Xinzhuang Dist., New Taipei City, New Taipei City, 242062, Taiwan, 886 02-2905-3109; 5 Department of Otorhinolaryngology—Head and Neck Surgery, Fu Jen Catholic University Hospital, Fu Jen Catholic University, New Taipei City, Taiwan

**Keywords:** prostate cancer, PSA screening, prostate-specific antigen, shared decision-making, decision aids, machine learning, personalized recommendations, decisional conflict, patient-centered care

## Abstract

**Background:**

Prostate‑specific antigen (PSA) screening involves complex trade‑offs between early detection and the risks of overdiagnosis. For older adults (aged ≥50 years), shared decision‑making (SDM) is often hindered by limited health literacy, sensory or cognitive impairments, and multimorbidity, which complicate risk comprehension. Traditional decision aids provide foundational knowledge but are often nonpersonalized. Machine learning (ML) may offer individualized recommendations, yet the psychological and behavioral effects of ML‑assisted SDM in geriatric populations remain poorly characterized.

**Objective:**

This study aimed to develop and evaluate a web‑based, ML‑driven decision aid integrated into an SDM workflow to provide personalized PSA screening recommendations and to assess its effects on decisional conflict (primary outcome), state anxiety, and decision satisfaction among middle‑aged and older men.

**Methods:**

The study followed a 2‑stage design. First, a model establishment group (n=507) was used to train and evaluate 6 ML algorithms based on clinical and values‑clarification data. A random forest model was selected for its superior performance (mean area under the curve 0.933, SD 0.350; 95% CI 0.902-0.963). Second, a randomized controlled trial was conducted with 367 participants (mean age 64.34, SD 10.30 years) randomly assigned 1:1 to the ML suggestion group (MLSG; n=185) or the control group (CG; n=182). Both groups received video‑based education, counseling, and values clarification; only the MLSG received an ML‑generated “second opinion” recommendation. Primary and secondary outcomes were assessed using the Decisional Conflict Scale (DCS), Spielberger State‑Trait Anxiety Inventory (STAI), and Satisfaction with Decision scale.

**Results:**

In the randomized controlled trial (n=367), the MLSG reported significantly lower decisional conflict than the CG (total DCS score: mean difference [MD] –3.77, 95% CI –5.55 to −1.99; Cohen *d*=–0.44; P<.001). The MLSG reported greater perceived support (DCS7: adjusted *P*=.03), more adequate advice (DCS9: adjusted *P*<.001), and higher decision confidence (DCS10: adjusted *P*=.03; DCS11: adjusted *P*<.001). Regarding psychological well‑being, although total anxiety scores did not differ, the MLSG reported reduced worry (STAI item 6: MD –0.98, 95% CI –1.20 to −0.76; *d*=–0.89; adjusted *P*<.001) and increased calmness (STAI item 1: MD 0.30, 95% CI 0.06-0.54; *d*=0.25; adjusted *P*=.01). Decision satisfaction was higher in the MLSG across all items (total Satisfaction with Decision score: MD –7.38, 95% CI –8.54 to −6.18; *P*<.001). Behavioral choices were strongly influenced by the ML recommendation: participants in the MLSG who received an “accept” recommendation were more likely to select “accept” (34/67, 50.7%) than those in the CG (44/182, 24.2%; *P*<.001). When the system suggested “not now,” only 17.8% (21/118) chose “accept,” which was lower than in the CG.

**Conclusions:**

Integrating personalized ML recommendations into SDM workflows provides emotional scaffolding for older men, reducing decisional distress and enhancing confidence without undermining autonomy. By addressing geriatric‑specific vulnerabilities through a facilitated digital interface, this ML‑driven approach complements traditional clinical consultations. These findings support the scalable integration of artificial intelligence–assisted decision support to foster patient‑centered care in aging populations.

## Introduction

Over the past 2 decades, shared decision-making (SDM) has become a cornerstone of patient-centered care, enabling individuals to actively participate in health care choices [1,2]. In this process, clinicians and patients exchange information, deliberate on alternatives, and align decisions with patient values [3-5]. Guidelines generally outline 5 key steps in SDM: engaging the patient, presenting options, clarifying values, making the decision, and reviewing outcomes [6]. This structured approach has been shown to not only enhance patient satisfaction but also reduce decisional conflict [7-9].

Prostate cancer remains one of the most common malignancies among men globally. However, the decision to undergo prostate-specific antigen (PSA) screening involves complex trade-offs between the benefits of early detection and the risks of overdiagnosis or overtreatment [10-12]. Among older adults, SDM is indispensable because health literacy limitations, sensory or cognitive impairments, and multimorbidity can hinder risk comprehension and value clarification, thereby increasing reliance on physician-directed choices [13-16]. In time-pressured encounters, lower educational attainment further diminishes active patient participation, shifting decisions toward clinician preferences unless patient values are explicitly elicited and supported through tailored explanations [3,15-17].

Consequently, strategies to strengthen SDM for older adults should prioritize cultivating strong physician-patient relationships, explicitly addressing psychological determinants of choice, and implementing patient-tailored decision aids. Collectively, these approaches have been shown to improve knowledge without increasing anxiety and can sustain reductions in decisional conflict over time, helping patients critically appraise the benefits, risks, and uncertainties of PSA testing [18-20].

Efforts to support SDM in PSA screening have primarily relied on traditional decision aids (DAs), such as educational brochures, interactive questionnaires, and electronic platforms or videos [3,6,21]. Although machine learning (ML) approaches have gained traction in clinical practice, their application has largely focused on risk prediction rather than personalized decision support. Notably, fully personalized electronic decision interfaces remain underdeveloped [22-24]. Furthermore, few studies have explicitly evaluated the impact of ML-assisted recommendations on psychological outcomes—such as anxiety, uncertainty, and decisional conflict—which are critical indicators of acceptability and long-term efficacy.

To address these gaps, we developed a novel ML-based decision aid designed as a complementary component within existing SDM workflows for PSA screening consultations. Distinct from traditional aids, this tool integrates individualized risk profiles with structured preference elicitation to align recommendations with both patient values and clinical outcomes observed in comparable cohorts, thereby facilitating preference-concordant choices in geriatric care.

The primary objective of this study was to evaluate whether this ML-integrated strategy reduces decisional conflict (primary outcome) among middle-aged and older men compared to standard care. Secondary objectives included assessing its impact on decision self-efficacy, state anxiety, and decision regret, as well as its potential to improve informed choice and downstream quality-of-life outcomes. These findings will inform the scalable integration of ML-based recommendations into routine PSA screening workflows, with the ultimate goal of reducing overtreatment while empowering informed patient choice.

## Methods

### Establishment of a Preference-Sensitive DA

Drawing on the frameworks proposed by Eila et al [18], Rychetnik et al [25], and Ferrante et al [26], we developed the “importance for physiological and psychological impact” (IPPI) questionnaire to systematically evaluate participants’ values and preferences regarding concerns related to PSA screening. The instrument consisted of 10 items across two dimensions: 4 assessing physiological impacts and 6 evaluating psychological impacts. The full questionnaire is available in Multimedia Appendix 1.

### Validation of Questionnaires

This 10-item questionnaire underwent expert validity assessment by 10 urologists, each with more than 7 years of clinical experience. Reliability analysis, based on data from the first 300 patients, demonstrated strong internal consistency, with Cronbach α values of 0.838 for the physiological impact dimension and 0.900 for the psychological impact dimension.

Factor analysis of the IPPI was conducted using the maximum likelihood method, followed by Varimax rotation. Maximum likelihood estimation was selected for its superior parameter estimation and statistical testing capabilities [27]. Varimax rotation was used to maximize the variance of squared factor loadings, creating a simpler and more interpretable factor structure that approaches the Thurstone principle of simple structure [28,29]. This orthogonal rotation preserves factor independence, providing greater statistical stability and replicability compared to oblique methods [29]. The analysis extracted two factors: personal values concerning physiological impacts and personal values concerning psychological impacts.

In addition, the Chinese versions of the Decisional Conflict Scale (DCS) and the International Prostate Symptom Score (IPSS) were validated and used in this study (Multimedia Appendix 2). The short form of the Spielberger State-Trait Anxiety Inventory (STAI) and the Decision Satisfaction Questionnaire were translated into Chinese by 2 independent bilingual experts. Comprehensive details of all research instruments are provided in MultimediaAppendices 3  4.

### Participants Recruitment

This study used a closed, face-to-face recruitment strategy at Taiwan St. Joseph Hospital. We recruited men who were eligible if they (1) were aged 50 years or above, (2) presented for routine care between October 2017 and April 2018, and (3) were able to provide informed consent and communicate in Mandarin Chinese. This age threshold aligns with US Preventive Services Task Force and American Urological Association guidelines recommending PSA screening discussions starting at age 50, with SDM particularly emphasized for men aged 55‐69 years [30,31]. Men were excluded if they had (1) any documented psychiatric disorder that might impair decision-making capacity, (2) prior PSA testing (to ensure treatment-naïve status), or (3) a history of prostate surgery or prostate cancer. Participant identity and eligibility were verified against electronic medical records by research staff to prevent multiple entries. Notably, computer and internet literacy were not required; although the intervention used a web-based interface, all assessments and digital components were administered face-to-face by trained research staff. This supervised approach ensured accessibility for older adults regardless of technological proficiency and guaranteed that the study team had direct contact with all participants.

### Study Group Design Framework

A total of 520 participants were initially recruited for model development. After excluding 13 individuals due to low-quality responses—defined as ten consecutive identical scores or rhythmic scoring patterns—the remaining 507 participants formed the model establishment group (MEG) for comprehensive training and validation. To ensure progressive optimization, the ML model was iteratively retrained after every batch of 20 new participants. This development phase continued until the model achieved a predefined performance criterion (area under the curve [AUC] ≥0.80), indicating sufficient predictive accuracy for potential clinical application.

Subsequently, we conducted a parallel-group randomized controlled trial to evaluate the impact of ML-based decision aid recommendations. An additional 380 participants were recruited and randomly assigned with a 1:1 allocation ratio to either the ML suggestion group (MLSG, n=190) or the control group (CG, n=190). The randomization sequence was computer-generated and integrated directly into the web-based study platform to ensure allocation concealment. Group assignment was revealed to the research staff only after each participant’s baseline data had been fully entered and locked, thereby preventing any foreknowledge of the allocation sequence. The MLSG received standard SDM support supplemented by ML-assisted recommendations, while the CG received standard SDM alone. After excluding participants with low-quality responses, the final analysis included 185 participants in the MLSG (n=5 excluded) and 182 in the CG (n=8 excluded). Prior to study commencement, all participating medical staff completed a standardized 10-minute training session to ensure proficiency in navigating the DA. The study was conducted in accordance with the original protocol, and there were no changes to the methods (including eligibility criteria and outcomes) after trial commencement.

### Ethical Considerations

This study was conducted in accordance with the Declaration of Helsinki and relevant ethical guidelines for research involving human subjects. The study protocol was reviewed and approved by the Institutional Review Board of Fu Jen Catholic University (approval C105142), and written informed consent was obtained from all participants before enrollment. The trial was registered in the Chinese Clinical Trial Registry (ChiCTR 2000034126). Only the patient medical record number was retained to protect privacy, and all study data were accessible exclusively to authorized research personnel; nonresearch staff were not permitted to access the dataset. To promote transparency and fairness, participants received a cash payment of NT $300 for their participation, which is equivalent to approximately US $9.38 based on an exchange rate of US $1=NT $31.97.

### Intervention Specification

#### Intervention Flow and Core Components

**Figure 1.**
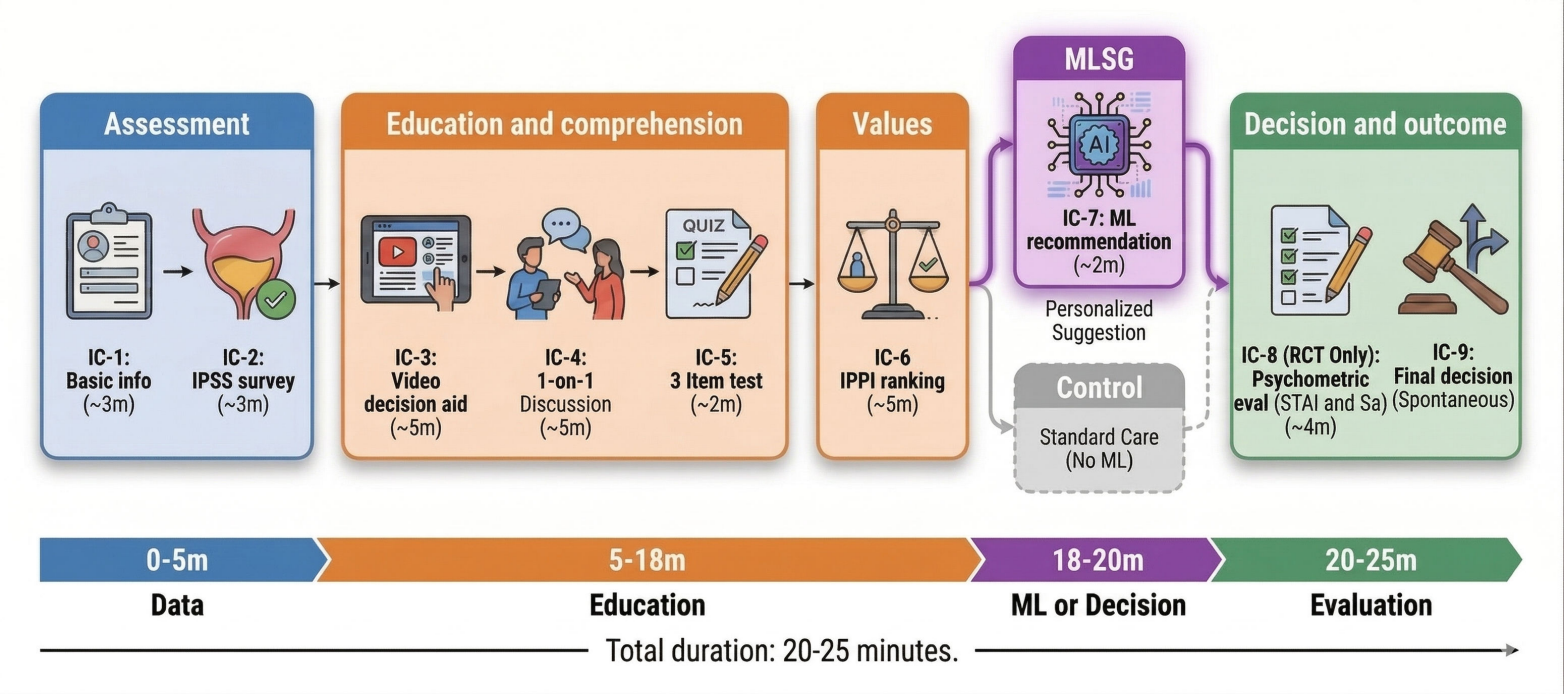
Prostate-specific antigen screening decision support intervention procedure. IC: informed consent; IPPI: importance for physiological and psychological impact; IPSS: International Prostate Symptom Score; ML: machine learning; MLSG: machine learning suggestion group; RCT: randomized controlled trial; Sa: Satisfaction with Decision scale; STAI: Spielberger State-Trait Anxiety Inventory.

The intervention comprised 9 standardized components delivered in approximately 20‐25 minutes (Figure 1). Participants in the MEG completed components IC-1 to IC-6, whereas participants in the randomized controlled trial (RCT) completed the full sequence (IC-1 to IC-9). Initially, all participants provided baseline demographic and clinical data (IC-1; ~3 min), followed by a urinary symptom assessment using the IPSS (IC-2; ~2 min). The video-based decision aid (IC-3; ~5 min) then provided a structured introduction to PSA screening, sequentially covering (1) prostate physiology, (2) potential morbidity of prostate disease, and (3) benefits of early detection versus risks of overdiagnosis.

At IC-4 (~5 min), trained medical personnel conducted individual counseling to ensure comprehension. Using neutral, nondirective language and the teach-back method, staff confirmed understanding and corrected misconceptions without soliciting patient preferences. Participants were explicitly reminded that the final screening decision remained entirely their prerogative. Subsequently, at IC-5, participants completed a brief 3-item knowledge assessment (true/false/unsure) covering screening accuracy, disease progression, and biopsy limitations (items detailed in Multimedia Appendix 5). At IC-6 (~3 min), participants engaged in the IPPI values-clarification exercise, rating the personal importance of screening attributes and ranking them by priority.

#### Facilitated ML Recommendation (IC-7; MLSG Only)

**Figure 2.**
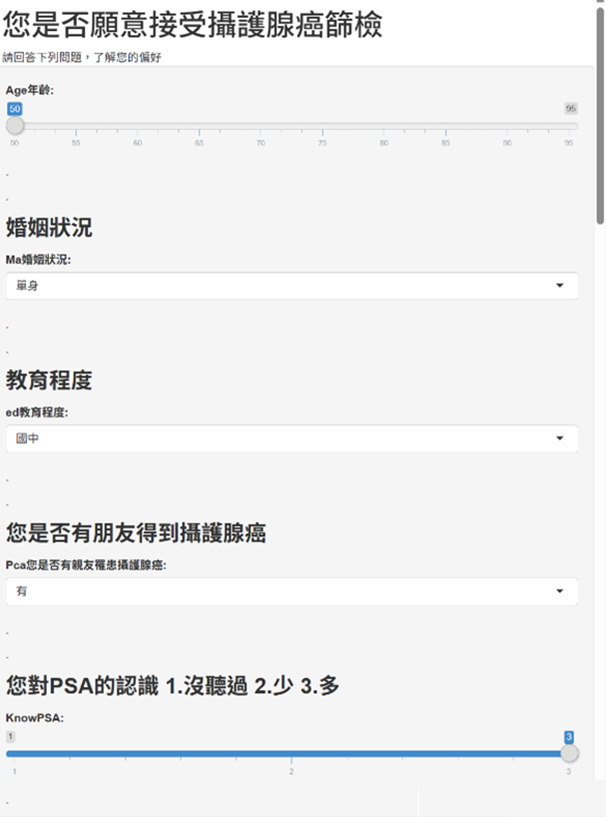
Screenshot of the web-based decision aid interface (original traditional Chinese version). Integrated participant interface for data entry. Full English translations are provided in Multimedia Appendix 6.

**Figure 3.**
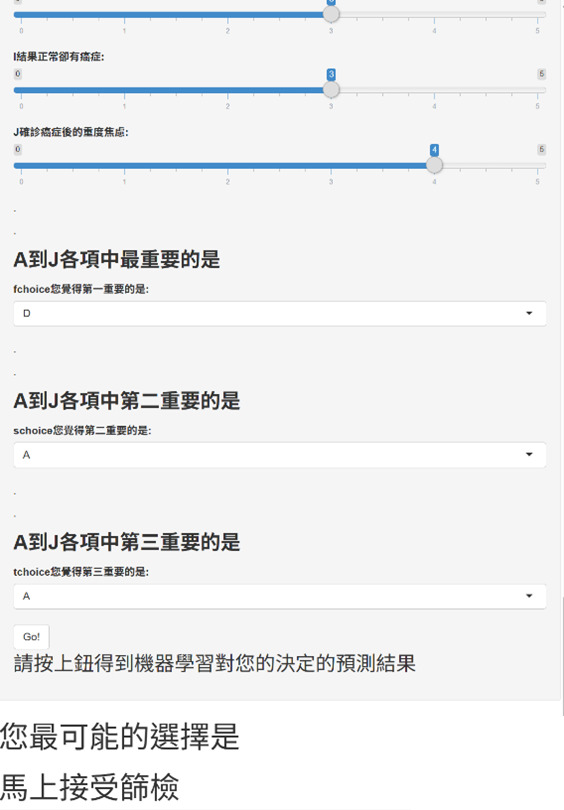
Screenshot of the web-based decision aid interface (original traditional Chinese version). Machine learning recommendation output screen (machine learning suggestion group only) displaying the personalized recommendation generated from participant inputs. Full English translations are provided in Multimedia Appendix 6.

Data collected during IC-1 (baseline demographics and clinical history), IC-2 (IPSS urinary symptom assessment), and IC-6 (IPPI values clarification) were completed within the same web-based interface and were used as inputs to the deployed ML model. After finishing IC-1-IC-6, in the RCT (n=367), participants randomized to the MLSG received a personalized, on-screen recommendation at IC-7 (~2 min) via this dedicated interface. Figure 2 presents representative screenshots of the user interface (original traditional Chinese version), including the integrated data-entry and values-clarification screens (Figure 2) and the recommendation output screen (Figure 3). To support international readability and replicability, full English translations of all interface text and items are provided in Multimedia Appendix 6. The interface displayed the recommendation with the exact wording: “Your most likely choice is: [Immediately accept screening]” or “[Reconsider or refuse to undergo screening].” Clinicians conveyed this result using standardized, age-appropriate language, explicitly emphasizing its nature as an advisory “second opinion” based on the choices of similar patients, intended to support rather than replace the patient’s autonomy.

#### Staff Training and Fidelity

To ensure digital equity and protocol adherence, the intervention was delivered in a facilitated setting. Prior to the study, all participating medical staff completed a comprehensive 10-minute training session focused on two key objectives: (1) technical proficiency in navigating the web-based decision aid to act as effective “digital navigators” and (2) adherence to a neutral facilitation protocol to avoid influencing participant decisions. Staff provided standardized, one-on-one technical assistance (eg, interface navigation and font enlargement) to any participant experiencing difficulty. This support was strictly operational, ensuring accessibility for older adults with varying technological proficiency while maintaining the neutrality of the SDM process. Periodic fidelity checks were conducted by the principal investigator to ensure standardized delivery.

Finally, all participants completed psychological measures (IC-8; ~4 min; STAI, SA, and DCS) and made their screening decision at IC-9 (self-paced).

### Model Construction and Validation

#### Algorithm Selection and Optimization

To identify the optimal predictive model, we systematically benchmarked 5 baseline algorithms—logistic regression (LGR), random forest (RF), support vector machine, Extreme Gradient Boosting, and multilayer perceptron—alongside a deep neural network benchmark. All candidate models were evaluated using a rigorous 0.632 bootstrap protocol (n=507 iterations) to generate out-of-bag estimates of accuracy, sensitivity, specificity, and AUC. Technical specifications, including the hyperparameter optimization strategy (using design of experiments and ant lion optimizer), the full deep neural network architecture, and extended performance comparisons across all classifiers, are provided in Multimedia Appendix 7.

#### Model Deployment

Based on the comparative evaluation detailed in Multimedia Appendix 8, the RF model demonstrated the most robust performance, achieving the highest mean AUC of 0.933 (95% CI 0.902‐0.963). Consequently, it was selected as the optimal algorithm for deployment. Using a standard probability threshold of 0.5 to generate binary recommendations (“accept” vs “not now”), the deployed model achieved a sensitivity of 0.69 and a specificity of 0.97. This high specificity was particularly valued to minimize false positive encouragement of screening, thereby reducing potential overdiagnosis. This finalized RF model was integrated into a secure web server (Shiny interface; Multimedia Appendix 6) to generate personalized recommendations for the intervention group.

#### Input Features and Outcome Definition

The deployed model used 17 input features derived from the MEG dataset (IC-1 to IC-6), including age, marital status, education, prior PSA knowledge, clinical history (eg, family history, prior biopsy), and IPPI-derived value priorities. Missing data were handled using complete case analysis, as the percentage of missing values was negligible (<1%) due to the forced-entry design of the digital interface. The prediction target was the participant’s stated preference (“accept” vs “not now”) recorded immediately after the values-clarification exercise but before viewing any ML recommendation, thus serving as an unbiased ground truth for supervised learning. Detailed definitions for all predictor variables are provided in Multimedia Appendix 9.

### Statistical Analysis and Outcome Interpretation

#### Variable Scoring and Interpretation

To ensure consistent interpretation of group differences across psychological and decision-quality measures, scale directionality was defined as follows. For STAI, item direction varies: for negative affect items (STAI-2 tense, STAI-3 upset, STAI-6 worried), lower scores indicate lower anxiety; whereas for positive affect items (STAI-1 calm, STAI-4 relaxed, STAI-5 reassured), higher scores indicate greater calmness and reassurance. For the DCS, items were reverse scored (0‐4; strongly agree=0, strongly disagree=4) such that higher scores indicate greater decisional conflict (worse decision quality). For Satisfaction with Decision (Sa), items were coded such that lower scores indicate greater satisfaction.

#### Statistical Methods

Continuous variables were compared using Student *t* tests, and categorical variables using *χ*^2^ tests. To address multiplicity, we prioritized the total DCS score as the single primary end point; therefore, no adjustment was applied for this hypothesis. All other analyses, including subscales of the DCS, STAI, and Sa, were considered secondary and exploratory. For item-level comparisons within these scales, we controlled the false discovery rate using the Benjamini-Hochberg procedure to minimize type I errors. Adjusted *P* values are reported for these multiple comparisons.

Effect sizes were reported as Cohen *d* for continuous outcomes. Mean differences (MD) with 95% CIs were calculated. Statistical significance was defined as a 2-tailed *P*<.05. Analyses were performed using SPSS version 21.0 (IBM Corp) and R version 3.4.2 (R Core Team) (R package provided in Multimedia Appendix 10).

#### System Stability and Quality Control

The ML-based decision aid was deployed on a dedicated Shiny server. All intervention components (video content, questionnaires, IPPI interface, and the ML recommendation algorithm) were finalized and locked before randomization. No content modifications, algorithm updates, or bug fixes were implemented after the trial commenced. Study personnel remained consistent throughout the enrollment period, and no unexpected system failures affected data collection.

## Results

### Demographic Characteristics of the MEG Group

**Figure 4.**
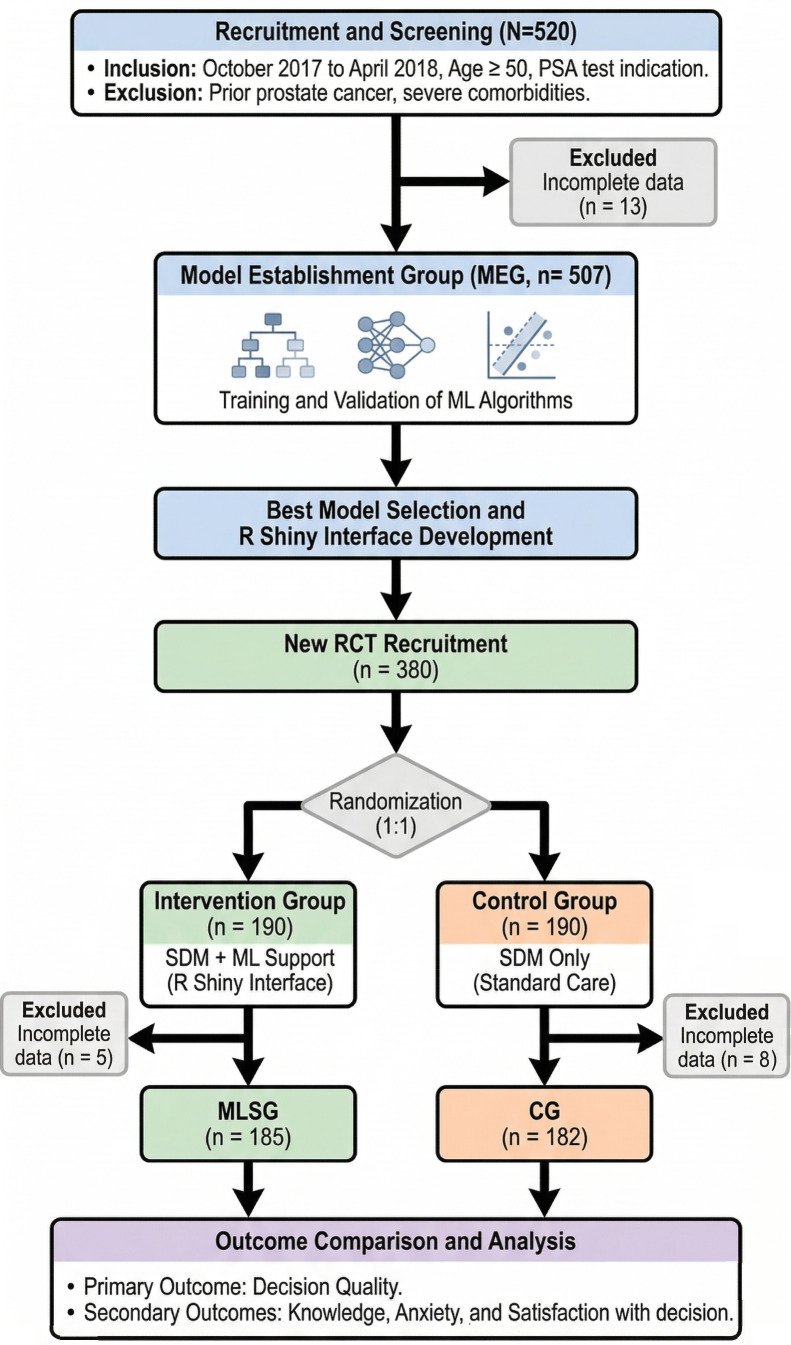
Flowchart of the SDM modeling research process. CG: control group; MEG: model establishment group; ML: machine learning; MLSG: machine learning suggestion group; PSA: prostate-specific antigen; RCT: randomized controlled trial; SDM: shared decision-making.

Baseline characteristics of the MEG cohort (n=507) are shown in Figure 4 and summarized in Table 1, with comprehensive results provided in Multimedia Appendix 11. Self-reported knowledge of PSA testing (KnowPSA score) was significantly higher in the accept group (mean 2.23, SD 0.81) than in the not now group (mean 2.07, SD 0.69; *P*=.05). Marital status distribution also differed significantly between the 2 groups (*P*<.001), with a notably higher proportion of married individuals in the accept group. Regarding the IPSS, the accept group reported significantly higher scores for IPSS items 1‐5 (*P* values ≤.02) and item 7 (*P*<.001). Furthermore, a significant difference was observed in the IPSS Q3 (quality of life) score (*P*=.008), whereas no significant difference was found for IPSS item 6. For the IPPI questionnaire, the accept group scored significantly higher across all domains except for domain G (*P*=.34).

**Table 1.** Baseline characteristics in the MEG^a^ group.

Variables	MEG group			*P* value
	All (n=507)	Accept (n=130)	Not now (n=377)	
Age (y), mean (SD)	62.89 (9.70)	63.63 (9.41)	62.63 (9.80)	.31
KnowPSA^b^, mean (SD)	2.11 (0.73)	2.23 (0.81)	2.07 (0.69)	.05
Marriage, n (%)				<.001
Married	447 (88.16)	110 (84.62)	337 (89.39)	
Divorce	5 (0.98)	5 (3.85)	0 (0)	
Single	18 (3.55)	9 (6.92)	9 (2.39)	
Widow	37 (7.30)	6 (4.62)	31 (8.22)	
PCaFH^c^, n (%)				.05
Yes	404 (79.68)	96 (73.85)	308 (81.70)	
No	103 (20.32)	34 (26.15)	69 (18.30)	
IPSS^d^, mean (SD)				
IPSS 1	0.86 (1.21)	1.31 (1.48)	0.70 (1.06)	<.001
IPSS 2	0.89 (1.21)	1.33 (1.42)	0.74 (1.09)	<.001
IPSS 3	0.78 (1.25)	1.20 (1.53)	0.64 (1.10)	.008
IPSS 4	0.78 (1.21)	1.15 (1.46)	0.66 (1.08)	.02
IPSS 5	0.53 (1.02)	0.87 (1.26)	0.42 (0.89)	<.001
IPSS 6	0.64 (1.10)	0.81 (1.28)	0.58 (1.02)	.06
IPSS 7	1.71 (1.16)	2.02 (1.29)	1.60 (1.10)	<.001
IPSS Q3^e^	5.12 (1.33)	4.85 (1.45)	5.21 (1.28)	.008
IPPI^f^, mean (SD)				
A	3.39 (1.27)	3.93 (1.07)	3.20 (1.29)	<.001
B	3.35 (1.35)	3.60 (1.26)	3.26 (1.37)	<.001
C	3.67 (1.17)	3.88 (1.15)	3.60 (1.17)	.006
D	3.14 (1.54)	3.42 (1.35)	3.05 (1.59)	.001
E	3.60 (1.26)	4.43 (0.85)	3.31 (1.26	<.001
F	3.27 (1.48)	4.16 (1.00)	2.96 (1.49)	<.001
G	2.26 (1.56)	2.29 (1.37)	2.25 (1.62)	.34
H	2.86 (1.57)	2.94 (1.26)	2.84 (1.67)	.004
I	3.53 (1.40)	3.71 (1.25)	3.47 (1.44)	.005
J	3.12 (1.56)	3.43 (1.29)	3.01 (1.63)	<.001
Top 3 ranked decision-making factors (A-J)^g^, n (%)				
First				
A	88 (17.35)	32 (24.62)	56 (14.85)	.01
B	85 (16.76)	10 (7.69)	75 (19.89)	.001
D	42 (8.28)	3 (2.31)	39 (10.34)	.004
E	32 (6.31)	19 (14.62)	13 (3.45)	<.001
Second				
B	82 (16.17)	18 (13.85)	64 (16.98)	.40
E	43 (8.48)	27 (20.77)	16 (4.24)	<.001
F	29 (5.72)	17 (13.08)	12 (3.18)	<.001
H	47 (9.27)	4 (3.08)	43 (11.41)	.004
Third				
C	112 (22.09)	28 (21.54)	84 (22.28)	.86
E	28 (5.52)	13 (10.00)	15 (3.98)	.009
F	28 (5.52)	19 (14.62)	9 (2.39)	<.001
J	65 (12.82)	9 (6.92)	56 (14.85)	.01

a MEG: model establishment group.

b KnowPSA: How would you rate your level of knowledge regarding prostate-specific antigen screening?

c PCaFF: prostate cancer among family or close friends.

d IPSS: International Prostate Symptom Score.

e Q3: quality of life.

f IPPI: importance for physiological and psychological impact.

g Decision factors derived from the Importance for IPPI questionnaire.

Regarding decision-making priorities, item A was most frequently identified as the primary factor in the accept group (n=32, 24.62%), which was significantly higher than in the not now group (14.85%; *P*=.01). In contrast, item B was more commonly prioritized as the first concern by the not now group (19.89%) compared to the accept group (7.69%; *P*=.001). Significant between-group differences were also noted for items D (*P*=.004) and E (*P*<.001). For the second most important factor, item E was significantly more endorsed by the accept group (20.77%) than the not now group (4.24%; *P*<.001), a trend also observed for item F (13.08% vs 3.18%; *P*<.001). Conversely, item H was less favored in the accept group (3.08%) compared to the not now group (11.41%; *P*=.004).

As for the third most important factor, item C was the leading choice overall (22.09%). However, item F was significantly emphasized in the accept group (14.62%) relative to the not now group (2.39%; *P*<.001). Additionally, significant between-group variations were observed for items E (*P*=.009) and J (*P*=.01). These findings highlight the differential influence of items A, B, C, E, F, and J on decision-making, suggesting nuanced variations in patient priorities as captured by the IPPI.

**Figure 5.**
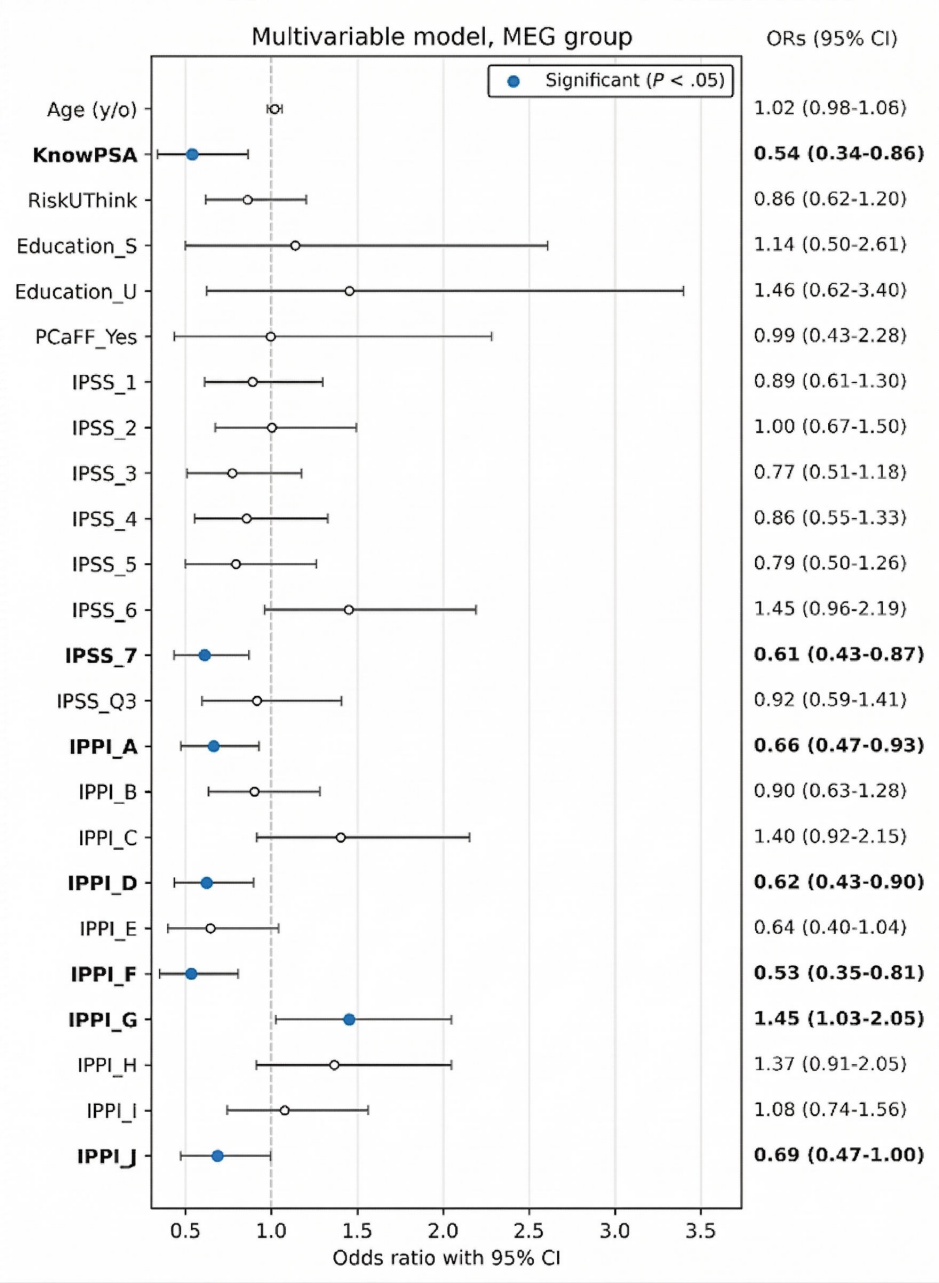
Forest plot of multivariate analysis among MEG group. IPPI: importance for physiological and psychological impact; IPSS: International Prostate Symptom Score; MEG: model establishment group; OR: odds ratio; PSA: prostate-specific antigen.

Figure 5 shows the multivariable LGR results for participants in the MEG group, presenting odds ratios (ORs) and 95% CIs for each covariate, with PSA screening uptake as the outcome. Statistically significant estimates (*P*<.05) are highlighted in blue, whereas nonsignificant estimates are shown as hollow markers. In this model, KnowPSA (OR 0.54, 95% CI 0.34‐0.86; *P*=.01) and IPSS_7 (OR 0.61, 95% CI 0.43‐0.87; *P*=.006) were statistically significant. Moreover, several IPPI subscales were also significantly associated with the outcome, including IPPI_A (OR 0.66, 95% CI 0.47‐0.93; *P*=.02), IPPI_D (OR 0.62, 95% CI 0.43‐0.90; *P*=.01), IPPI_F (OR 0.53, 95% CI 0.35‐0.81; *P*=.003), IPPI_G (OR 0.53, 95% CI 0.35‐0.81; *P*=.003), and IPPI_J (OR 0.69, 95% CI 0.47‐1.00; *P*=.048). Together, these results suggest that both knowledge-related and psychological factors are associated with PSA screening decisions in this group. Among the significant predictors, KnowPSA, IPPI_A, IPPI_D, IPPI_F, and IPPI_G had the smallest ORs, and their 95% CIs were entirely below 1, supporting their independent associations with the outcome. Overall, these findings underscore the importance of addressing both informational and psychological dimensions when supporting patient decision-making in the context of ML-enhanced guidance.

### Demographic Characteristics of the RCT Group

A total of 367 participants were included in the analysis, consisting of 185 in the MLSG group and 182 in the CG group (Table 2). Detailed results are provided in Multimedia Appendix 12. Regarding lower urinary tract symptoms measured by the IPSS, significant between-group differences were observed in specific domains. Specifically, scores for IPSS item 3 (0.93±1.28 vs 0.63±0.88; *P*=.008) and item 4 (0.93±1.33 vs 0.58±0.85; *P*=.003) were significantly higher in the MLSG group compared with the CG group.

**Table 2.** Baseline characteristics in the RCT^a^ group.

Variables	RCT group			*P* value
	All (n=367)	MLSG^b^ (n=185)	CG^c^ (n=182)	
IPSS^d^, mean (SD)				
IPSS 1	0.76 (1.16)	0.86 (1.28)	0.65 (1.02)	.08
IPSS 2	0.90 (1.32)	0.95 (1.38)	0.86 (1.26)	.54
IPSS 3	0.78 (1.11)	0.93 (1.28)	0.63 (0.88)	.008
IPSS 4	0.76 (1.13)	0.93 (1.33)	0.58 (0.85)	.003
Top 3 ranked decision-making factors (A-J)^e^, n (%)				
First				
A	114 (31.06)	46 (25.27)	68 (36.76)	.01
B	48 (13.08)	30 (16.48)	18 (9.73)	.06
G	11 (3.00)	2 (1.10)	9 (4.86)	.03
Second				
E	73 (19.89)	27 (14.84)	46 (24.86)	.01
Third				
F	84 (22.89)	37 (20.33)	47 (25.41)	.24

a RCT: randomized controlled trial.

b MLSG: machine learning suggestion group.

c CG: control group.

d IPSS: International Prostate Symptom Score.

e Decision factors derived from the importance for physiological and psychological impact questionnaire.

When ranking primary decision-making concerns, item A was most frequently identified as the top priority overall (31.06%). Notably, a significantly larger proportion of the CG group selected item A as their first concern compared with the MLSG group (36.76% vs 25.27%; *P*=.01). Regarding subsequent priorities, item E was the most common second-ranked choice overall (19.89%), while item F was the most frequently selected third-ranked priority (22.89%).

Table 3 summarizes the impact of ML-based recommendations on participants’ final decision-making outcomes. In the CG, 24.18% of participants selected “accept” as their final decision, while 75.82% chose “not now.” In contrast, participants in the MLSG who received an “accept” recommendation were significantly more likely to select “accept” compared to those in the CG (50.75% vs 24.18%; *P*<.001).

**Table 3.** Effects of machine learning suggestions on final decision.

	Final decision		*P* value
	Accept	Not now	
MLSG^a^ (decision aid suggestion)			<.001
Accept	34 (50.75)	33 (49.25)	
Not now	21 (17.80)	97 (82.20)	
CG^b^	44 (24.18)	138 (75.82)	—^c^

a MLSG: machine learning suggestion group.

b CG: control group.

c Not applicable.

Furthermore, when the ML system suggested “not now,” only 17.8% of participants chose to “accept,” which was lower than the baseline acceptance rate in the CG. These findings indicate that ML recommendations exerted a statistically significant influence on participants’ decisions, substantially increasing the likelihood of selecting the recommended option.

### Psychological Outcomes, Decision Satisfaction, and Decision Support Between MLSG and CG

For interpretability, lower scores on the DCS and Sa scale reflect better outcomes (ie, reduced conflict and enhanced satisfaction). Regarding the primary outcome, the total DCS score was significantly lower in the MLSG compared with the CG (MD –3.77, 95% CI –5.55 to −1.99; Cohen *d*=–0.44; *P*<.001), indicating superior overall decision quality. Item-level analysis revealed significant improvements in several key dimensions: participants in the MLSG reported greater perceived decision support (DCS7: adjusted *P*=.03), more adequate advice (DCS9: adjusted *P*<.001), higher decision confidence (DCS10: adjusted *P*=.03; DCS11: adjusted *P*<.001), and found the decision-making process significantly easier (DCS12: adjusted *P*<.001).

For decision satisfaction, the MLSG demonstrated a large and statistically significant improvement in the total Sa score (MD –7.38, 95% CI –8.54 to −6.18; *d*=–1.29; *P*<.001). Notably, the MLSG reported significantly higher satisfaction across all 5 items (adjusted *P* values <.001, Cohen *d* ≤ –1.10). These gains were most pronounced in information adequacy (Sa1: MD −1.46, 95% CI –1.72 to −1.20), confidence that the decision was the best personal option (Sa2), alignment with personal values (Sa3), and a stronger intention to implement the chosen decision (Sa4).

Regarding anxiety, although the total STAI score did not differ significantly between groups (*P*=.37), item-level analysis identified significant improvements in specific psychological states. Participants in the MLSG reported feeling significantly less worried (STAI 6: MD –0.98, 95% CI –1.20 to −0.76; *d*=–0.89; adjusted *P*<.001) and significantly calmer (STAI 1: MD 0.30, 95% CI 0.06 to 0.54; *d*=0.25; adjusted *P*=.01) compared with the CG. Detailed item-level comparisons are presented in Table 4.

**Table 4.** Comparison of the STAI^a^, Sa^b^, and DSC^c^ questionnaires between the MLSG^d^ and CG^e^.

Variables	RCT^f^ group			*P* value	Cohen *d*	Adjusted *P*^g^
	MLSG (n=185), mean (SD)	CG (n=182), mean (SD)	MD^h^ (95% CI)			
Anxiety						
STAI 1	2.28 (1.20)	1.98 (1.14)	0.30 (0.06 to 0.54)	.004	0.25	*.01*
STAI 2	2.22 (0.98)	2.08 (0.90)	0.14 (–0.05 to 0.33)	.14	0.15	.17
STAI 3	2.21 (1.09)	2.36 (1.05)	–0.15 (–0.37 to 0.07)	.17	–0.14	.17
STAI 4	2.15 (1.19)	1.97 (1.13)	0.18 (–0.06 to 0.42)	.06	0.15	.10
STAI 5	2.12 (1.21)	1.87 (1.10)	0.25 (0.01 to 0.49)	.03	0.21	.06
STAI 6	2.00 (1.02)	2.98 (1.16)	–0.98 (–1.20 to -0.76)	<.001	–0.89	*<.001*
Total	12.97 (2.39)	13.23 (3.13)	–0.26 (–0.83 to 0.31)	.37	–0.09	—^i^
Decision satisfaction						
Sa 1	1.75 (0.92)	3.21 (1.56)	–1.46 (–1.72 to -1.20)	<.001	–1.14	*<.001*
Sa 2	1.83 (0.88)	3.29 (1.44)	–1.46 (–1.71 to –1.21)	<.001	–1.22	*<.001*
Sa 3	1.77 (0.89)	3.19 (1.60)	–1.42 (–1.69 to –1.15)	<.001	–1.10	*<.001*
Sa 4	1.67 (0.82)	3.13 (1.69)	–1.46 (–1.73 to –1.19)	<.001	–1.10	*<.001*
Sa 5	1.57 (0.81)	3.12 (1.67)	–1.55 (–1.82 to –1.28)	<.001	–1.18	*<.001*
Total	8.58 (3.02)	15.94 (7.52)	–7.38 (–8.54 to –6.18)	<.001	–1.29	—
Decision conflicts						
DCS 1	2.09 (0.96)	2.29 (1.11)	–0.20 (–0.41 to 0.01)	.14	–0.19	.22
DCS 2	2.23 (1.02)	2.18 (1.15)	0.05 (–0.17 to 0.27)	.32	0.05	.40
DCS 3	2.24 (1.09)	2.19 (1.09)	0.05 (–0.17 to 0.27)	.65	0.05	.65
DCS 4	2.23 (1.09)	2.16 (1.18)	0.07 (–0.16 to 0.30)	.37	0.06	.43
DCS 5	2.17 (1.08)	2.33 (1.24)	–0.16 (–0.40 to 0.08)	.32	–0.14	.40
DCS 6	2.29 (1.08)	2.42 (1.25)	–0.13 (–0.37 to 0.11)	.43	–0.11	.46
DCS 7	1.85 (1.05)	2.19 (1.24)	–0.34 (–0.58 to –0.10)	.01	–0.30	*.03*
DCS 8	2.05 (1.17)	1.91 (1.06)	0.14 (–0.09 to 0.37)	.31	0.12	.40
DCS 9	1.73 (0.95)	2.30 (1.17)	–0.57 (–0.79 to –0.35)	<.001	–0.56	*<.001*
DCS 10	1.88 (0.99)	2.26 (1.26)	–0.38 (–0.61 to –0.15)	.008	–0.33	*.03*
DCS 11	1.81 (0.98)	2.38 (1.22)	–0.57 (–0.80 to -0.34)	<.001	–0.51	*<.001*
DCS 12	1.81 (0.93)	2.43 (1.23)	–0.62 (-0.84 to –0.40)	<.001	–0.57	*<.001*
DCS 13	1.89 (1.05)	2.12 (1.17)	–0.23 (–0.46 to –0.00)	.07	–0.21	.15
DCS 14	1.96 (0.95)	2.15 (1.06)	–0.19 (–0.40 to 0.02)	.10	–0.19	.19
DCS 15	1.78 (0.87)	2.08 (1.16)	–0.30 (–0.51 to –0.09)	.04	–0.29	.09
DCS 16	1.60 (0.73)	1.98 (1.09)	–0.38 (–0.57 to –0.19)	.004	–0.41	*.01*
Total	31.60 (9.29)	35.37 (7.98)	–3.77 (–5.55 to –1.99)	<.001	–044	—

a STAI: Spielberger State-Trait Anxiety Inventory.

b Sa: Satisfaction with Decision.

c DCS: Decisional Conflict Scale.

d MLSG: machine learning suggestion group.

e CG: control group.

f RCT: randomized controlled trial.

g *P* values adjusted for multiplicity using the Benjamini-Hochberg false discovery rate method within each outcome (anxiety, decision satisfaction, decisional conflict). Italics values indicate statistical significance after adjustment (adjusted *P*<.05).

h MD: mean difference.

i Not applicable.

j Scale direction: For DCS and Sa, lower scores indicate better outcomes (ie, lower decisional conflict and higher decision satisfaction). For STAI, item direction varies: for STAI-6 (worry), lower scores indicate lower anxiety; whereas for STAI-1 (calm) and STAI-5 (reassured), higher scores indicate greater calmness and reassurance.

## Discussion

### Principal Findings

In this randomized controlled trial of 367 men aged 50‐75 years, adding personalized ML recommendations to a structured SDM workflow was associated with significantly improved decision quality for PSA screening. Supported by a high-performing prediction model (AUC 0.93), the intervention group (MLSG) reported lower decisional conflict and reduced anxiety compared with standard care. Specifically, participants receiving ML-supported recommendations experienced greater calmness (STAI 1: adjusted *P*=.01) and less worry (STAI 6: adjusted *P*<.001). Beyond anxiety reduction, the intervention produced large, consistent improvements in decision satisfaction across all domains (Cohen *d* >1.10; adjusted *P* values <.001), including perceived adequacy of information and alignment with personal values.

Importantly, the intervention was designed to complement—rather than replace—clinician counseling. The ML output was presented as an advisory “second opinion” and reinforced by standardized clinician communication, ensuring that the final screening decision remained patient-directed. This approach is particularly relevant for older adults, who often face cognitive load and uncertainty when weighing the complex trade-offs of PSA screening [32].

### Comparison With Prior Work

Although conventional prediction approaches (eg, LGR) can identify determinants associated with PSA screening intentions and uptake, they are generally not designed to deliver individualized, real-time recommendations at the point of decision-making [33]. Unlike prior decision-aid evaluations that focused primarily on presentation format or generic personalization, our intervention explicitly targeted decisional conflict and anxiety [34,35]—key barriers to informed choice in adults and aging populations [3,36]. Prior studies also suggest that education, age, symptom-related, and psychosocial factors influence screening behavior, and that incorporating constructs such as self-efficacy and emotional well-being may improve the use of decision support tools [37-39]. Watson et al [33] reported that men’s intention to undergo PSA testing was closely related to baseline knowledge, attitudes, and perceived risk, underscoring the central role of informational and psychological determinants.

Consistent with this literature, our multivariable LGR analysis in the MEG cohort confirmed that screening decisions were driven by a complex interplay of informational and preference-related factors. Specifically, higher PSA-related knowledge (KnowPSA) was independently associated with lower screening uptake (OR 0.54; *P*=.01). This inverse association suggests that participants with limited understanding may be more inclined to accept screening without fully weighing potential harms (eg, false positives, overdiagnosis), whereas greater awareness appears to foster a more cautious approach, reducing uninformed compliance.

In addition to knowledge, clinical symptoms and personal values were robust predictors of decision-making. Greater urinary symptom severity (IPSS item 7; OR 0.61; *P*=.006) was associated with lower screening uptake, possibly reflecting a prioritization of symptom management over preventive screening in symptomatic individuals. Furthermore, several values-clarification subscales (IPPI items A, D, F, G, and J) significantly predicted screening behavior, indicating that participants who placed higher importance on specific physiological, psychological, or social impacts were more likely to decline or defer screening.

These results suggest that when patients are equipped with sufficient knowledge and an opportunity to clarify their values, they may become more discerning, potentially leading to informed decisions to decline screening based on their personal context. This aligns with the core tenet of SDM, which prioritizes value-concordant choices over passive adherence. The robust predictive power of these factors underscores the use of our ML model in capturing nuanced, individualized determinants, thereby moving beyond generic guidelines to support truly patient-centered care.

### Clinical Implications

Recent evidence suggests that fewer than 25% of men aged 40 and older report discussing the benefits and harms of PSA testing with their providers, a gap even more pronounced among those with lower educational attainment [40]. The selection of the delivery mode for DAs is critical to addressing this disparity. Given that the target population for prostate cancer screening primarily consists of older adults, web-based DAs may present usability challenges due to age-related declines in fine motor control or limited digital literacy [41]. To mitigate these barriers, our intervention used a facilitated delivery approach. By providing personalized, accessible recommendations through a supported interface, our ML-driven decision aid ensures that screening decisions align with individual values rather than being driven by incomplete information or physician preference alone. Compared with conventional decision aids, ML recommendations significantly enhanced decisional confidence and yielded modest yet consistent increases in screening acceptance. This aligns with prior evidence that technology-supported tools can shape patient preferences and improve decision quality [24,42].

However, we emphasize that ML should be viewed as a decision-support tool, not a decision-maker. The modest increase in screening acceptance observed in the MLSG suggests that the tool facilitated decisive action, but ethical implementation requires ensuring that this influence does not coerce patients. Future integration into routine practice must prioritize transparency and physician training to maintain the delicate balance between algorithmic guidance and patient autonomy.

Taken together, these findings indicate that embedding personalized, ML-driven recommendations within SDM frameworks may not only improve decision quality but also enhance psychological outcomes. Before integrating ML-assisted tools into routine clinical practice, further research is warranted to evaluate their psychological and physiological safety. Such investigations are essential to determine not only the efficacy of ML recommendations in enhancing decision quality but also their broader implications for patient autonomy and well-being [43].

### Limitations

Several limitations should be noted. First, while we provided uniform technical assistance, we did not formally assess baseline eHealth literacy or track the frequency of support required, limiting our ability to analyze effects based on digital proficiency. Future studies should incorporate validated measures like the eHealth Literacy Scale. Second, although our model demonstrated high discrimination (AUC >0.90), calibration metrics (eg, Brier score) were not assessed during the development phase; future work should include calibration plots to ensure risk estimates are accurate across subgroups. Third, unmeasured confounding remains a possibility, as factors such as physician influence or access barriers were not fully captured.

Finally, the current user interface provides binary recommendations (agree vs not now) without visualizing feature contributions. Integrating Explainable Artificial Intelligence techniques, such as Shapley Additive Explanations values, will be critical for future iterations to enhance transparency and trust.

### Conclusion

This study demonstrates that integrating personalized ML recommendations into SDM frameworks can enhance decision quality while simultaneously reducing psychological burdens, such as anxiety and decisional conflict. Participants exposed to ML-supported recommendations reported greater confidence, reassurance, and satisfaction with their choices, underscoring the unique psychological value of individualized algorithmic guidance in prostate cancer screening.

Importantly, these findings suggest that ML-based decision support should be regarded as a complement to, rather than a replacement for, traditional decision aids and physician-patient discussions. By enriching the decision-making process with nuanced, personalized insights, ML has the potential to foster more supportive and less distressing decision environments.

## Supplementary material

10.2196/83238 Multimedia Appendix 1 Importance for physiological and psychological impact questionnaire.

10.2196/83238 Multimedia Appendix 2 International Prostate Symptom Score questionnaire.

10.2196/83238 Multimedia Appendix 3 Decisional Conflict Scale questionnaire.

10.2196/83238 Multimedia Appendix 4 Expert validity review questionnaire.

10.2196/83238 Multimedia Appendix 5 Knowledge test items.

10.2196/83238 Multimedia Appendix 6 Website link and interface.

10.2196/83238 Multimedia Appendix 7 Technical details of model development.

10.2196/83238 Multimedia Appendix 8 Performance evaluation of machine learning models.

10.2196/83238 Multimedia Appendix 9 Variables description.

10.2196/83238 Multimedia Appendix 10 Detail baseline characteristics in the model establishment group.

10.2196/83238 Multimedia Appendix 11 R packages.

10.2196/83238 Multimedia Appendix 12 Detail baseline characteristics in the randomized controlled trial group.

10.2196/83238 Checklist 1 CONSORT-EHEALTH checklist.

## Abbreviations

AUC: area under the curve
CG: control group
CONSORT: Consolidated Standards of Reporting Trials
DA: decision aid
DCS: Decisional Conflict Scale
IPPI: importance for physiological and psychological impact
IPSS: International Prostate Symptom Score
LGR: logistic regression
MD: mean difference
MLSG: machine learning suggestion group
OR: odds ratio
PSA: prostate-specific antigen
RCT: randomized controlled trial
RF: random forest
Sa: Satisfaction with Decision
SDM: shared decision-making
STAI: Spielberger State-Trait Anxiety Inventory
