# Supplementary material for: Web-Based Personalized Machine Learning Recommendations to Enhance Shared Decision-Making in Prostate-Specific Antigen Screening: Randomized Controlled Trial
Source: JMIR Aging. 2026 Apr 13;9:e83238. doi: 10.2196/83238 (PMC13075628; doi:10.2196/83238)
Supplement: Multimedia Appendix 1 [file aging-v9-e83238-s001.docx]

**Appendix 1. Importance for Physiological and Psychological Impact (IPPI)**

Considering the option you prefer, please answer the following questions:

| **Part 1** | | | | | |
| --- | --- | --- | --- | --- | --- |
| 1. How familiar are you with prostate-specific antigen (PSA) screening? | □ Very familiar | □ Somewhat familiar | □ Not at all familiar | □ Disagree | □ Strongly Disagree |
| 1. Have you ever heard any information about prostate cancer? | □ Very familiar | □ Somewhat familiar | □ Not at all familiar | □ Disagree | □ Strongly Disagree |
| 1. Compared to others, how do you perceive your risk of developing prostate cancer? | □ Much higher | □ Slightly higher | □ About the same | □ Slightly lower | □ Much lower |

| **Part 2** | | | |
| --- | --- | --- | --- |
| 1. A normal screening result does not necessarily mean there is no cancer; an abnormal screening result does not necessarily mean there is cancer. | □ True | □ False | □ Do not know |
| 1. Most prostate cancers grow slowly, and some cancers detected through screening may never cause symptoms even without treatment. | □ True | □ False | □ Do not know |
| 1. Even if the result of a prostate biopsy is normal, prostate cancer may still be present. | □ True | □ False | □ Do not know |

| **Part 3-1_Physical Impact** | 0  (Not important at all) | 1 | 2 | 3 | 4 | 5  (Extremely important) |
| --- | --- | --- | --- | --- | --- | --- |
| 1. Positive Physical Impact: After screening, there is approximately a 2% chance of early diagnosis and early treatment, which may prolong life. How important is this impact to you? | □ 0 | □ 1 | □ 2 | □ 3 | □ 4 | □ 5 |
| 1. After screening, there is approximately a 12% chance that, due to limited test accuracy, you may receive an abnormal result even if you do not have cancer. This could lead to unnecessary further examinations and repeated biopsies, resulting in physical risk. How important is this impact to you? | □ 0 | □ 1 | □ 2 | □ 3 | □ 4 | □ 5 |
| 1. After screening, there is approximately a 13% chance that, despite a normal test result, you may actually have early-stage cancer, leading to delayed treatment. How important is this impact to you? | □ 0 | □ 1 | □ 2 | □ 3 | □ 4 | □ 5 |
| 1. After screening, there is approximately a 1% chance of detecting a slow-growing cancer that would never cause symptoms in your lifetime, but this finding may result in unnecessary major surgery or radiation therapy. How important is this impact to you? | □ 0 | □ 1 | □ 2 | □ 3 | □ 4 | □ 5 |

| **Part 3-2_Psychological Impact** | 0  (Not important at all) | 1 | 2 | 3 | 4 | 5  (Extremely important) |
| --- | --- | --- | --- | --- | --- | --- |
| 1. After screening, you will know your PSA level, which satisfies your need for knowledge about your health status. How important is this impact to you | □ 0 | □ 1 | □ 2 | □ 3 | □ 4 | □ 5 |
| 1. After screening, there is approximately an 85% chance that the result will be normal, which may provide you with peace of mind. How important is this impact to you? | □ 0 | □ 1 | □ 2 | □ 3 | □ 4 | □ 5 |
| 1. Most participants experience mild nervousness before screening. How important is this impact to you? | □ 0 | □ 1 | □ 2 | □ 3 | □ 4 | □ 5 |
| 1. After screening, there is approximately a 15% chance that the result will be abnormal, which may cause you to feel anxious. How important is this impact to you? | □ 0 | □ 1 | □ 2 | □ 3 | □ 4 | □ 5 |
| 1. After screening, there is approximately a 13% chance that, despite a normal test result, you actually have cancer. If cancer is discovered later, it may cause psychological distress. How important is this impact to you? | □ 0 | □ 1 | □ 2 | □ 3 | □ 4 | □ 5 |
| 1. After screening, there is approximately a 3% chance that a subsequent biopsy will confirm cancer, and a cancer diagnosis may cause you severe anxiety. How important is this impact to you? | □ 0 | □ 1 | □ 2 | □ 3 | □ 4 | □ 5 |

| **Part 4** | | | | | | | | | | |
| --- | --- | --- | --- | --- | --- | --- | --- | --- | --- | --- |
| Which item do you consider the most important? | □ A | □ B | □ C | □ D | □ E | □ F | □ G | □ H | □ I | □ J |
| Which item do you consider the second most important? | □ A | □ B | □ C | □ D | □ E | □ F | □ G | □ H | □ I | □ J |

| **Part 5** |
| --- |
| **My decision is**  □ I choose to undergo screening □ I choose not to undergo screening □ I am still uncertain and would like more time to decide |
