# Supplementary material for: Web-Based Personalized Machine Learning Recommendations to Enhance Shared Decision-Making in Prostate-Specific Antigen Screening: Randomized Controlled Trial
Source: JMIR Aging. 2026 Apr 13;9:e83238. doi: 10.2196/83238 (PMC13075628; doi:10.2196/83238)
Supplement: Multimedia Appendix 2 [file aging-v9-e83238-s002.docx]

**Appendix 2. International Prostate Symptom Score (IPSS) Questionnaire**

Over the past month, how often have you experienced the following symptoms? (Please circle the number that best applies to you.)

| **Question** | **Not at all** | **Less than 1 in 5 times** | **Less than half the time** | **About half the time** | **More than half the time** | **Almost always** | **Your**  **score** |
| --- | --- | --- | --- | --- | --- | --- | --- |
| 1. **Incomplete emptying:** How often have you had the sensation of not emptying your bladder completely after you finished urinating? | 0 | 1 | 2 | 3 | 4 | 5 |  |
| 2. **Frequency:** How often have you had to urinate again less than two hours after you finished urinating? | 0 | 1 | 2 | 3 | 4 | 5 |  |
| 3. **Intermittency:** How often have you found you stopped and started again several times when you urinated? | 0 | 1 | 2 | 3 | 4 | 5 |  |
| 4. **Urgency:** How often have you found it difficult to postpone urination? | 0 | 1 | 2 | 3 | 4 | 5 |  |
| 5. **Weak stream:** How often have you had a weak urinary stream? | 0 | 1 | 2 | 3 | 4 | 5 |  |
| 6. **Straining:** How often have you had to push or strain to begin urination? | 0 | 1 | 2 | 3 | 4 | 5 |  |
|  | **None** | **1Times** | **2Times** | **3Times** | **4Times** | **5Times** |  |
| 7. **Nocturia:** How many times did you typically get up to urinate from the time you went to bed at night until the time you got up in the morning? | 0 | 1 | 2 | 3 | 4 | 5 |  |

**Quality of Life Question (QoL)**

| **Question** | **Delighted** | **Pleased** | **Mostly satisfied** | **Mixed (about equally satisfied and dissatisfied)** | **Mostly dissatisfied** | **Unhappy** | **Terrible** |
| --- | --- | --- | --- | --- | --- | --- | --- |
| If you were to spend the rest of your life with your urinary condition just the way it is now, how would you feel about that? | 0 | 1 | 2 | 3 | 4 | 5 | 6 |

**Scoring**

Mild symptoms: 0–7 points

Moderate symptoms: 8–19 points

Severe symptoms: 20–35 points
