# Supplementary material for: Web-Based Personalized Machine Learning Recommendations to Enhance Shared Decision-Making in Prostate-Specific Antigen Screening: Randomized Controlled Trial
Source: JMIR Aging. 2026 Apr 13;9:e83238. doi: 10.2196/83238 (PMC13075628; doi:10.2196/83238)
Supplement: Multimedia Appendix 3 [file aging-v9-e83238-s003.docx]

**Appendix 3. Decisional Conflict Scale (DCS)**

Considering the option you prefer, please answer the following questions:

| **Question** | **Strongly Agree** | **Agree** | **Neither Agree Nor Disagree** | **Disagree** | **Strongly Disagree** |
| --- | --- | --- | --- | --- | --- |
| 1. I know which options are available to me. | 0 | 1 | 2 | 3 | 4 |
| 1. I know the benefits of each option. | 0 | 1 | 2 | 3 | 4 |
| 1. I know the risks and side effects of each option. | 0 | 1 | 2 | 3 | 4 |
| 1. I am clear about which benefits matter most to me. | 0 | 1 | 2 | 3 | 4 |
| 1. I am clear about which risks and side effects matter most to me. | 0 | 1 | 2 | 3 | 4 |
| 1. I am clear about which is more important to me   (the benefits or the risks and side effects). | 0 | 1 | 2 | 3 | 4 |
| 1. I have enough support from others to make a choice. | 0 | 1 | 2 | 3 | 4 |
| 1. I am choosing without pressure from others. | 0 | 1 | 2 | 3 | 4 |
| 1. I have enough advice to make a choice. |  |  |  |  |  |
| 1. I am clear about the best choice for me. | 0 | 1 | 2 | 3 | 4 |
| 1. I feel sure about what to choose. | 0 | 1 | 2 | 3 | 4 |
| 1. This decision is easy for me to make. |  |  |  |  |  |
| 1. I feel I have made an informed choice. | 0 | 1 | 2 | 3 | 4 |
| 1. My decision shows what is important to me. | 0 | 1 | 2 | 3 | 4 |
| 1. I expect to stick with my decision. | 0 | 1 | 2 | 3 | 4 |
| 1. I am satisfied with my decision. | 0 | 1 | 2 | 3 | 4 |
