# Supplementary material for: Web-Based Personalized Machine Learning Recommendations to Enhance Shared Decision-Making in Prostate-Specific Antigen Screening: Randomized Controlled Trial
Source: JMIR Aging. 2026 Apr 13;9:e83238. doi: 10.2196/83238 (PMC13075628; doi:10.2196/83238)
Supplement: Multimedia Appendix 4 [file aging-v9-e83238-s004.docx]

**Appendix 4. Expert Validity Review Questionnaire**

Expert Validity Review Questionnaire for “Shared Decision-Making in Healthcare: Assisting Patients in Deciding Whether to Undergo Prostate-Specific Antigen Screening Using Decision Trees and Neural Networks”

Dear Expert,

Thank you very much for taking the time out of your busy schedule to review this questionnaire. This instrument is part of a doctoral dissertation entitled “Shared Decision-Making in Healthcare: Assisting Patients in Deciding Whether to Undergo Prostate-Specific Antigen Screening Using Decision Trees and Neural Networks.” The purpose of this study is to provide decision support through decision trees and neural networks, thereby helping participants determine whether to undergo prostate-specific antigen (PSA) screening. This approach aims to increase participants’ satisfaction with their decisions and reduce decisional conflict, as well as to decrease the number of individuals unable to reach a decision.

To establish the validity of this research tool, your valuable insights are sincerely requested. Please review the questionnaire items based on the attached research objectives, research questions, and questionnaire framework, and assess the accuracy of each item and its alignment with the study’s aims. If you find any ambiguities or unclear wording, kindly provide your suggestions for revision directly on the item. Should you feel that any item should be removed or that additional items should be included, please indicate or annotate accordingly. Your feedback will serve as an important reference for revising this instrument. We greatly appreciate your input, as your expert opinion will be of immense value to this research.

Upon completion, please submit the questionnaire. Thank you again for your generous assistance.

Sincerely,

Advisor: Dr. Ming-Chih Chen
Graduate Institute of Business Administration, Fu Jen Catholic University

Graduate Student: Yi-Ting Lin

Contact Phone: 0956578998
E-mail: [y.llingtaiwan@gmail.com](mailto:y.llingtaiwan@gmail.com)
Date: October 16, 2017

*Required Fields

1. Email Address*: _____________

1. Name of Expert *: ____________

**Abstract**

Shared decision-making (SDM) has been demonstrated to effectively enhance the alignment of medical decisions with patient values and to improve patient understanding of treatment options. However, many patients still experience a certain degree of indecision, doubt, and decisional conflict during the decision-making process. This project aims to utilize decision trees and neural networks to provide decision support for patients considering whether to undergo prostate-specific antigen (PSA) screening, with the goal of increasing decision self-efficacy and reducing decisional conflict. A total of 930 participants will be recruited. The first 30 questionnaires will be used to assess the reliability of the instrument. Data from the first 300 participants will be used to train the decision tree and neural network classifiers. After training is complete, participants will be randomly assigned to two groups: the experimental group, which will receive decision support from the trained models, and the control group, which will not. Each group will consist of approximately 300 participants. The primary outcomes will be compared between groups, including decision-efficacy, satisfaction with the decision-making process, and the degree of decisional conflict.

**Background**

SDM has been shown to enhance the congruence between medical decisions and patient values, improve patient understanding of treatment, and reduce decisional conflict. Nevertheless, even with the assistance of decision aids, many patients continue to experience uncertainty, lack of confidence, and decisional conflict. Some studies have even reported that the use of decision aids may increase decisional conflict in certain cases. Furthermore, current decision aids are often unable to address the issue of patients being unable to make a decision. Several studies on SDM for PSA screening have reported similar phenomena, with many patients remaining undecided or experiencing decisional conflict.

Decision trees and neural networks have been widely applied in clinical decision support for healthcare professionals, including in the diagnosis of diabetes, prediction of cancer prognosis, postoperative outcomes for colorectal cancer, and diagnosis of fatty liver disease. We believe these methods can be more broadly applied to patient-centered shared decision-making. By collecting a large dataset from study participants, we will train decision tree and neural network classifiers to predict patients’ decisions regarding PSA screening. According to the literature, factors influencing the decision to undergo PSA screening include age, prostate symptoms, knowledge about screening, anxiety about prostate cancer, and personal values and attitudes. This study will use these factors to develop predictive models and provide decision support. The impact of these decision aids will be evaluated using validated instruments, including the State Anxiety Inventory, Decision Self-Efficacy Scale, Decision Satisfaction Scale, Decisional Conflict Scale, and measures of value-concordant decision-making. The ultimate goal is to increase decision self-efficacy, reduce decisional conflict, and decrease the proportion of participants unable to make a decision.

**Objectives**

1. To provide decision support using decision trees and neural networks to assist patients in deciding whether to undergo PSA screening, with the aim of increasing decision self-efficacy and reducing decisional conflict.
2. To use decision trees and neural networks to reduce the proportion of participants unable to make a decision regarding PSA screening.

**Research Design and Implementation Methods**

**Step 1: Questionnaire Reliability Assessment**
The reliability of the questionnaire will be evaluated using standard psychometric methods to ensure consistency and stability of the instrument.

**Step 2: Training the Classifiers**
A total of 300 participants will be recruited for the classifier training group. Data collected from these participants—including demographic information, cancer-related anxiety, prostate symptom scores, knowledge of medical options, perceived importance of physical and psychological impacts, attitudes, decision-making, and value-congruence—will be used to train decision tree and neural network classifiers. The process for participants in the classifier training group is as follows:

- **Step 1:** Complete the decision support tool, which includes questions on medical option awareness, importance of physical and psychological impacts, attitudes, decision-making, and value congruence.
- **Step 2:** Complete the classifier training group questionnaire, which includes demographic information, cancer-related anxiety, prostate symptom scores, self-efficacy scale, decision satisfaction scale, and decisional conflict scale.

**Step 3: Testing the Impact of Decision Tree and Neural Network Classifiers on Decision-Making and Psychological Outcomes**
An additional 600 participants will be recruited and randomly assigned to two groups:

- **Experimental Group:** Participants will first receive an explanation and written decision aid from a counselor, then receive recommendations generated by the decision tree and neural network classifiers.
- **Control Group:** Participants will only receive an explanation and written decision aid from a counselor.

**Expert Validity Assessment**

***Instructions for Completion:***

1. This questionnaire consists of 18 items. Please indicate the degree to which each item is applicable by selecting the appropriate option.
2. If you find any ambiguity, awkward phrasing, inappropriate categorization, or have any suggestions for improvement, we sincerely invite your feedback to enhance the quality of the questionnaire. Thank you for your valuable input.

| **Questionnaire Part 1** |
| --- |
| The purpose of the following questions is to assess participants’ perceived knowledge about prostate cancer and screening, as well as their level of concern about developing prostate cancer, prior to receiving any decision aid.  Please evaluate whether the following three items are appropriate for assessing the above topics. |
| 1. **How familiar are you with prostate-specific antigen (PSA) screening?***   □ Very familiar □ Somewhat familiar □ Not at all familiar |
| □ Appropriate  □ Not appropriate  □ Appropriate after revision |
| Suggestions for revision_______________________ |
| 1. **Have you ever heard any information about prostate cancer? ***   □ Very familiar □ Somewhat familiar □ Not at all familiar |
| □ Appropriate  □ Not appropriate  □ Appropriate after revision |
| Suggestions for revision________________________ |
| 1. **Compared to others, how do you perceive your risk of developing prostate cancer? ***   □ Much higher □ Slightly higher □ About the same □ Slightly lower  □ Much lower |
| □ Appropriate  □ Not appropriate  □ Appropriate after revision |
| Suggestions for revision________________________ |
| **Part 2** |
| These questions aim to assess participants’ actual knowledge about prostate cancer and screening after receiving the decision aid. Please evaluate whether the following three items are appropriate for assessing the above topics. |
| 1. **A normal screening result does not necessarily mean there is no cancer; an abnormal screening result does not necessarily mean there is cancer. ***   □ True □ False □ Do not know |
| □ Appropriate  □ Not appropriate  □ Appropriate after revision |
| Suggestions for revision________________________ |
| 1. **Most prostate cancers grow slowly, and some cancers detected through screening may never cause symptoms even without treatment. ***   □ True □ False □ Do not know |
| □ Appropriate  □ Not appropriate  □ Appropriate after revision |
| Suggestions for revision________________________ |
| 1. **Even if the result of a prostate biopsy is normal, prostate cancer may still be present. ***   □ True □ False □ Do not know |
| □ Appropriate  □ Not appropriate  □ Appropriate after revision |
| Suggestions for revision________________________ |
| **Part 3-1_Physical Impact** |
| The decision aid has provided a comprehensive explanation of both the positive and negative physical and psychological impacts of prostate-specific antigen (PSA) screening.  In this section of the questionnaire, these impacts are categorized into ten items (A–J): four physical and six psychological. Participants are asked to evaluate the importance of each impact based on their personal values, with 0 indicating “not important at all” and 5 indicating “extremely important.”  Please assess whether the following four items appropriately represent the physical positive and negative impacts of screening. |
| 1. **Positive Physical** Impact: After screening, there is approximately a 2% chance of early diagnosis and early treatment, which may prolong life. How important is this impact to you? *****   □ 0 (Not important at all) □ 1 □ 2 □ 3 □ 4 □ 5 (Extremely important) |
| □ Appropriate  □ Not appropriate  □ Appropriate after revision |
| Suggestions for revision________________________ |
| 1. **Negative Physical Impact:** After screening, there is approximately a 12% chance that, due to limited test accuracy, you may receive an abnormal result even if you do not have cancer. This could lead to unnecessary further examinations and repeated biopsies, resulting in physical risk. How important is this impact to you? *****   □ 0 (Not important at all) □ 1 □ 2 □ 3 □ 4 □ 5 (Extremely important) |
| □ Appropriate  □ Not appropriate  □ Appropriate after revision |
| Suggestions for revision________________________ |
| 1. **Negative Physical Impact:** After screening, there is approximately a 13% chance that, despite a normal test result, you may actually have early-stage cancer, leading to delayed treatment. How important is this impact to you? *****   □ 0 (Not important at all) □ 1 □ 2 □ 3 □ 4 □ 5 (Extremely important) |
| □ Appropriate  □ Not appropriate  □ Appropriate after revision |
| Suggestions for revision________________________ |
| 1. **Negative Physical Impact:** After screening, there is approximately a 1% chance of detecting a slow-growing cancer that would never cause symptoms in your lifetime, but this finding may result in unnecessary major surgery or radiation therapy. How important is this impact to you? *****   □ 0 (Not important at all) □ 1 □ 2 □ 3 □ 4 □ 5 (Extremely important) |
| □ Appropriate  □ Not appropriate  □ Appropriate after revision |
| Suggestions for revision________________________ |
| **Part 3-2_Psychological Impact** |
| The decision aid has provided a comprehensive explanation of both the positive and negative physical and psychological impacts of prostate-specific antigen (PSA) screening. In this section of the questionnaire, these impacts are categorized into ten items (A–J): four physical and six psychological. Participants are asked to evaluate the importance of each impact based on their personal values, with 0 indicating “not important at all” and 5 indicating “extremely important.”  Please assess whether the following six items are appropriate representations of the psychological positive and negative impacts of screening. |
| 1. **Positive Psychological Impact:** After screening, you will know your PSA level, which satisfies your need for knowledge about your health status. How important is this impact to you? *****   □ 0 (Not important at all) □ 1 □ 2 □ 3 □ 4 □ 5 (Extremely important) |
| □ Appropriate  □ Not appropriate  □ Appropriate after revision |
| Suggestions for revision________________________ |
| 1. **Positive Psychological Impact:** After screening, there is approximately an 85% chance that the result will be normal, which may provide you with peace of mind. How important is this impact to you? *****   □ 0 (Not important at all) □ 1 □ 2 □ 3 □ 4 □ 5 (Extremely important) |
| □ Appropriate  □ Not appropriate  □ Appropriate after revision |
| Suggestions for revision________________________ |
| 1. **Negative Psychological Impact:** Most participants experience mild nervousness before screening. How important is this impact to you? *****   □ 0 (Not important at all) □ 1 □ 2 □ 3 □ 4 □ 5 (Extremely important) |
| □ Appropriate  □ Not appropriate  □ Appropriate after revision |
| Suggestions for revision________________________ |
| 1. **Negative Psychological Impact:** After screening, there is approximately a 15% chance that the result will be abnormal, which may cause you to feel anxious. How important is this impact to you? *****   □ 0 (Not important at all) □ 1 □ 2 □ 3 □ 4 □ 5 (Extremely important) |
| □ Appropriate  □ Not appropriate  □ Appropriate after revision |
| Suggestions for revision________________________ |
| 1. **Negative Psychological Impact:** After screening, there is approximately a 13% chance that, despite a normal test result, you actually have cancer. If cancer is discovered later, it may cause psychological distress. How important is this impact to you? *****   □ 0 (Not important at all) □ 1 □ 2 □ 3 □ 4 □ 5 (Extremely important) |
| □ Appropriate  □ Not appropriate  □ Appropriate after revision |
| Suggestions for revision________________________ |
| 1. **Negative Psychological Impact:** After screening, there is approximately a 3% chance that a subsequent biopsy will confirm cancer, and a cancer diagnosis may cause you severe anxiety. How important is this impact to you? *****   □ 0 (Not important at all) □ 1 □ 2 □ 3 □ 4 □ 5 (Extremely important) |
| □ Appropriate  □ Not appropriate  □ Appropriate after revision |
| Suggestions for revision________________________ |
| **Part 4** |
| In the previous step, we assessed the importance of each item (A–J) as determined by participants’ personal values. We now aim to help participants further reflect on the importance of each of these items and ask them to identify the two most important items to facilitate their final decision.  Please evaluate whether this questionnaire format can assist both participants and the research team in identifying which two of the ten items participants consider most important. |
| 1. **Which item do you consider the most important? *** □ A □ B □ C □ D □ E □ F □ G □ H □ I □ J |
| 1. **Which item do you consider the second most important? ***   □ A □ B □ C □ D □ E □ F □ G □ H □ I □ J |
| **Part 5** |
| We also wish to understand the participant’s final decision.  Please evaluate whether this questionnaire format can help both participants and the research team understand the participant’s final decision. |
| **My decision is (choose one) *:** □ I choose to undergo screening □ I choose not to undergo screening □ I am still uncertain and would like more time to decide |
| Suggestions for revision________________________ |
| **Expert Feedback Section** |
| We sincerely invite your comments and suggestions regarding the overall questionnaire.  _____________________________________________________________________  _____________________________________________________________________  _____________________________________________________________________  _____________________________________________________________________ |
|  |
