# Supplementary material for: Web-Based Personalized Machine Learning Recommendations to Enhance Shared Decision-Making in Prostate-Specific Antigen Screening: Randomized Controlled Trial
Source: JMIR Aging. 2026 Apr 13;9:e83238. doi: 10.2196/83238 (PMC13075628; doi:10.2196/83238)
Supplement: Multimedia Appendix 5 [file aging-v9-e83238-s005.docx]

**Appendix 5. Knowledge Test Items**

1. Screening Accuracy: "A normal screening result does not necessarily mean there is no cancer, and an abnormal result does not necessarily mean cancer." (Correct: True)

2. Disease Progression: "Prostate cancer mostly grows slowly; some cancers may never cause illness in a lifetime." (Correct: True)

3. Biopsy Limitations: "Even if a prostate biopsy result is normal, there is still a possibility that cancer is present." (Correct: True)
