# Supplementary figures and images for: Web-Based Personalized Machine Learning Recommendations to Enhance Shared Decision-Making in Prostate-Specific Antigen Screening: Randomized Controlled Trial
Source: JMIR Aging. 2026 Apr 13;9:e83238. doi: 10.2196/83238 (PMC13075628; doi:10.2196/83238)

**Appendix 6. web link and interface**

Link: http://psachoice.shinyapps.io/psapsa/


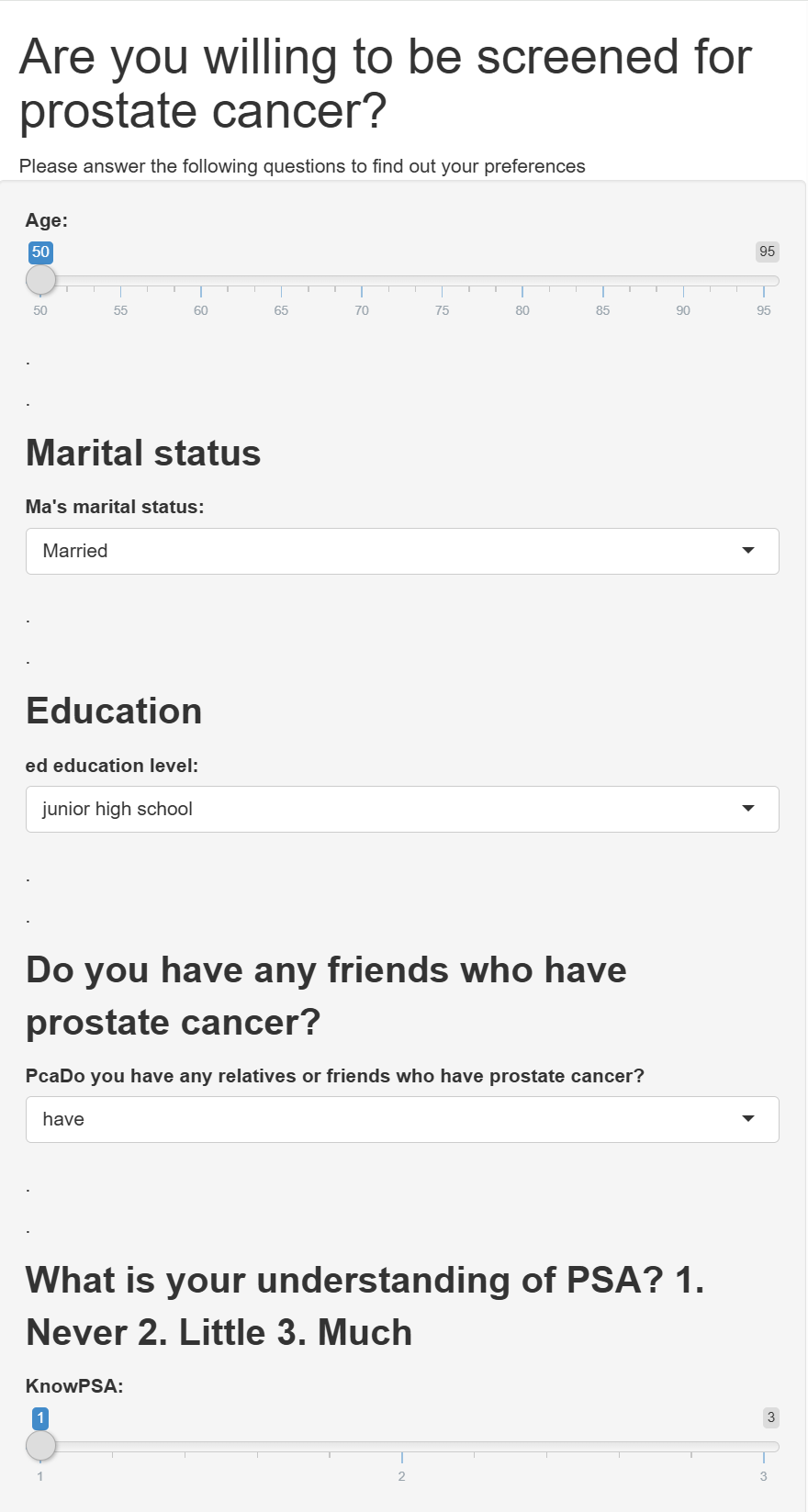


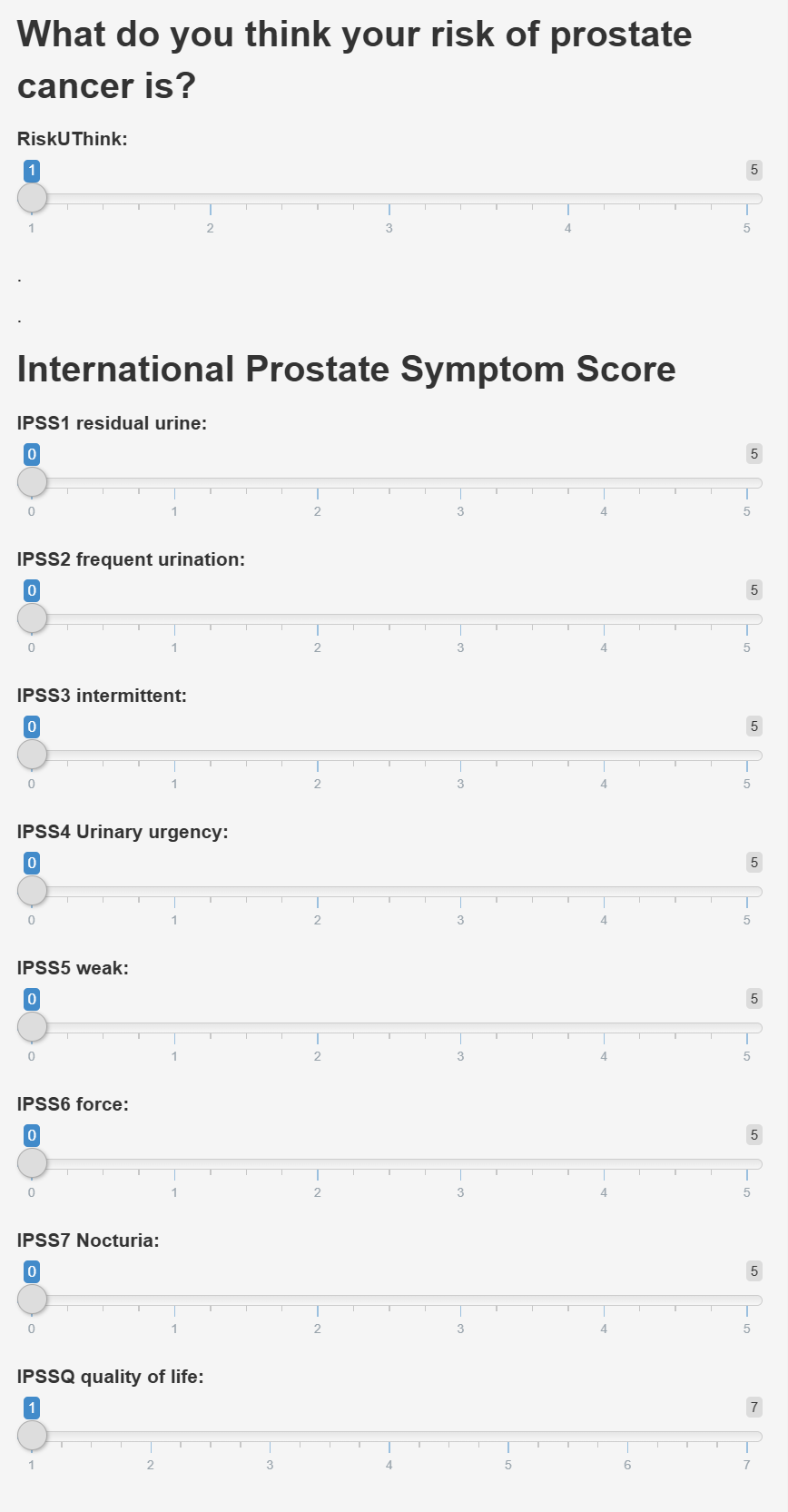


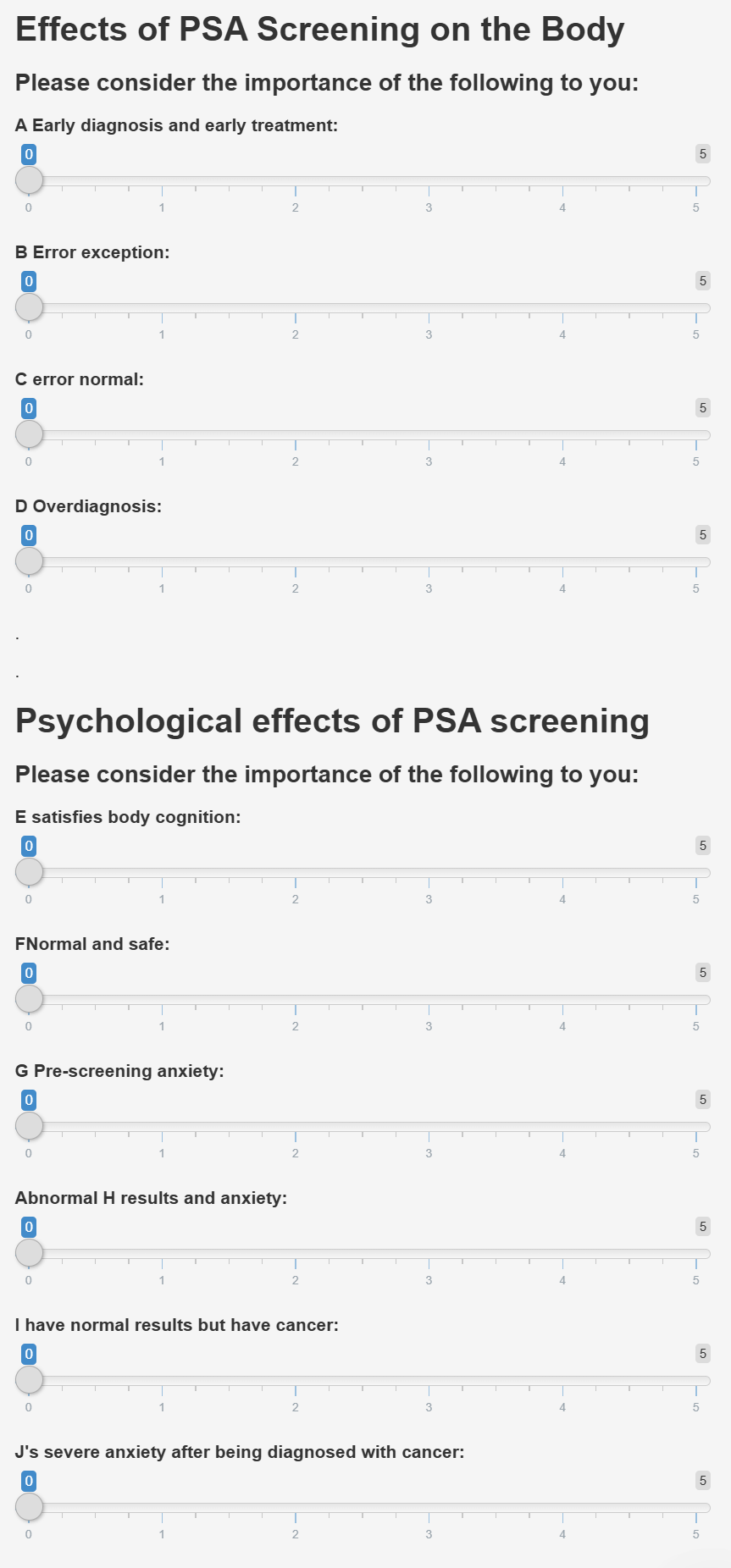


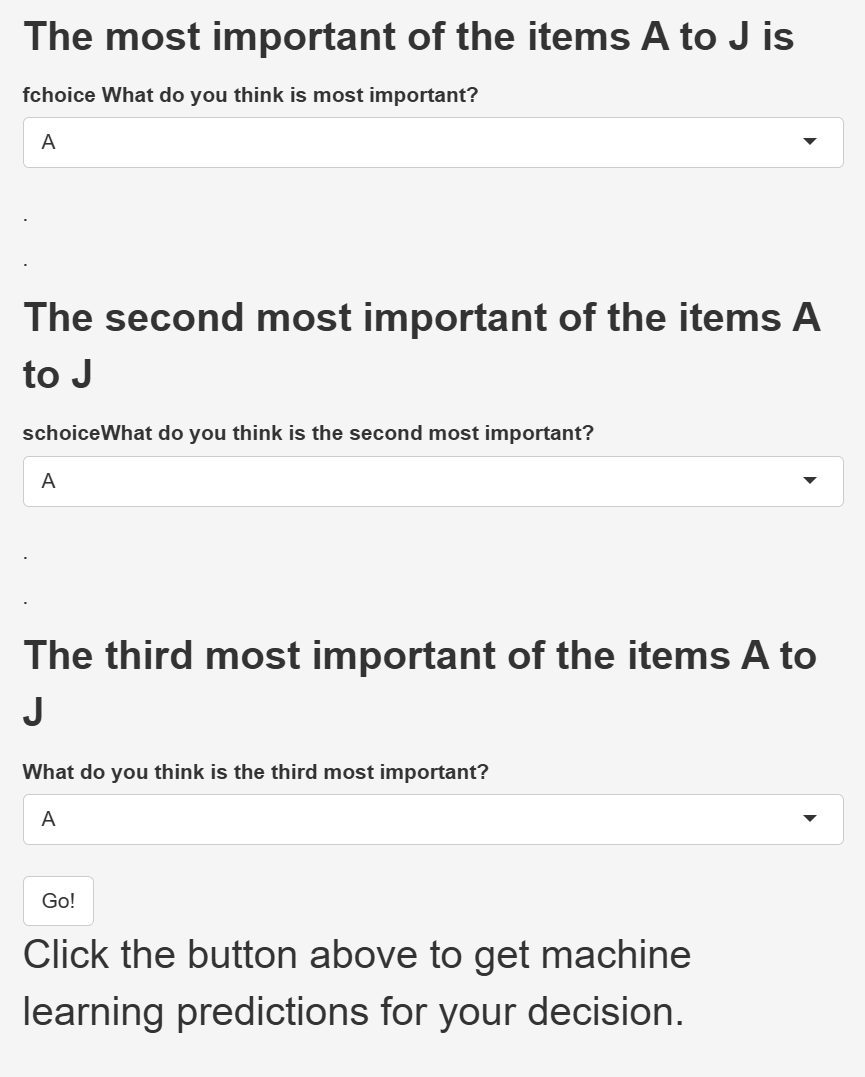

Supplement: Multimedia Appendix 6 [file aging-v9-e83238-s006.docx]
