# Supplementary material for: Web-Based Personalized Machine Learning Recommendations to Enhance Shared Decision-Making in Prostate-Specific Antigen Screening: Randomized Controlled Trial
Source: JMIR Aging. 2026 Apr 13;9:e83238. doi: 10.2196/83238 (PMC13075628; doi:10.2196/83238)
Supplement: Multimedia Appendix 7 [file aging-v9-e83238-s007.docx]

**Appendix 7. Machine Learning Model Development**

1. **Baseline Algorithms and Architecture**
2. **Logistic Regression (LGR)**

LGR is a supervised learning classification algorithm primarily used for binary classification problems[1]. Unlike linear regression, logistic regression employs the sigmoid function to map the output of a linear combination to a probability value between 0 and 1[1, 2]. Given intercept term $\beta_{0}$coefficient $\beta_{i}$ for n features $x_{i}$, it’s formulation is:

$$p=\frac{1}{1+e^{-\left( \beta_{0}+\sum_{i=1}^{n} \beta_{i}x_{i} \right)}}$$

1. **Random Forest (RF)**

RF, introduced by Leo Breiman in 2001, is an ensemble learning algorithm. It is a collection of tree-structured classifiers, in which each classifier (decision tree) contributes to the final prediction [3, 4]. The core mechanism of the algorithm employs the bagging technique. Specifically, multiple subsets are drawn with replacement from the original training dataset, and each subset is used to train an individual decision tree. This approach reduces model variance and increases stability, making Random Forest a robust and widely adopted method for classification and regression tasks[3].

$$\hat{f}=\frac{1}{B}\sum_{b=1}^{B} f_{b}\left( x^{'} \right)$$

where *B* is the total number of decision trees and $f_{b}\left( x^{'} \right)$ represents the prediction made from the *b*-th tree.

1. **Support Vector Machine (SVM)**

SVM, developed by Vapnik et al. in the early 1990s , identifies the optimal hyperplane that maximally separates classes, grounded in statistical learning theory and the principle of structural risk minimization. [5, 6].Furthermore, SVM leverage the principle of structural risk minimization, which seeks to balance model complexity with empirical performance, thereby reducing the risk of overfitting[7]. Using a Gaussian kernel $K\left( x_{i},x \right)$ the decision function is:

$$f\left( x \right)=\text{sign}\left( \sum_{i=1}^{m} \alpha_{i}y_{i}K\left( x_{i},x \right)+b \right)$$

where $\alpha_{i}$ are Lagrange multiplier; $y_{i}$ the class label of support vector $x_{i}$ and *b* the bias function.

1. **XGBoost (XGB)**

XGB is a gradient boosting framework that sequentially adds decision trees to minimize a regularized objective function, balancing prediction accuracy and model generalization[8-10].

For feature vector $x_{i}$ of the $i$-th instance, the model prediction is:

$$\hat{y_{i}}=\sum_{k=1}^{K} f_{k}\left( x_{i} \right),\quad f_{k}\in\mathcal{F}$$

where $K$ is the number of tress, $f_{k}$ the $k$-th decision rule function, and $\mathcal{F}$ the set of all regression trees.

1. **Multilayer Perceptron (MLP)**

MLP is a feedforward artificial neural network composed of an input layer, one or more hidden layers, and an output layer. Each neuron computes a weighted sum of its inputs, adds a bias term, and applies a nonlinear activation function $\phi$ :

$$a_{j}=\phi\left( \sum_{i=1}^{n} w_{ji}x_{i}+b_{j} \right)$$

MLP is a type of feedforward artificial neural network consisting of an input layer, one or more hidden layers, and an output layer. Each neuron computes a weighted sum of its inputs, applies an activation function, and passes the result to the next layer. Where *aj* is the output (activation) of the *j*-th neuron; *wji*: the weight from the *i*-th input to the *j*-th neuron; *xi* are *i*-th input feature; *bj* is the bias of the *j*-th neuron; and *ϕ* is the activation function (e.g., sigmoid, ReLU, tanh, etc.).

1. **Hyperparameter Optimization Strategy**

For this study, we systematically benchmarked five supervised classifiers: LGR, RF, XGB, MLP, and SVM. For each algorithm, a prespecified search space was defined for relevant hyperparameters. Hyperparameters were optimized via a two-stage global search:

First, a design-of-experiments (DOE) sweep was performed, sampling 20 random hyperparameter configurations per model from the defined ranges. Candidates were ranked based on mean out-of-bag area under the ROC curve (OOB AUC) estimated over 60 bootstrap replicates. The top-K configurations were then retained as seeds.Second, these seeds underwent iterative refinement over 10 generations using an adaptive large-neighborhood search inspired by the Ant Lion Optimizer (ALO).

This stage employed gradually decreasing perturbation scales around current elite candidates; each generation was evaluated via mean OOB AUC on a held-out subset of 60 bootstraps for efficiency[11].The final optimal configuration for each classifier was re-evaluated with all 507 bootstrap OOB samples to confirm generalization, and the best-performing hyperparameter set was fixed for subsequent model comparison.

1. **.632 Bootstrapping Evaluation**

To obtain robust, unbiased estimates of generalization performance, we adopted the .632 bootstrap protocol with 507 iterations (equal to the sample size). In each iteration, a bootstrap sample—with replacement—served as the training set, and the out-of-bag (OOB) instances (~36.8% of data not selected) formed the evaluation set. All six tuned classifiers were independently trained on each bootstrap and evaluated on the corresponding OOB set [12, 13]. All candidate models were evaluated using this protocol, generating OOB estimates of accuracy, Cohen’s kappa, sensitivity, specificity, and ROC AUC. The following metrics were computed: accuracy, Cohen’s kappa, sensitivity, specificity, and ROC AUC. For each model, the apparent (resubstitution) error ($\bar{ⅇrr}$) and mean OOB error (${Err}_{oob}$) were computed. Final performance estimates for all models followed the .632 estimator:

$${Error}_{0.632}=0.368*\bar{ⅇrr}+0.632*{Err}_{oob}$$

This approach reduces the bias inherent in estimation with limited data by incorporating both OOB and apparent errors[12]。

1. **Deep Neural Network Benchmark**

In parallel, a deep neural network (DNN) was implemented as a binary classifier benchmark. The DNN comprised two fully connected ReLU-activated hidden layers with 64 and 32 units, respectively. Each layer included batch normalization and a dropout rate of 0.3 to prevent overfitting. The output layer employed a sigmoid activation function. The model was trained using the Adam optimizer (learning rate = 1e-3) and binary cross-entropy loss. To further mitigate overfitting, 20% of the training data was reserved for validation, and early stopping was implemented.

To stabilize ensemble weight estimation for final reporting, all classical pipelines (LGR, RF, SVM, XGB, MLP) underwent stratified 5‑fold and 10‑fold cross‑validation to yield mean AUCs. These mean AUCs—along with the DNN’s test-set AUC—served as fixed weights in the ensemble variant evaluated under an 80/20 train-test protocol. Finally, all models were refit on 80% of the data and evaluated on the remaining 20% holdout. Across all bootstraps, performance distributions were compared using the Kruskal–Wallis test to identify the optimal algorithm.

**Reference**

1. Sperandei, S., *Understanding logistic regression analysis.* Biochemia medica, 2014. **24**(1): p. 12-18.

2. Best, H. and C. Wolf, *Logistic regression.* The SAGE handbook of regression analysis and causal inference. Los Angeles: Sage, 2015: p. 153-171.

3. Breiman, L., *Random forests.* Machine learning, 2001. **45**: p. 5-32.

4. Huang, Y.-C., et al., *A framework to predict second primary lung cancer patients by using ensemble models.* Annals of Operations Research, 2023: p. 1-25.

5. Bottou, L. and C.-J. Lin, *Support vector machine solvers.* Large scale kernel machines, 2007. **3**(1): p. 301-320.

6. Vapnik, V.N. *The support vector method*. in *International conference on artificial neural networks*. 1997. Springer.

7. Yasar, K. *What is a support vector machine (SVM)?* 2024; Available from: <https://www.techtarget.com/whatis/definition/support-vector-machine-SVM>.

8. Tarwidi, D., et al., *An optimized XGBoost-based machine learning method for predicting wave run-up on a sloping beach.* MethodsX, 2023. **10**: p. 102119.

9. Chen, T. and C. Guestrin. *Xgboost: A scalable tree boosting system*. in *Proceedings of the 22nd acm sigkdd international conference on knowledge discovery and data mining*. 2016.

10. Mitchell, R., et al., *Xgboost: Scalable gpu accelerated learning.* arXiv preprint arXiv:1806.11248, 2018.

11. Shijie, Z., et al., *Ant lion optimizer with chaotic investigation mechanism for optimizing SVM parameters.* Journal of Frontiers of Computer Science & Technology, 2016. **10**(5): p. 722.

12. Efron, B. and R. Tibshirani, *Improvements on cross-validation: the 632+ bootstrap method.* Journal of the American Statistical Association, 1997. **92**(438): p. 548-560.

13. Jiang, W. and R. Simon, *A comparison of bootstrap methods and an adjusted bootstrap approach for estimating the prediction error in microarray classification.* Statistics in medicine, 2007. **26**(29): p. 5320-5334.
