# Supplementary material for: Web-Based Personalized Machine Learning Recommendations to Enhance Shared Decision-Making in Prostate-Specific Antigen Screening: Randomized Controlled Trial
Source: JMIR Aging. 2026 Apr 13;9:e83238. doi: 10.2196/83238 (PMC13075628; doi:10.2196/83238)
Supplement: Multimedia Appendix 8 [file aging-v9-e83238-s008.docx]

**Appendix 8. Performance Evaluation of Machine Learning Models**

The optimal hyperparameters of the five machine learning models, as summarized in Table 1, were determined by employing the ALO [1]. After parameter tuning, the XGB model achieved the highest mean accuracy (84.42%), whereas the RF model attained the highest mean AUC value (93.25%). Because the MEG dataset presented a relatively imbalanced classification problem, accuracy alone might not reliably reflect model discrimination ability. Prior studies have shown that AUC is a more appropriate and robust performance metric under such conditions, as it accounts for both sensitivity and specificity independent of class distribution (Ling, Huang, & Zhang, 2003). Therefore, the RF model—demonstrating superior average AUC performance—was selected as the optimal algorithm. This model served as the foundation for constructing the user interface to be utilized in the subsequent RCT.

As summarized in Table 2, the optimal hyperparameters for the five machine learning models were identified using the ALO [1]. After parameter tuning, the XGB model achieved the highest mean accuracy (84.42%), whereas the RF model attained the highest mean AUC (93.25%). The superior AUC performance of the Random Forest model suggests relatively greater robustness, as AUC is widely regarded as a more reliable metric for model evaluation than accuracy, providing a more informative assessment of classification performance[2, 3]. Given these findings, the RF model—demonstrating the highest overall AUC—was selected as the optimal algorithm in this study. This model subsequently served as the foundation for constructing the user interface, which was later evaluated in the RCT.

Appendix8 Table 1. Algorithmic Design of a Decision-Support Classifier

| **Algorithm** | **AUC** | **95% CI** | **Accuracy** | **Kappa** | **Sensitivity** | **Specificity** |
| --- | --- | --- | --- | --- | --- | --- |
| **LGR** | 0.8524 | 0.809 – 0.896 | 0.8087 | 0.5552 | 0.6154 | 0.8611 |
| **RF** | **0.9325** | **0.902 – 0.963** | 0.7903 | 0.5219 | 0.6857 | 0.9708 |
| **SVM** | 0.9119 | 0.877 – 0.947 | 0.8378 | 0.5026 | 0.6500 | 0.9724 |
| **MLP** | 0.8918 | 0.854 – 0.930 | 0.8068 | 0.5085 | 0.7143 | 0.8358 |
| **XGB** | 0.9186 | 0.885 – 0.952 | 0.8442 | 0.5033 | 0.6227 | 0.9355 |
| **DNN** | 0.8801 | 0.840 – 0.920 | 0.8439 | 0.5404 | 0.6316 | 0.9037 |
| **Abbreviations**: LGR, Logistic Regression; RF, Random Forest; SVM, Support Vector Machine; MLP, Multilayer Perceptron; XGB, XGBoost; DNN, Deep Neural Network; MLSG, Machine Learning Suggestion Group; CG, Control Group. | | | | | | |

**Reference**

1. Shijie, Z., et al., *Ant lion optimizer with chaotic investigation mechanism for optimizing SVM parameters.* Journal of Frontiers of Computer Science & Technology, 2016. **10**(5): p. 722.

2. Li, J., *Area under the ROC Curve has the most consistent evaluation for binary classification.* PloS one, 2024. **19**(12): p. e0316019.

3. Omar, E.D., et al., *Comparative analysis of logistic regression, gradient boosted trees, SVM, and random forest algorithms for prediction of acute kidney injury requiring dialysis after cardiac surgery.* International Journal of Nephrology and Renovascular Disease, 2024: p. 197-204.
