# Supplementary material for: Web-Based Personalized Machine Learning Recommendations to Enhance Shared Decision-Making in Prostate-Specific Antigen Screening: Randomized Controlled Trial
Source: JMIR Aging. 2026 Apr 13;9:e83238. doi: 10.2196/83238 (PMC13075628; doi:10.2196/83238)
Supplement: Multimedia Appendix 9 [file aging-v9-e83238-s009.docx]

**Appendix 9. Variables Description**

|  | **Variables** | **Description (Explanation)** | **Categories / Scale** |
| --- | --- | --- | --- |
| 1 | KnowPSA | Self-reported knowledge level regarding PSA screening. | 1: No knowledge, 2: Limited knowledge, 3: Extensive knowledge |
| 2 | RiskPerception | Perceived personal risk of developing prostate cancer. | 1: Very low, 2: Low, 3: Average, 4: High, 5: Very high |
| 3 | Marriage | Current marital status. | S: Single, M: Married, D: Divorced, W: Widowed |
| 4 | Education | Highest level of education attained. | J: ≤ Junior high school, S: Senior high school, U: ≥ College/University |
| 5 | PCaFH | History of prostate cancer among family or close friends. | Y: Yes, N: No |
| 6 | IPSS_QoL | Quality-of-life (QoL) score from the International Prostate Symptom Score (IPSS). | |
| 7 | PrimaryConcern | The most important factor influencing the screening decision (from IPPI items A–J). | Item (A–J) |
| 8 | SecondaryConcern | The second most important factor influencing the screening decision. |  |
| 9 | TertiaryConcern | The third most important factor influencing the screening decision. |  |
| **IPPI** | | | |
| A | Physiological impact | Potential for PSA screening to increase life expectancy. | |
| B |  | Risk of false-positive results leading to unnecessary biopsies and associated side effects. | |
| C |  | Risk of false-negative results leading to delayed diagnosis and reduced survival time. | |
| D |  | isk of overdiagnosis (detecting clinically insignificant cancer) leading to unnecessary treatment. | |
| E | Psychological impact | Satisfaction from understanding one's own health status through PSA testing | |
| F |  | Sense of relief following a normal PSA test result. | |
| G |  | Anxiety or nervousness experienced before undergoing a PSA blood test. | |
| H |  | Anxiety or distress upon receiving an abnormal PSA test result. | |
| I |  | Psychological distress associated with the possibility of a false-negative result. | |
| J |  | Intense anxiety following a biopsy result that confirms prostate cancer. | |
