# Supplementary material for: Web-Based Personalized Machine Learning Recommendations to Enhance Shared Decision-Making in Prostate-Specific Antigen Screening: Randomized Controlled Trial
Source: JMIR Aging. 2026 Apr 13;9:e83238. doi: 10.2196/83238 (PMC13075628; doi:10.2196/83238)
Supplement: Multimedia Appendix 10 [file aging-v9-e83238-s010.docx]

**Appendix10**.

Appendix10 table1 Detail Baseline characteristics in the MEG group

| **Variables** | | **MEG**^a^ **group**  **(N=507)** | | | |
| --- | --- | --- | --- | --- | --- |
|  |  | **All**  **(N=507)** | **Accept**  **(N=130)** | **Not Now (N=377)** | **P-value** |
| Age (y/o) | | 62.89 ± 9.70 | 63.63 ± 9.41 | 62.63 ± 9.80 | .31 |
| KnowPSA^b^ | | 2.11 ± 0.73 | 2.23 ± 0.81 | 2.07 ± 0.69 | .05 |
| RiskPerception | | 2.50 ± 1.05 | 2.61 ± 1.12 | 2.47 ± 1.02 | .19 |
| Marriage | Married | 447 (88.16) | 110 (64.62) | 337 (89.39) | <.001 |
|  | Divorce | 5 (0.98) | 5 (3.85) | 0 (0) |  |
|  | Single | 18 (3.55) | 9 (6.92) | 9 (2.39) |  |
|  | Widow | 37 (7.30) | 6 (4.62) | 31 (8.22) |  |
| Education | J | 256 (50.49) | 61 (46.92) | 195 (51.72) | .07 |
|  | S | 149 (29.39) | 34 (26.15) | 115 (30.50) |  |
|  | U | 102 (20.12) | 35 (26.92) | 67 (17.77) |  |
| PCaFH^c^ | Yes | 404 (79.68) | 96 (73.85) | 308 (81.70) | .05 |
|  | No | 103 (20.32) | 34 (26.15) | 69 (18.30) |  |
| IPSS^d^ | IPSS 1 | 0.86 ± 1.21 | 1.31 ± 1.48 | 0.70 ± 1.06 | <.001 |
|  | IPSS 2 | 0.89 ± 1.21 | 1.33 ± 1.42 | 0.74 ± 1.09 | <.001 |
|  | IPSS 3 | 0.78 ± 1.25 | 1.20 ± 1.53 | 0.64 ± 1.10 | .008 |
|  | IPSS 4 | 0.78 ± 1.21 | 1.15 ± 1.46 | 0.66 ± 1.08 | .02 |
|  | IPSS 5 | 0.53 ± 1.02 | 0.87 ± 1.26 | 0.42 ± 0.89 | <.001 |
|  | IPSS 6 | 0.64 ± 1.10 | 0.81 ± 1.28 | 0.58 ± 1.02 | .06 |
|  | IPSS 7 | 1.71 ± 1.16 | 2.02 ± 1.29 | 1.60 ± 1.10 | <.001 |
|  | IPSS Q^3^ | 5.12 ± 1.33 | 4.85 ± 1.45 | 5.21 ± 1.28 | .008 |
| IPPI^e^ | A | 3.39 ± 1.27 | 3.93 ± 1.07 | 3.20 ± 1.29 | <.001 |
|  | B | 3.35 ± 1.35 | 3.60 ± 1.26 | 3.26 ± 1.37 | <.001 |
|  | C | 3.67 ± 1.17 | 3.88 ± 1.15 | 3.60 ± 1.17 | .006 |
|  | D | 3.14 ± 1.54 | 3.42 ± 1.35 | 3.05 ± 1.59 | .001 |
|  | E | 3.60 ± 1.26 | 4.43 ± 0.85 | 3.31 ± 1.26 | <.001 |
|  | F | 3.27 ± 1.48 | 4.16 ± 1.00 | 2.96 ± 1.49 | <.001 |
|  | G | 2.26 ± 1.56 | 2.29 ± 1.37 | 2.25 ± 1.62 | .34 |
|  | H | 2.86 ± 1.57 | 2.94 ± 1.26 | 2.84 ± 1.67 | .004 |
|  | I | 3.53 ± 1.40 | 3.71 ± 1.25 | 3.47 ± 1.44 | .005 |
|  | J | 3.12 ± 1.56 | 3.43 ± 1.29 | 3.01 ± 1.63 | <.001 |
| **Top three ranked decision‑making factors (A–J)** ^f^ | | | | | |
| First | A | 88 (17.35) | 32 (24.62) | 56 (14.85) | .01 |
|  | B | 85 (16.76) | 10 (7.69) | 75 (19.89) | .001 |
|  | C | 64 (12.62) | 20 (15.38) | 44 (11.67) | .27 |
|  | D | 42 (8.28) | 3 (2.31) | 39 (10.34) | .004 |
|  | E | 32 (6.31) | 19 (14.62) | 13 (3.45) | <.001 |
|  | F | 19 (3.74) | 8 (6.15) | 11 (2.92) | .09 |
|  | G | 7 (1.38) | 1 (0.77) | 6 (1.59) | .48 |
|  | H | 37 (7.73) | 8 (6.15) | 29 (7.69) | .56 |
|  | I | 104 (20.51) | 25 (19.23) | 79 (20.95) | .67 |
|  | J | 29 (5.72) | 4 (3.08) | 25 (6.63) | .13 |
| Second | A | 29 (5.72) | 5 (3.85) | 24 (6.37) | .28 |
|  | B | 82 (16.17) | 18 (13.85) | 64 (16.98) | .40 |
|  | C | 87 (17.16) | 18 (13.85) | 69 (18.30) | .24 |
|  | D | 61 (12.03) | 16 (12.31) | 45 (11.94) | .91 |
|  | E | 43 (8.48) | 27 (20.77) | 16 (4.24) | <.001 |
|  | F | 29 (5.72) | 17 (13.08) | 12 (3.18) | <.001 |
|  | G | 8 (1.58) | 0 | 8 (2.12) | .09 |
|  | H | 47 (9.27) | 4 (3.08) | 43 (11.41) | .004 |
|  | I | 76 (14.99) | 17 (13.08) | 59 (15.65) | .47 |
|  | J | 45 (8.88) | 8 (6.15) | 37 (9.81) | .20 |
| Third | A | 30 (5.92) | 10 (7.69) | 20 (5.31) | .31 |
|  | B | 61 (12.03) | 11 (8.46) | 50 (13.26) | .14 |
|  | C | 112 (22.09) | 28 (21.54) | 84 (22.28) | .86 |
|  | D | 59 (11.64) | 13 (10.00) | 46 (12.20) | .49 |
|  | E | 28 (5.52) | 13 (10.00) | 15 (3.98) | .009 |
|  | F | 28 (5.52) | 19 (14.62) | 9 (2.39) | <.001 |
|  | G | 20 (3.94) | 2 (1.54) | 18 (4.77) | .10 |
|  | H | 59 (11.64) | 9 (6.92) | 50 (13.26) | .05 |
|  | I | 45 (8.88) | 16 (12.31) | 29 (7.69) | .110 |
|  | J | 65 (12.82) | 9 (6.92) | 56 (14.85) | .01 |
| ^a^MEG: Model Establishment Group; ^b^KnowPSA: How would you rate your level of knowledge regarding PSA screening?; ^c^PCaFF: History of prostate cancer among family or close friends.; ^d^IPSS: International Prostate Symptom Score; ^e^IPPI: Importance for Physiological and Psychological Impact questionnaire; ^f^Decision factors derived from the Importance for IPPI questionnaire. | | | | | |
