# Supplementary material for: Web-Based Personalized Machine Learning Recommendations to Enhance Shared Decision-Making in Prostate-Specific Antigen Screening: Randomized Controlled Trial
Source: JMIR Aging. 2026 Apr 13;9:e83238. doi: 10.2196/83238 (PMC13075628; doi:10.2196/83238)
Supplement: Multimedia Appendix 11 [file aging-v9-e83238-s011.docx]

**Appendix 11. R packages**

| **Abbreviation** | **Algorithms** | **Package** |
| --- | --- | --- |
| LGR | Logistic regression | ISLR |
| MLP | Multilayer perceptron neural network | mxnet GPU version (Rao & Chaparala, 2011) |
| DNN | Deep learning neural network |  |
| RF | Random forest | RandomForest |
| XGboost | Extreme gradient boosting | xgboost |
| SVM | Support vector machine | e107 |
| ALO | Ant Lion Optimizer | metaheuristicOpt (Shijie et al., 2016) |
| **Others** | | |
| Enabled parallel computing on the CPU to reduce data processing time | | doSNOW |
| Calculating accuracy and AUC | | ROCR |
