# Supplementary material for: Web-Based Personalized Machine Learning Recommendations to Enhance Shared Decision-Making in Prostate-Specific Antigen Screening: Randomized Controlled Trial
Source: JMIR Aging. 2026 Apr 13;9:e83238. doi: 10.2196/83238 (PMC13075628; doi:10.2196/83238)
Supplement: Multimedia Appendix 12 [file aging-v9-e83238-s012.docx]

**Appendix12**.

Appendix12 table1 Detail Baseline characteristics in the RCT group

| **Variables** | | **RCT group**  **(N=367)** | | | |
| --- | --- | --- | --- | --- | --- |
|  |  | **All**  **(N=367)** | **MLSG**  **(N=185)** | **CG**  **(N=182)** | **P-value** |
| Age (y/o) | | 64.34 ± 10.30 | 64.77 ± 10.50 | 63.91 ± 10.11 | .42 |
| KnowPSA^a^ | | 1.84 ± 0.70 | 1.88 ± 0.72 | 1.88 ± 0.68 | .28 |
| RiskPerception | | 2.76 ± 0.78 | 2.78 ± 0.68 | 2.74 ± 0.69 | .65 |
| Marriage | Married | 336 (91.55) | 166 (89.73) | 170 (93.41) | .64 |
|  | Divorce | 13 (3.54) | 8 (4.32) | 5 (2.75) |  |
|  | Single | 15 (4.10) | 9 (4.86) | 6 (3.30) |  |
|  | Widow | 3 (0.82) | 2 (1.08) | 1 (0.55) |  |
| Education | J | 211 (57.49) | 109 (58.92) | 102 (56.04) | .19 |
|  | S | 95 (25.89) | 41 (22.16) | 54 (29.67) |  |
|  | U | 61 (16.62) | 35 (18.92) | 26 (14.29) |  |
| PCaFH^b^ | Yes | 45 (12.26) | 25 (13.51) | 20 (10.99) | .46 |
|  | No | 322 (87.74) | 160 (86.49) | 162 (89.01) |  |
| IPSS^c^ | IPSS 1 | 0.76 ± 1.16 | 0.86 ± 1.28 | 0.65 ± 1.02 | .08 |
|  | IPSS 2 | 0.90 ± 1.32 | 0.95 ± 1.38 | 0.86 ± 1.26 | .54 |
|  | IPSS 3 | 0.78 ± 1.11 | 0.93 ± 1.28 | 0.63 ± 0.88 | .008 |
|  | IPSS 4 | 0.76 ± 1.13 | 0.93 ± 1.33 | 0.58 ± 0.85 | .003 |
|  | IPSS 5 | 0.43 ± 0.79 | 0.42 ± 0.80 | 0.44 ± 0.78 | .82 |
|  | IPSS 6 | 0.56 ± 1.02 | 0.65 ± 1.16 | 0.47 ± 0.85 | .09 |
|  | IPSS 7 | 1.73 ± 1.18 | 1.65 ± 1.180 | 1.81 ± 1.17 | .19 |
|  | IPSS Q^3^ | 5.52 ± 1.30 | 5.51 ± 1.33 | 5.54 ± 1.27 | .82 |
| IPPI^d^ | A | 3.15 ± 1.35 | 3.26 ± 1.47 | 3.04 ± 1.22 | .12 |
|  | B | 2.87 ± 1.36 | 2.81 ± 1.50 | 2.95 ± 1.21 | .32 |
|  | C | 2.95 ± 1.30 | 3.00 ± 1.46 | 2.90 ± 1.12 | .46 |
|  | D | 2.95 ± 1.27 | 2.97 ± 1.50 | 2.92 ± 0.98 | .73 |
|  | E | 3.46 ± 1.27 | 3.49 ± 1.34 | 3.43 ± 1.19 | .63 |
|  | F | 3.49 ± 1.30 | 3.49 ± 1.35 | 3.49 ± 1.25 | .95 |
|  | G | 1.79 ± 1.87 | 1.78 ± 1.89 | 1.80 ± 1.86 | .92 |
|  | H | 2.10 ± 1.85 | 2.28 ± 1.93 | 1.92 ± 1.76 | .06 |
|  | I | 2.72 ± 1.55 | 2.86 ± 1.64 | 2.58 ± 1.45 | .08 |
|  | J | 2.84 ± 1.61 | 2.79 ± 1.75 | 2.89 ± 1.47 | .54 |
| **Top three ranked decision‑making factors (A–J)** ^e^ | | | | | |
| First | A | 114 (31.06) | 46 (25.27) | 68 (36.76) | .01 |
|  | B | 48 (13.08) | 30 (16.48) | 18 (9.73) | .05 |
|  | C | 32 (8.72) | 15 (8.24) | 17 (9.19) | .74 |
|  | D | 22 (5.99) | 14 (7.69) | 8 (4.32) | .17 |
|  | E | 65 (17.71) | 37 (20.33) | 28 (15.14) | .19 |
|  | F | 35 (9.54) | 20 (10.99) | 15 (8.11) | .34 |
|  | G | 11 (3.00) | 2 (1.10) | 9 (4.86) | .03 |
|  | H | 13 (3.54) | 7 (3.85) | 6 (3.24) | .75 |
|  | I | 13 (3.54) | 6 (3.30) | 7 (3.78) | .80 |
|  | J | 14 (3.81) | 5 (2.75) | 9 (4.86) | .28 |
| Second | A | 29 (7.90) | 14 (7.69) | 15 (8.11) | .88 |
|  | B | 46 (12.53) | 28 (15.38) | 18 (9.73) | .10 |
|  | C | 61 (16.62) | 32 (17.58) | 29 (15.68) | .62 |
|  | D | 41 (11.17) | 24 (13.19) | 17 (9.19) | .22 |
|  | E | 73 (19.89) | 27 (14.84) | 46 (24.86) | .01 |
|  | F | 52 (14.17) | 22 (12.09) | 30 (16.22) | .25 |
|  | G | 4 (1.09) | 2 (1.10) | 2 (1.08) | .98 |
|  | H | 17 (4.63) | 7 (3.85) | 10 (5.41) | .47 |
|  | I | 19 (5.18) | 12 (6.59) | 7 (3.78) | .22 |
|  | J | 25 (6.81) | 14 (7.69) | 11 (5.95) | .50 |
| Third | A | 36 (9.81) | 19 (10.44) | 17 (9.19) | .68 |
|  | B | 22 (5.99) | 8 (4.40) | 14 (7.57) | .20 |
|  | C | 36 (9.81) | 20 (10.99) | 16 (8.65) | .45 |
|  | D | 57 (15.53) | 34 (18.68) | 23 (12.43) | .09 |
|  | E | 55 (14.99) | 32 (17.58) | 23 (12.43) | .16 |
|  | F | 84 (22.89) | 37 (20.33) | 47 (25.41) | .24 |
|  | G | 13 (3.54) | 3 (1.65) | 10 (5.41) | .05 |
|  | H | 14 (3.81) | 6 (3.30) | 8 (4.32) | .60 |
|  | I | 21 (5.72) | 8 (4.40) | 13 (7.03) | .27 |
|  | J | 29 (7.90) | 15 (8.24) | 14 (7.57) | .81 |
| ^a^KnowPSA: How would you rate your level of knowledge regarding PSA screening?; ^b^PCaFF: History of prostate cancer among family or close friends.; ^c^IPSS: International Prostate Symptom Score; ^d^IPPI: Importance for Physiological and Psychological Impact questionnaire; ^e^Decision factors derived from the Importance for IPPI questionnaire. | | | | | |
