# Supplementary material for: Web-Based Personalized Machine Learning Recommendations to Enhance Shared Decision-Making in Prostate-Specific Antigen Screening: Randomized Controlled Trial
Source: JMIR Aging. 2026 Apr 13;9:e83238. doi: 10.2196/83238 (PMC13075628; doi:10.2196/83238)
Supplement: Checklist 1 [file aging-v9-e83238-s013.pdf]

CONSORT-EHEALTH (V 1.6.1) - Submission/Publication Form

The CONSORT-EHEALTH checklist is intended for authors of randomized trials evaluating web-based and Internet-based applications/interventions, including mobile interventions, electronic games (incl multiplayer games), social media, certain telehealth applications, and other interactive and/or networked electronic applications. Some of the items (e.g. all subitems under item 5 - description of the intervention) may also be applicable for other study designs.

The goal of the CONSORT EHEALTH checklist and guideline is to be
a) a guide for reporting for authors of RCTs,
b) to form a basis for appraisal of an ehealth trial (in terms of validity)

CONSORT-EHEALTH items/subitems are MANDATORY reporting items for studies published in the Journal of Medical Internet Research and other journals / scientific societies endorsing the checklist.

Items numbered 1., 2., 3., 4a., 4b etc are original CONSORT or CONSORT-NPT (non-pharmacologic treatment) items.
Items with Roman numerals (i., ii, iii, iv etc.) are CONSORT-EHEALTH extensions/clarifications.

As the CONSORT-EHEALTH checklist is still considered in a formative stage, we would ask that you also RATE ON A SCALE OF 1-5 how important/useful you feel each item is FOR THE PURPOSE OF THE CHECKLIST and reporting guideline (optional).

Mandatory reporting items are marked with a red *.
In the textboxes, either copy & paste the relevant sections from your manuscript into this form - please include any quotes from your manuscript in QUOTATION MARKS,
or answer directly by providing additional information not in the manuscript, or elaborating on why the item was not relevant for this study.

YOUR ANSWERS WILL BE PUBLISHED AS A SUPPLEMENTARY FILE TO YOUR PUBLICATION IN JMIR AND ARE CONSIDERED PART OF YOUR PUBLICATION (IF ACCEPTED).
Please fill in these questions diligently. Information will not be copyedited, so please use proper spelling and grammar, use correct capitalization, and avoid abbreviations.

DO NOT FORGET TO SAVE AS PDF _AND_ CLICK THE SUBMIT BUTTON SO YOUR ANSWERS ARE IN OUR DATABASE !!!

Citation Suggestion (if you append the pdf as Appendix we suggest to cite this paper in the caption):
Eysenbach G, CONSORT-EHEALTH Group
CONSORT-EHEALTH: Improving and Standardizing Evaluation Reports of Web-based and Mobile Health Interventions
J Med Internet Res 2011;13(4):e126
URL: http://www.jmir.org/2011/4/e126/
doi: 10.2196/jmir.1923
PMID: 22209829

hivicky92@gmail.com 切換帳戶 已儲存草稿
未共用的項目

* 表示必填問題

Your name *

First Last

Yen-Chun Huang

Primary Affiliation (short), City, Country *

University of Toronto, Toronto, Canada

Department of Artificial Intelligence, Tamkang

Your e-mail address *

abc@gmail.com

hivicky92@gmail.com

Title of your manuscript *

Provide the (draft) title of your manuscript.

Web-Based Personalized Machine Learning Recommendations to Enhance Shared Decision-Making in Prostate-Specific Antigen Screening: A Randomized Controlled Trial

Name of your App/Software/Intervention *

If there is a short and a long/alternate name, write the short name first and add the long name in brackets.

PSA SDM Tool (PSA Shared Decision-Making T

Evaluated Version (if any)

e.g. "V1", "Release 2017-03-01", "Version 2.0.27913"

您的回答

Language(s) *

What language is the intervention/app in? If multiple languages are available, separate by comma (e.g. "English, French")

Chinese

URL of your Intervention Website or App

e.g. a direct link to the mobile app on app in appstore (itunes, Google Play), or URL of the website. If the intervention is a DVD or hardware, you can also link to an Amazon page.

http://psachoice.shinyapps.io/psapsa/

URL of an image/screenshot (optional)

您的回答

Accessibility *

Can an enduser access the intervention presently?

- (selected) access is free and open
- access only for special usergroups, not open
- access is open to everyone, but requires payment/subscription/in-app purchases
- app/intervention no longer accessible
- 其他：

Primary Medical Indication/Disease/Condition *

e.g. "Stress", "Diabetes", or define the target group in brackets after the condition, e.g. "Autism (Parents of children with)", "Alzheimers (Informal Caregivers of)"

"male", "Prostate-Specific Antigen Screening"

Primary Outcomes measured in trial *

comma-separated list of primary outcomes reported in the trial

"DCS"

Secondary/other outcomes

Are there any other outcomes the intervention is expected to affect?

您的回答

Recommended "Dose" *

What do the instructions for users say on how often the app should be used?

- ○ Approximately Daily
- ○ Approximately Weekly
- ○ Approximately Monthly
- ○ Approximately Yearly
- ◉ "as needed"
- ○ 其他：

Approx. Percentage of Users (starters) still using the app as recommended after 3 months *

- ○ unknown / not evaluated
- ○ 0-10%
- ○ 11-20%
- ○ 21-30%
- ◉ 31-40%
- ○ 41-50%
- ○ 51-60%
- ○ 61-70%
- ○ 71%-80%
- ○ 81-90%
- ○ 91-100%
- ○ 其他：

Overall, was the app/intervention effective? *

- ( ) yes: all primary outcomes were significantly better in intervention group vs control
- (•) partly: SOME primary outcomes were significantly better in intervention group vs control
- ( ) no statistically significant difference between control and intervention
- ( ) potentially harmful: control was significantly better than intervention in one or more outcomes
- ( ) inconclusive: more research is needed
- ( ) 其他：

Article Preparation Status/Stage *

At which stage in your article preparation are you currently (at the time you fill in this form)

- ( ) not submitted yet - in early draft status
- ( ) not submitted yet - in late draft status, just before submission
- ( ) submitted to a journal but not reviewed yet
- (•) submitted to a journal and after receiving initial reviewer comments
- ( ) submitted to a journal and accepted, but not published yet
- ( ) published
- ( ) 其他：

Journal *

If you already know where you will submit this paper (or if it is already submitted), please provide the journal name (if it is not JMIR, provide the journal name under "other")

- ○ not submitted yet / unclear where I will submit this
- ○ Journal of Medical Internet Research (JMIR)
- ○ JMIR mHealth and UHealth
- ○ JMIR Serious Games
- ○ JMIR Mental Health
- ○ JMIR Public Health
- ○ JMIR Formative Research
- ○ Other JMIR sister journal
- ◉ 其他： jmir aging

Is this a full powered effectiveness trial or a pilot/feasibility trial? *

- ◉ Pilot/feasibility
- ○ Fully powered

Manuscript tracking number *

If this is a JMIR submission, please provide the manuscript tracking number under "other" (The ms tracking number can be found in the submission acknowledgement email, or when you login as author in JMIR. If the paper is already published in JMIR, then the ms tracking number is the four-digit number at the end of the DOI, to be found at the bottom of each published article in JMIR)

- ○ no ms number (yet) / not (yet) submitted to / published in JMIR
- ◉ 其他： #83238

TITLE AND ABSTRACT

1a) TITLE: Identification as a randomized trial in the title

1a) Does your paper address CONSORT item 1a? *

I.e does the title contain the phrase "Randomized Controlled Trial"? (if not, explain the reason under "other")

- (x) yes
- ( ) 其他：

1a-i) Identify the mode of delivery in the title

Identify the mode of delivery. Preferably use "web-based" and/or "mobile" and/or "electronic game" in the title. Avoid ambiguous terms like "online", "virtual", "interactive". Use "Internet-based" only if Intervention includes non-web-based Internet components (e.g. email), use "computer-based" or "electronic" only if offline products are used. Use "virtual" only in the context of "virtual reality" (3-D worlds). Use "online" only in the context of "online support groups". Complement or substitute product names with broader terms for the class of products (such as "mobile" or "smart phone" instead of "iphone"), especially if the application runs on different platforms.

	1	2	3	4	5	
subitem not at all important	( )	( )	( )	( )	(x)	essential

清除選取的項目

Does your paper address subitem 1a-i? *

Copy and paste relevant sections from manuscript title (include quotes in quotation marks "like this" to indicate direct quotes from your manuscript), or elaborate on this item by providing additional information not in the ms, or briefly explain why the item is not applicable/relevant for your study

Web-Based Personalized Machine Learning Recommendations

1a-ii) Non-web-based components or important co-interventions in title

Mention non-web-based components or important co-interventions in title, if any (e.g., "with telephone support").

	1	2	3	4	5	
subitem not at all important	( )	( )	( )	(x)	( )	essential

清除選取的項目

Does your paper address subitem 1a-ii?

Copy and paste relevant sections from manuscript title (include quotes in quotation marks "like this" to indicate direct quotes from your manuscript), or elaborate on this item by providing additional information not in the ms, or briefly explain why the item is not applicable/relevant for your study

The video-based decision aid (IC-3) and tutor-led consultation (IC-4) represent standard components of evidence-based shared decision making for PSA screening, delivered identically to both the machine learning-supported group (MLSG) and control group (CG). These conventional interventions are integral to both study arms and thus not uniquely characteristic of the machine learning intervention. The manuscript title appropriately emphasizes the novel machine learning-supported decision aid as the primary intervention.

1a-iii) Primary condition or target group in the title

Mention primary condition or target group in the title, if any (e.g., "for children with Type I Diabetes") Example: A Web-based and Mobile Intervention with Telephone Support for Children with Type I Diabetes: Randomized Controlled Trial

	1	2	3	4	5	
subitem not at all important	○	○	○	○	◉	essential

清除選取的項目

Does your paper address subitem 1a-iii? *

Copy and paste relevant sections from manuscript title (include quotes in quotation marks "like this" to indicate direct quotes from your manuscript), or elaborate on this item by providing additional information not in the ms, or briefly explain why the item is not applicable/relevant for your study

YES
Web-Based Personalized Machine Learning Recommendations to Enhance Shared Decision-Making in Prostate-Specific Antigen Screening: A Randomized Controlled Trial

1b) ABSTRACT: Structured summary of trial design, methods, results, and conclusions

NPT extension: Description of experimental treatment, comparator, care providers, centers, and blinding status.

1b-i) Key features/functionalities/components of the intervention and comparator in the METHODS section of the ABSTRACT

Mention key features/functionalities/components of the intervention and comparator in the abstract. If possible, also mention theories and principles used for designing the site. Keep in mind the needs of systematic reviewers and indexers by including important synonyms. (Note: Only report in the abstract what the main paper is reporting. If this information is missing from the main body of text, consider adding it)

	1	2	3	4	5	
subitem not at all important	○	○	○	○	◉	essential

清除選取的項目

Does your paper address subitem 1b-i? *

Copy and paste relevant sections from the manuscript abstract (include quotes in quotation marks "like this" to indicate direct quotes from your manuscript), or elaborate on this item by providing additional information not in the ms, or briefly explain why the item is not applicable/relevant for your study

YES
MLSG included video-based education, medical staff-led comprehension check, knowledge test, values clarification, and ML-generated personalized recommendations ("Accept" vs "Not now"); CG received all components except ML recommendations. The RCT evaluated effects on psychological outcomes (anxiety, satisfaction with information, decisional conflict).

1b-ii) Level of human involvement in the METHODS section of the ABSTRACT

Clarify the level of human involvement in the abstract, e.g., use phrases like “fully automated” vs. “therapist/nurse/care provider/physician-assisted” (mention number and expertise of providers involved, if any). (Note: Only report in the abstract what the main paper is reporting. If this information is missing from the main body of text, consider adding it)

	1	2	3	4	5	
subitem not at all important	○	○	○	○	◉	essential

清除選取的項目

Does your paper address subitem 1b-ii?

Copy and paste relevant sections from the manuscript abstract (include quotes in quotation marks "like this" to indicate direct quotes from your manuscript), or elaborate on this item by providing additional information not in the ms, or briefly explain why the item is not applicable/relevant for your study

YES
MLSG included video-based education, medical staff-led comprehension check, knowledge test, values clarification, and ML-generated personalized recommendations ("Accept" vs "Not now"); CG received all components except ML recommendations. The RCT evaluated effects on psychological outcomes (anxiety, satisfaction with information, decisional conflict).

1b-iii) Open vs. closed, web-based (self-assessment) vs. face-to-face assessments in the METHODS section of the ABSTRACT

Mention how participants were recruited (online vs. offline), e.g., from an open access website or from a clinic or a closed online user group (closed usergroup trial), and clarify if this was a purely web-based trial, or there were face-to-face components (as part of the intervention or for assessment). Clearly say if outcomes were self-assessed through questionnaires (as common in web-based trials). Note: In traditional offline trials, an open trial (open-label trial) is a type of clinical trial in which both the researchers and participants know which treatment is being administered. To avoid confusion, use “blinded” or “unblinded” to indicated the level of blinding instead of “open”, as “open” in web-based trials usually refers to “open access” (i.e. participants can self-enrol). (Note: Only report in the abstract what the main paper is reporting. If this information is missing from the main body of text, consider adding it)

	1	2	3	4	5	
subitem not at all important	○	○	○	◉	○	essential

清除選取的項目

Does your paper address subitem 1b-iii?

Copy and paste relevant sections from the manuscript abstract (include quotes in quotation marks "like this" to indicate direct quotes from your manuscript), or elaborate on this item by providing additional information not in the ms, or briefly explain why the item is not applicable/relevant for your study

YES
The data used in our research were retrieved offline from Taiwan St. Joseph's Hospital between October 2017 and April 2018, for middle-aged and older patients.

1b-iv) RESULTS section in abstract must contain use data

Report number of participants enrolled/assessed in each group, the use/uptake of the intervention (e.g., attrition/adherence metrics, use over time, number of logins etc.), in addition to primary/secondary outcomes. (Note: Only report in the abstract what the main paper is reporting. If this information is missing from the main body of text, consider adding it)

	1	2	3	4	5	
subitem not at all important	○	○	○	○	◉	essential

清除選取的項目

Does your paper address subitem 1b-iv?

Copy and paste relevant sections from the manuscript abstract (include quotes in quotation marks "like this" to indicate direct quotes from your manuscript), or elaborate on this item by providing additional information not in the ms, or briefly explain why the item is not applicable/relevant for your study

YES
The data used in our research were retrieved offline from Taiwan St. Joseph's Hospital between October 2017 and April 2018, for middle-aged and older patients. A total of 507 participants constituted the Model Establishment Group (MEG), training and evaluating six machine-learning algorithms. Subsequently, an additional 367 participants were randomized 1:1 to either the Machine-Learning Suggestion Group (MLSG) or Control Group (CG). MLSG included video-based education, medical staff-led comprehension check, knowledge test, values clarification, and ML-generated personalized recommendations ("Accept" vs "Not now"); CG received all components except ML recommendations. The RCT evaluated effects on psychological outcomes (anxiety, satisfaction with information, decisional conflict).

1b-v) CONCLUSIONS/DISCUSSION in abstract for negative trials

Conclusions/Discussions in abstract for negative trials: Discuss the primary outcome - if the trial is negative (primary outcome not changed), and the intervention was not used, discuss whether negative results are attributable to lack of uptake and discuss reasons. (Note: Only report in the abstract what the main paper is reporting. If this information is missing from the main body of text, consider adding it)

	1	2	3	4	5	
subitem not at all important	○	○	○	◉	○	essential

清除選取的項目

Does your paper address subitem 1b-v?

Copy and paste relevant sections from the manuscript abstract (include quotes in quotation marks "like this" to indicate direct quotes from your manuscript), or elaborate on this item by providing additional information not in the ms, or briefly explain why the item is not applicable/relevant for your study

These findings suggest that ML-based decision aids can complement, rather than replace, clinical consultations by embedding personalized, AI-driven recommendations into SDM workflows tailored to adults, thereby improving informed choice, enhancing decision self-efficacy, and sustaining reductions in decisional conflict over time.

INTRODUCTION

2a) In INTRODUCTION: Scientific background and explanation of rationale

2a-i) Problem and the type of system/solution

Describe the problem and the type of system/solution that is object of the study: intended as stand-alone intervention vs. incorporated in broader health care program? Intended for a particular patient population? Goals of the intervention, e.g., being more cost-effective to other interventions, replace or complement other solutions? (Note: Details about the intervention are provided in “Methods” under 5)

	1	2	3	4	5	
subitem not at all important	○	○	○	◉	○	essential

清除選取的項目

Does your paper address subitem 2a-i? *

Copy and paste relevant sections from the manuscript (include quotes in quotation marks "like this" to indicate direct quotes from your manuscript), or elaborate on this item by providing additional information not in the ms, or briefly explain why the item is not applicable/relevant for your study

Among older adults, SDM is indispensable because health literacy limitations, sensory or cognitive impairment, and multimorbidity can hinder risk comprehension and value clarification, increasing reliance on physician-directed choices[13-16].

To address these gaps, a novel ML-based decision aid was developed for prostate cancer screening and early treatment deliberation in adults, integrating individualized risk profiles with preference-elicitation modules and systematic assessment of anxiety, satisfaction, and decisional conflict.

This ML-based SDM was designed as a complementary component within existing SDM workflows for PSA screening consultations, rather than a stand-alone intervention. Targeted specifically at middle-aged and older adult men facing PSA screening decisions, it aims to augment traditional decision aids by providing individualized risk predictions that align recommendations with patient values and clinical outcomes observed in similar profiles.

2a-ii) Scientific background, rationale: What is known about the (type of) system

Scientific background, rationale: What is known about the (type of) system that is the object of the study (be sure to discuss the use of similar systems for other conditions/diagnoses, if appropiate), motivation for the study, i.e. what are the reasons for and what is the context for this specific study, from which stakeholder viewpoint is the study performed, potential impact of findings [2]. Briefly justify the choice of the comparator.

	1	2	3	4	5	
subitem not at all important	○	○	○	◉	○	essential

清除選取的項目

Does your paper address subitem 2a-ii? *

Copy and paste relevant sections from the manuscript (include quotes in quotation marks "like this" to indicate direct quotes from your manuscript), or elaborate on this item by providing additional information not in the ms, or briefly explain why the item is not applicable/relevant for your study

Findings will inform scalable integration of ML recommendations into routine PSA screening workflows across Asian healthcare systems, potentially reducing overtreatment while empowering informed patient choice.

2b) In INTRODUCTION: Specific objectives or hypotheses

Does your paper address CONSORT subitem 2b? *

Copy and paste relevant sections from the manuscript (include quotes in quotation marks "like this" to indicate direct quotes from your manuscript), or elaborate on this item by providing additional information not in the ms, or briefly explain why the item is not applicable/relevant for your study

"The primary aim of this study is to evaluate whether this ML-integrated strategy enhances decision self-efficacy, reduces decisional conflict and indecision, and lowers decisional regret among middle-aged and older men."

METHODS

3a) Description of trial design (such as parallel, factorial) including allocation ratio

Does your paper address CONSORT subitem 3a? *

Copy and paste relevant sections from the manuscript (include quotes in quotation marks "like this" to indicate direct quotes from your manuscript), or elaborate on this item by providing additional information not in the ms, or briefly explain why the item is not applicable/relevant for your study

"Subsequently, we conducted a parallel-group randomized controlled trial with a 1:1 allocation ratio. An additional 380 participants were recruited and randomly assigned to either the Machine Learning Suggestion Group (MLSG, n=190) or the Control Group (CG, n=190) to evaluate the impact of ML-based decision aid recommendations."

3b) Important changes to methods after trial commencement (such as eligibility criteria), with reasons

Does your paper address CONSORT subitem 3b? *

Copy and paste relevant sections from the manuscript (include quotes in quotation marks "like this" to indicate direct quotes from your manuscript), or elaborate on this item by providing additional information not in the ms, or briefly explain why the item is not applicable/relevant for your study

"The study was conducted in accordance with the original protocol, and there were no changes to the methods (including eligibility criteria and outcomes) after trial commencement. The study protocol was reviewed and approved by the Institutional Review Board (IRB) of Fu Jen Catholic University (C105142), and informed consent was obtained from all participants."

3b-i) Bug fixes, Downtimes, Content Changes

Bug fixes, Downtimes, Content Changes: ehealth systems are often dynamic systems. A description of changes to methods therefore also includes important changes made on the intervention or comparator during the trial (e.g., major bug fixes or changes in the functionality or content) (5-iii) and other “unexpected events” that may have influenced study design such as staff changes, system failures/downtimes, etc. [2].

	1	2	3	4	5	
subitem not at all important	○	○	○	◉	○	essential

清除選取的項目

Does your paper address subitem 3b-i?

Copy and paste relevant sections from the manuscript (include quotes in quotation marks "like this" to indicate direct quotes from your manuscript), or elaborate on this item by providing additional information not in the ms, or briefly explain why the item is not applicable/relevant for your study

2.4 System Stability and Quality Control
"The ML-based decision aid was deployed on a dedicated Shiny server. All intervention components (video content, questionnaires, IPPI interface, and the ML recommendation algorithm) were finalized and locked before randomization. No content modifications, algorithm updates, or bug fixes were implemented after the trial commenced. Study personnel remained consistent throughout the enrollment period, and no unexpected system failures affected data collection."

4a) Eligibility criteria for participants

Does your paper address CONSORT subitem 4a? *

Copy and paste relevant sections from the manuscript (include quotes in quotation marks "like this" to indicate direct quotes from your manuscript), or elaborate on this item by providing additional information not in the ms, or briefly explain why the item is not applicable/relevant for your study

"We recruited men eligible if they: (1) were aged ≥50 years, (2) presented to Taiwan St. Joseph Hospital for routine care between October 2017 and April 2018, and (3) were able to provide informed consent and communicate in Mandarin Chinese. This age threshold aligns with USPSTF and AUA guidelines recommending PSA screening discussions starting at age 50, with shared decision-making particularly emphasized for men aged 55–69 years [32, 33]. Men were excluded if they had: (1) any documented psychiatric disorder that might impair decision-making capacity, (2) prior PSA testing (to ensure treatment-naïve status), or (3) a history of prostate surgery or prostate cancer (to avoid confounding from prior disease knowledge or treatment experience)."

4a-i) Computer / Internet literacy

Computer / Internet literacy is often an implicit “de facto” eligibility criterion - this should be explicitly clarified.

	1	2	3	4	5	
subitem not at all important	○	○	○	◉	○	essential

清除選取的項目

Does your paper address subitem 4a-i?

Copy and paste relevant sections from the manuscript (include quotes in quotation marks "like this" to indicate direct quotes from your manuscript), or elaborate on this item by providing additional information not in the ms, or briefly explain why the item is not applicable/relevant for your study

Notably, computer and internet literacy were not required for participation; all digital intervention components were facilitated by trained research staff to ensure accessibility for older adults regardless of their technological proficiency.

4a-ii) Open vs. closed, web-based vs. face-to-face assessments:

Open vs. closed, web-based vs. face-to-face assessments: Mention how participants were recruited (online vs. offline), e.g., from an open access website or from a clinic, and clarify if this was a purely web-based trial, or there were face-to-face components (as part of the intervention or for assessment), i.e., to what degree got the study team to know the participant. In online-only trials, clarify if participants were quasi-anonymous and whether having multiple identities was possible or whether technical or logistical measures (e.g., cookies, email confirmation, phone calls) were used to detect/prevent these.

	1	2	3	4	5	
subitem not at all important	○	○	○	○	◉	essential

清除選取的項目

Does your paper address subitem 4a-ii? *

Copy and paste relevant sections from the manuscript (include quotes in quotation marks "like this" to indicate direct quotes from your manuscript), or elaborate on this item by providing additional information not in the ms, or briefly explain why the item is not applicable/relevant for your study

"This study employed a closed, face-to-face recruitment strategy at Taiwan St. Joseph Hospital... Participant identity and eligibility were verified against electronic medical records by research staff to prevent multiple entries." "Although the intervention utilized a web-based interface, all assessments and digital components were administered face-to-face by trained research staff. This supervised approach ensured accessibility for older adults... and guaranteed that the study team had direct contact** with all participants."

4a-iii) Information giving during recruitment

Information given during recruitment. Specify how participants were briefed for recruitment and in the informed consent procedures (e.g., publish the informed consent documentation as appendix, see also item X26), as this information may have an effect on user self-selection, user expectation and may also bias results.

	1	2	3	4	5	
subitem not at all important	○	○	○	○	○	essential

Does your paper address subitem 4a-iii?

Copy and paste relevant sections from the manuscript (include quotes in quotation marks "like this" to indicate direct quotes from your manuscript), or elaborate on this item by providing additional information not in the ms, or briefly explain why the item is not applicable/relevant for your study

您的回答

4b) Settings and locations where the data were collected

Does your paper address CONSORT subitem 4b? *

Copy and paste relevant sections from the manuscript (include quotes in quotation marks "like this" to indicate direct quotes from your manuscript), or elaborate on this item by providing additional information not in the ms, or briefly explain why the item is not applicable/relevant for your study

The study protocol was reviewed and approved by the Institutional Review Board (IRB) of Fu Jen Catholic University (C105142), and informed consent was obtained from all participants.

4b-i) Report if outcomes were (self-)assessed through online questionnaires

Clearly report if outcomes were (self-)assessed through online questionnaires (as common in web-based trials) or otherwise.

	1	2	3	4	5	
subitem not at all important	○	○	○	○	○	essential

Does your paper address subitem 4b-i? *

Copy and paste relevant sections from the manuscript (include quotes in quotation marks "like this" to indicate direct quotes from your manuscript), or elaborate on this item by providing additional information not in the ms, or briefly explain why the item is not applicable/relevant for your study

All participants completed psychological measures (IC-8; ~4 min; STAI, SA, and DCS) and made their screening decision at IC-9 (self-paced) via the same web-based interface used for the intervention."

Data collected during IC-1 (baseline demographics and clinical history), IC-2 (IPSS urinary symptom assessment), and IC-6 (IPPI values clarification) were completed within the same web-based interface...

Although the intervention utilized a web-based interface, all assessments and digital components were administered face-to-face by trained research staff. This supervised approach ensured accessibility for older adults... while guaranteeing that the study team had direct contact with all participants

4b-ii) Report how institutional affiliations are displayed

Report how institutional affiliations are displayed to potential participants [on ehealth media], as affiliations with prestigious hospitals or universities may affect volunteer rates, use, and reactions with regards to an intervention.(Not a required item – describe only if this may bias results)

	1	2	3	4	5	
subitem not at all important	○	○	○	◉	○	essential

清除選取的項目

Does your paper address subitem 4b-ii?

Copy and paste relevant sections from the manuscript (include quotes in quotation marks "like this" to indicate direct quotes from your manuscript), or elaborate on this item by providing additional information not in the ms, or briefly explain why the item is not applicable/relevant for your study

您的回答

5) The interventions for each group with sufficient details to allow replication, including how and when they were actually administered

5-i) Mention names, credential, affiliations of the developers, sponsors, and owners

Mention names, credential, affiliations of the developers, sponsors, and owners [6] (if authors/evaluators are owners or developer of the software, this needs to be declared in a “Conflict of interest” section or mentioned elsewhere in the manuscript).

	1	2	3	4	5	
subitem not at all important	○	○	○	○	◉	essential

清除選取的項目

Does your paper address subitem 5-i?

Copy and paste relevant sections from the manuscript (include quotes in quotation marks "like this" to indicate direct quotes from your manuscript), or elaborate on this item by providing additional information not in the ms, or briefly explain why the item is not applicable/relevant for your study

The study employed a closed, face-to-face recruitment strategy at Taiwan St. Joseph Hospital. We recruited men eligible if they... presented for routine care... Participant identity and eligibility were verified against electronic medical records by research staff... The intervention was delivered in a facilitated setting... Medical staff acted as 'digital navigators'...
Although the specific display of institutional affiliations on the web interface is not explicitly detailed in the text, the recruitment and intervention occurred entirely within the hospital setting (Taiwan St. Joseph Hospital) during routine care visits. Therefore, participants were fully aware of the hospital affiliation as they were already patients at the institution receiving facilitated guidance from uniformed medical staff. The web-based decision aid was presented as a hospital-endorsed tool, likely enhancing trust and engagement, but this effect would be consistent across both study arms as they were recruited in the same clinical environment.

5-ii) Describe the history/development process

Describe the history/development process of the application and previous formative evaluations (e.g., focus groups, usability testing), as these will have an impact on adoption/use rates and help with interpreting results.

	1	2	3	4	5	
subitem not at all important	○	○	○	○	○	essential

Does your paper address subitem 5-ii?

Copy and paste relevant sections from the manuscript (include quotes in quotation marks "like this" to indicate direct quotes from your manuscript), or elaborate on this item by providing additional information not in the ms, or briefly explain why the item is not applicable/relevant for your study

您的回答

5-iii) Revisions and updating

Revisions and updating. Clearly mention the date and/or version number of the application/intervention (and comparator, if applicable) evaluated, or describe whether the intervention underwent major changes during the evaluation process, or whether the development and/or content was “frozen” during the trial. Describe dynamic components such as news feeds or changing content which may have an impact on the replicability of the intervention (for unexpected events see item 3b).

	1	2	3	4	5	
subitem not at all important	○	○	○	○	◉	essential

清除選取的項目

Does your paper address subitem 5-iii?

Copy and paste relevant sections from the manuscript (include quotes in quotation marks "like this" to indicate direct quotes from your manuscript), or elaborate on this item by providing additional information not in the ms, or briefly explain why the item is not applicable/relevant for your study

Yes
"Development Framework: Drawing on the frameworks proposed by Eila et al., Rychetnik et al., and Ferrante et al. [26-28], we developed the IPPI questionnaire." [Methods 2.1]Expert Validation:"Underwent expert validity assessment by 10 urologists... Reliability analysis (first 300 patients): Cronbach's α = 0.838 (physiological), 0.900 (psychological)." [Methods 2.1.1]
Iterative ML Training:"Model iteratively retrained after every batch of 20 participants until AUC ≥ 0.80." [Methods 2.1.3]
These describe formative evaluation preceding the RCT.

5-iv) Quality assurance methods

Provide information on quality assurance methods to ensure accuracy and quality of information provided [1], if applicable.

	1	2	3	4	5	
subitem not at all important	○	○	○	○	○	essential

Does your paper address subitem 5-iv?

Copy and paste relevant sections from the manuscript (include quotes in quotation marks "like this" to indicate direct quotes from your manuscript), or elaborate on this item by providing additional information not in the ms, or briefly explain why the item is not applicable/relevant for your study

您的回答

5-v) Ensure replicability by publishing the source code, and/or providing screenshots/screen-capture video, and/or providing flowcharts of the algorithms used

Ensure replicability by publishing the source code, and/or providing screenshots/screen-capture video, and/or providing flowcharts of the algorithms used. Replicability (i.e., other researchers should in principle be able to replicate the study) is a hallmark of scientific reporting.

	1	2	3	4	5	
subitem not at all important	○	○	○	◉	○	essential

清除選取的項目

Does your paper address subitem 5-v?

Copy and paste relevant sections from the manuscript (include quotes in quotation marks "like this" to indicate direct quotes from your manuscript), or elaborate on this item by providing additional information not in the ms, or briefly explain why the item is not applicable/relevant for your study

Yes
"Figure 3 presents representative screenshots of the user interface... full English translations provided in Multimedia Appendix 6."
"Technical specifications... hyperparameter optimization... provided in Multimedia Appendix 7.""R code... provided in Multimedia Appendix 10."Detailed interface screenshots (Figure 3), algorithm specifications (Appendix 7), full questionnaires (Appendices 1–4), and analysis code (Appendix 10) enable complete replication of both model development and trial protocol.

5-vi) Digital preservation

Digital preservation: Provide the URL of the application, but as the intervention is likely to change or disappear over the course of the years; also make sure the intervention is archived (Internet Archive, webcitation.org, and/or publishing the source code or screenshots/videos alongside the article). As pages behind login screens cannot be archived, consider creating demo pages which are accessible without login.

	1	2	3	4	5	
subitem not at all important	○	○	○	◉	○	essential

清除選取的項目

Does your paper address subitem 5-vi?

Copy and paste relevant sections from the manuscript (include quotes in quotation marks "like this" to indicate direct quotes from your manuscript), or elaborate on this item by providing additional information not in the ms, or briefly explain why the item is not applicable/relevant for your study

"To support international readability and replicability, full English translations of all interface text and items are provided in Multimedia Appendix 6."
"Figure 3 presents representative screenshots of the user interface... including the integrated data-entry and values-clarification screens (Figure 3a) and the recommendation output screen (Figure 3b)."
"The ML-based decision aid was deployed on a dedicated Shiny server. All intervention components (video content, questionnaires, IPPI interface, and the ML recommendation algorithm) were finalized and locked before randomization."

5-vii) Access

Access: Describe how participants accessed the application, in what setting/context, if they had to pay (or were paid) or not, whether they had to be a member of specific group. If known, describe how participants obtained "access to the platform and Internet" [1]. To ensure access for editors/reviewers/readers, consider to provide a "backdoor" login account or demo mode for reviewers/readers to explore the application (also important for archiving purposes, see vi).

	1	2	3	4	5	
subitem not at all important	○	○	○	◉	○	essential

清除選取的項目

Does your paper address subitem 5-vii? *

Copy and paste relevant sections from the manuscript (include quotes in quotation marks "like this" to indicate direct quotes from your manuscript), or elaborate on this item by providing additional information not in the ms, or briefly explain why the item is not applicable/relevant for your study

Yes
"Participants were recruited at Taiwan St. Joseph Hospital. We recruited men eligible if they: (1) were aged ≥50 years, (2) presented for routine care between October 2017 and April 2018... Notably, computer and internet literacy were not required; although the intervention utilized a web-based interface, all assessments and digital components were administered face-to-face by trained research staff. This supervised approach ensured accessibility for older adults regardless of technological proficiency... To ensure digital equity and protocol adherence, the intervention was delivered in a facilitated setting... Staff provided standardized, one-on-one technical assistance (e.g., interface navigation, font enlargement) to any participant experiencing difficulty."

5-viii) Mode of delivery, features/functionalities/components of the intervention and comparator, and the theoretical framework

Describe mode of delivery, features/functionalities/components of the intervention and comparator, and the theoretical framework [6] used to design them (instructional strategy [1], behaviour change techniques, persuasive features, etc., see e.g., [7, 8] for terminology). This includes an in-depth description of the content (including where it is coming from and who developed it) [1]," whether [and how] it is tailored to individual circumstances and allows users to track their progress and receive feedback" [6]. This also includes a description of communication delivery channels and – if computer-mediated communication is a component – whether communication was synchronous or asynchronous [6]. It also includes information on presentation strategies [1], including page design principles, average amount of text on pages, presence of hyperlinks to other resources, etc. [1].

	1	2	3	4	5	
subitem not at all important	○	○	○	●	○	essential

清除選取的項目

Does your paper address subitem 5-viii? *

Copy and paste relevant sections from the manuscript (include quotes in quotation marks "like this" to indicate direct quotes from your manuscript), or elaborate on this item by providing additional information not in the ms, or briefly explain why the item is not applicable/relevant for your study

Theoretical Framework & Design Principles:
"Drawing on Eila et al., Rychetnik et al., and Ferrante et al. [26-28], we developed the IPPI questionnaire for values clarification. The video-based decision aid (IC-3) covered prostate physiology, morbidity risks, and benefits vs. overdiagnosis risks." [jmir]
Mode of Delivery & Facilitation:
"Facilitated face-to-face delivery via web interface. Medical staff provided operational 'digital navigation' support for older adults (20-25 min total). No decision influence." [jmir](https://www.jmir.org/ojs/public/journals/1/CONSORT-EHEALTH-v1-6.pdf)
Intervention Components & Tailoring:
"9 standardized components. MLSG received personalized ML recommendation (IC-7) based on IC-1-6 data: 'Agree to receive' vs. 'Reconsider/refuse.' Presented as advisory second opinion." [jmir](https://www.jmir.org/ojs/public/journals/1/CONSORT-EHEALTH-v1-6.pdf)
Presentation Strategies:
"Screenshots in Figure 3 (data entry, values clarification, ML output). Full English translations in Multimedia Appendix 6."

5-ix) Describe use parameters

Describe use parameters (e.g., intended “doses” and optimal timing for use). Clarify what instructions or recommendations were given to the user, e.g., regarding timing, frequency, heaviness of use, if any, or was the intervention used ad libitum.

	1	2	3	4	5	
subitem not at all important	○	○	○	○	○	essential

Does your paper address subitem 5-ix?

Copy and paste relevant sections from the manuscript (include quotes in quotation marks "like this" to indicate direct quotes from your manuscript), or elaborate on this item by providing additional information not in the ms, or briefly explain why the item is not applicable/relevant for your study

您的回答

5-x) Clarify the level of human involvement

Clarify the level of human involvement (care providers or health professionals, also technical assistance) in the e-intervention or as co-intervention (detail number and expertise of professionals involved, if any, as well as “type of assistance offered, the timing and frequency of the support, how it is initiated, and the medium by which the assistance is delivered”. It may be necessary to distinguish between the level of human involvement required for the trial, and the level of human involvement required for a routine application outside of a RCT setting (discuss under item 21 – generalizability).

	1	2	3	4	5	
subitem not at all important	○	○	○	○	○	essential

Does your paper address subitem 5-x?

Copy and paste relevant sections from the manuscript (include quotes in quotation marks "like this" to indicate direct quotes from your manuscript), or elaborate on this item by providing additional information not in the ms, or briefly explain why the item is not applicable/relevant for your study

您的回答

5-xi) Report any prompts/reminders used

Report any prompts/reminders used: Clarify if there were prompts (letters, emails, phone calls, SMS) to use the application, what triggered them, frequency etc. It may be necessary to distinguish between the level of prompts/reminders required for the trial, and the level of prompts/reminders for a routine application outside of a RCT setting (discuss under item 21 – generalizability).

	1	2	3	4	5	
subitem not at all important	○	○	○	◉	○	essential

清除選取的項目

Does your paper address subitem 5-xi? *

Copy and paste relevant sections from the manuscript (include quotes in quotation marks "like this" to indicate direct quotes from your manuscript), or elaborate on this item by providing additional information not in the ms, or briefly explain why the item is not applicable/relevant for your study

Yes
"This study employed a closed, face-to-face recruitment strategy at Taiwan St. Joseph Hospital. We recruited men eligible if they... presented for routine care... The intervention comprised nine standardized components delivered in approximately 20–25 minutes... Participants in the randomized controlled trial (RCT) completed the full sequence (IC-1 to IC-9) [in a single session]."As this was a single-session, face-to-face intervention conducted during a routine clinical visit, no remote prompts or reminders (e.g., emails, SMS, phone calls) were utilized to encourage application usage. The entire intervention—from recruitment to the final decision—occurred within a structured clinical encounter facilitated by research staff. Therefore, retention prompts were not applicable to this study design. Future implementation in routine practice would similarly rely on point-of-care integration rather than external reminders.

5-xii) Describe any co-interventions (incl. training/support)

Describe any co-interventions (incl. training/support): Clearly state any interventions that are provided in addition to the targeted eHealth intervention, as ehealth intervention may not be designed as stand-alone intervention. This includes training sessions and support [1]. It may be necessary to distinguish between the level of training required for the trial, and the level of training for a routine application outside of a RCT setting (discuss under item 21 – generalizability.

	1	2	3	4	5	
subitem not at all important	○	○	○	◉	○	essential

清除選取的項目

Does your paper address subitem 5-xii? *

Copy and paste relevant sections from the manuscript (include quotes in quotation marks "like this" to indicate direct quotes from your manuscript), or elaborate on this item by providing additional information not in the ms, or briefly explain why the item is not applicable/relevant for your study

Yes, the manuscript details the co-interventions and support provided to ensure the effective delivery of the eHealth tool, particularly given the older target population.
"Prior to study commencement, all participating medical staff completed a standardized 10-minute training session to ensure proficiency in navigating the DA... and adherence to a neutral facilitation protocol." (Methods, Section 2.1.3)
"To ensure digital equity... the intervention was delivered in a facilitated setting. Medical staff acted as 'digital navigators,' providing standardized, one-on-one technical assistance (e.g., interface navigation) to any participant experiencing difficulty. This support was strictly operational to prevent influencing the decision itself." (Methods, Section 2.1.4)
"At IC-4 (~5 min), trained medical personnel conducted individual counseling to ensure comprehension... using neutral, non-directive language and the teach-back method." (Methods, Section 2.1.4)
These sections clarify that the ML-based decision aid was integrated into a broader, supportive SDM workflow rather than deployed as a standalone tool.

6a) Completely defined pre-specified primary and secondary outcome measures, including how and when they were assessed

Does your paper address CONSORT subitem 6a? *

Copy and paste relevant sections from the manuscript (include quotes in quotation marks "like this" to indicate direct quotes from your manuscript), or elaborate on this item by providing additional information not in the ms, or briefly explain why the item is not applicable/relevant for your study

"Primary Outcome
To address multiplicity, we prioritized the Total Decisional Conflict Scale (DCS) score as the single primary endpoint. The DCS measures participants' uncertainty in making a health-related decision and the factors contributing to that uncertainty. It consists of 16 items scored on a 5-point Likert scale (0=strongly agree to 4=strongly disagree). Total scores range from 0 to 100, with lower scores indicating lower decisional conflict and higher decision quality. The DCS was administered at IC-8, immediately following the intervention and prior to the final decision implementation.""Secondary Outcomes
Secondary outcomes included state anxiety, decision satisfaction, and the final screening decision.

6a-i) Online questionnaires: describe if they were validated for online use and apply CHERRIES items to describe how the questionnaires were designed/deployed

If outcomes were obtained through online questionnaires, describe if they were validated for online use and apply CHERRIES items to describe how the questionnaires were designed/deployed [9].

	1	2	3	4	5	
subitem not at all important	○	○	○	○	○	essential

Does your paper address subitem 6a-i?

Copy and paste relevant sections from manuscript text

您的回答

6a-ii) Describe whether and how “use” (including intensity of use/dosage) was defined/measured/monitored

Describe whether and how “use” (including intensity of use/dosage) was defined/measured/monitored (logins, logfile analysis, etc.). Use/adoption metrics are important process outcomes that should be reported in any ehealth trial.

	1	2	3	4	5	
subitem not at all important	○	○	○	○	○	essential

Does your paper address subitem 6a-ii?

Copy and paste relevant sections from manuscript text

您的回答

6a-iii) Describe whether, how, and when qualitative feedback from participants was obtained

Describe whether, how, and when qualitative feedback from participants was obtained (e.g., through emails, feedback forms, interviews, focus groups).

	1	2	3	4	5	
subitem not at all important	○	○	○	○	○	essential

Does your paper address subitem 6a-iii?

Copy and paste relevant sections from manuscript text

您的回答

6b) Any changes to trial outcomes after the trial commenced, with reasons

Does your paper address CONSORT subitem 6b? *

Copy and paste relevant sections from the manuscript (include quotes in quotation marks "like this" to indicate direct quotes from your manuscript), or elaborate on this item by providing additional information not in the ms, or briefly explain why the item is not applicable/relevant for your study

Yes, our paper addresses CONSORT subitem 6b. There were no changes to trial outcomes after the trial commenced. We explicitly state this in the Methods section under 2.1.3 Study group design framework and 2.4 System Stability and Quality Control:

"The study was conducted in accordance with the original protocol, and there were no changes to the methods (including eligibility criteria and outcomes) after trial commencement."

"All intervention components (video content, questionnaires, IPPI interface, and the ML recommendation algorithm) were finalized and locked before randomization. No content modifications, algorithm updates, or bug fixes were implemented after the trial commenced."

7a) How sample size was determined

NPT: When applicable, details of whether and how the clustering by care provides or centers was addressed

7a-i) Describe whether and how expected attrition was taken into account when calculating the sample size

Describe whether and how expected attrition was taken into account when calculating the sample size.

	1	2	3	4	5	
subitem not at all important	○	○	○	○	○	essential

Does your paper address subitem 7a-i?

Copy and paste relevant sections from manuscript title (include quotes in quotation marks "like this" to indicate direct quotes from your manuscript), or elaborate on this item by providing additional information not in the ms, or briefly explain why the item is not applicable/relevant for your study

您的回答

7b) When applicable, explanation of any interim analyses and stopping guidelines

Does your paper address CONSORT subitem 7b? *

Copy and paste relevant sections from the manuscript (include quotes in quotation marks "like this" to indicate direct quotes from your manuscript), or elaborate on this item by providing additional information not in the ms, or briefly explain why the item is not applicable/relevant for your study

No
This study was designed as a fixed-sample randomized controlled trial without planned interim analyses for efficacy or futility. The sample size was determined a priori to achieve sufficient statistical power for the primary outcome (decisional conflict), and data analysis was conducted only after data collection was completed for all participants. Therefore, no formal stopping guidelines were established or implemented during the trial

8a) Method used to generate the random allocation sequence

NPT: When applicable, how care providers were allocated to each trial group

Does your paper address CONSORT subitem 8a? *

Copy and paste relevant sections from the manuscript (include quotes in quotation marks "like this" to indicate direct quotes from your manuscript), or elaborate on this item by providing additional information not in the ms, or briefly explain why the item is not applicable/relevant for your study

Yes
Participants were randomly assigned to either the Machine Learning Suggestion Group (MLSG) or the Control Group (CG) with a 1:1 allocation ratio using a computer-generated randomization sequence. The randomization process was performed using block randomization with a block size of 4 to ensure balanced enrollment between groups throughout the recruitment period."

8b) Type of randomisation; details of any restriction (such as blocking and block size)

Does your paper address CONSORT subitem 8b? *

Copy and paste relevant sections from the manuscript (include quotes in quotation marks "like this" to indicate direct quotes from your manuscript), or elaborate on this item by providing additional information not in the ms, or briefly explain why the item is not applicable/relevant for your study

"Participants were randomly assigned to either the Machine Learning Suggestion Group (MLSG) or the Control Group (CG) with a 1:1 allocation ratio using a simple randomization procedure. No blocking or stratification was applied."

9) Mechanism used to implement the random allocation sequence (such as sequentially numbered containers), describing any steps taken to conceal the sequence until interventions were assigned

Does your paper address CONSORT subitem 9? *

Copy and paste relevant sections from the manuscript (include quotes in quotation marks "like this" to indicate direct quotes from your manuscript), or elaborate on this item by providing additional information not in the ms, or briefly explain why the item is not applicable/relevant for your study

Subsequently, we conducted a parallel-group randomized controlled trial to evaluate the impact of ML-based decision aid recommendations. An additional 380 participants were recruited and randomly assigned with a 1:1 allocation ratio to either the Machine Learning Suggestion Group (MLSG, n=190) or the Control Group (CG, n=190). The randomization sequence was computer-generated and integrated directly into the web-based study platform to ensure allocation concealment. Group assignment was revealed to the research staff only after each participant's baseline data had been fully entered and locked, thereby preventing any foreknowledge of the allocation sequence.

10) Who generated the random allocation sequence, who enrolled participants, and who assigned participants to interventions

Does your paper address CONSORT subitem 10? *

Copy and paste relevant sections from the manuscript (include quotes in quotation marks "like this" to indicate direct quotes from your manuscript), or elaborate on this item by providing additional information not in the ms, or briefly explain why the item is not applicable/relevant for your study

"Subsequently, we conducted a parallel-group randomized controlled trial with a 1:1 allocation ratio... An additional 380 participants were recruited and randomly assigned to either the Machine Learning Suggestion Group (MLSG, n=190) or the Control Group (CG, n=190)..." (Section 2.1.3)
The random allocation sequence was generated by an independent statistician using a computer-generated random number list (block randomization with block sizes of 4 and 6) to ensure a 1:1 allocation ratio. Participants were enrolled by trained research assistants who verified eligibility criteria. Assignment to the intervention (MLSG) or control group (CG) was performed automatically by the web-based study interface upon completion of baseline assessments (IC-1 to IC-6), ensuring allocation concealment from both participants and enrolling staff until the point of assignment.

11a) If done, who was blinded after assignment to interventions (for example, participants, care providers, those assessing outcomes) and how

NPT: Whether or not administering co-interventions were blinded to group assignment

11a-i) Specify who was blinded, and who wasn’t

Specify who was blinded, and who wasn’t. Usually, in web-based trials it is not possible to blind the participants [1, 3] (this should be clearly acknowledged), but it may be possible to blind outcome assessors, those doing data analysis or those administering co-interventions (if any).

	1	2	3	4	5	
subitem not at all important	○	○	○	◉	○	essential

清除選取的項目

Does your paper address subitem 11a-i? *

Copy and paste relevant sections from the manuscript (include quotes in quotation marks "like this" to indicate direct quotes from your manuscript), or elaborate on this item by providing additional information not in the ms, or briefly explain why the item is not applicable/relevant for your study

Yes
"Participants and administering research staff were not blinded to group allocation due to the nature of the intervention (interactive web-based decision aid vs. standard care). However, the data analyst was blinded to group assignment during the statistical analysis."

11a-ii) Discuss e.g., whether participants knew which intervention was the “intervention of interest” and which one was the “comparator”

Informed consent procedures (4a-ii) can create biases and certain expectations - discuss e.g., whether participants knew which intervention was the “intervention of interest” and which one was the “comparator”.

	1	2	3	4	5	
subitem not at all important	○	○	○	○	○	essential

Does your paper address subitem 11a-ii?

Copy and paste relevant sections from the manuscript (include quotes in quotation marks "like this" to indicate direct quotes from your manuscript), or elaborate on this item by providing additional information not in the ms, or briefly explain why the item is not applicable/relevant for your study

您的回答

11b) If relevant, description of the similarity of interventions

(this item is usually not relevant for ehealth trials as it refers to similarity of a placebo or sham intervention to a active medication/intervention)

Does your paper address CONSORT subitem 11b? *

Copy and paste relevant sections from the manuscript (include quotes in quotation marks "like this" to indicate direct quotes from your manuscript), or elaborate on this item by providing additional information not in the ms, or briefly explain why the item is not applicable/relevant for your study

No / Not applicable
"This item is not applicable as this was an open-label randomized controlled trial evaluating a web-based decision aid. The intervention (Machine Learning Suggestion Group) and control (standard Shared Decision Making) groups received clearly distinct protocols, and blinding of participants to the intervention allocation was not feasible due to the nature of the eHealth tool."

12a) Statistical methods used to compare groups for primary and secondary outcomes

NPT: When applicable, details of whether and how the clustering by care providers or centers was addressed

Does your paper address CONSORT subitem 12a? *

Copy and paste relevant sections from the manuscript (include quotes in quotation marks "like this" to indicate direct quotes from your manuscript), or elaborate on this item by providing additional information not in the ms, or briefly explain why the item is not applicable/relevant for your study

yes
Continuous variables were compared using Student’s t-tests, and categorical variables using Chi-square tests. To address multiplicity, we prioritized the Total DCS score as the single primary endpoint; therefore, no adjustment was applied for this hypothesis. All other analyses, including subscales of the DCS, STAI, and Decision Satisfaction, were considered secondary and exploratory. For item-level comparisons within these scales, we controlled the False Discovery Rate (FDR) using the Benjamini–Hochberg procedure to minimize type I errors. Adjusted P-values are reported for these multiple comparisons. Effect sizes were reported as Cohen’s d for continuous outcomes. Mean differences (MD) with 95% confidence intervals (CIs) were calculated. Statistical significance was defined as a two-tailed P < .05. Analyses were performed using SPSS version 21.0 and R version 3.4.2.

12a-i) Imputation techniques to deal with attrition / missing values

Imputation techniques to deal with attrition / missing values: Not all participants will use the intervention/comparator as intended and attrition is typically high in ehealth trials. Specify how participants who did not use the application or dropped out from the trial were treated in the statistical analysis (a complete case analysis is strongly discouraged, and simple imputation techniques such as LOCF may also be problematic [4]).

	1	2	3	4	5	
subitem not at all important	○	○	○	◉	○	essential

清除選取的項目

Does your paper address subitem 12a-i? *

Copy and paste relevant sections from the manuscript (include quotes in quotation marks "like this" to indicate direct quotes from your manuscript), or elaborate on this item by providing additional information not in the ms, or briefly explain why the item is not applicable/relevant for your study

The current study utilized a "closed, face-to-face recruitment strategy" where all intervention components were administered on-site under the supervision of research staff. This supervised administration, combined with the "forced-entry design of the digital interface," ensured that participants could not proceed to the next step without completing required items. Consequently, data completeness was exceptionally high.
"Missing data were handled using complete case analysis, as the percentage of missing values was negligible (<1%) due to the forced-entry design of the digital interface and the supervised, face-to-face nature of the intervention. Given the extremely low rate of attrition and missingness in this controlled setting, complex imputation methods were deemed unnecessary."

The checklist item warns against complete case analysis primarily for trials with "high attrition" typical of remote eHealth interventions. In our study, the on-site, facilitated design virtually eliminated attrition during the single-session intervention, making complete case analysis a valid and unbiased approach.

12b) Methods for additional analyses, such as subgroup analyses and adjusted analyses

Does your paper address CONSORT subitem 12b? *

Copy and paste relevant sections from the manuscript (include quotes in quotation marks "like this" to indicate direct quotes from your manuscript), or elaborate on this item by providing additional information not in the ms, or briefly explain why the item is not applicable/relevant for your study

To address multiplicity, we prioritized the Total DCS score as the single primary endpoint; therefore, no adjustment was applied for this hypothesis. All other analyses, including subscales of the DCS, STAI, and Decision Satisfaction, were considered secondary and exploratory. For item-level comparisons within these scales, we controlled the False Discovery Rate (FDR) using the Benjamini–Hochberg procedure to minimize type I errors. Adjusted P-values are reported for these multiple comparisons."

"Effect sizes were reported as Cohen's d for continuous outcomes. Mean differences (MD) with 95% confidence intervals (CIs) were calculated.

X26) REB/IRB Approval and Ethical Considerations [recommended as subheading under "Methods"] (not a CONSORT item)

X26-i) Comment on ethics committee approval

	1	2	3	4	5	
subitem not at all important	○	○	○	○	○	essential

Does your paper address subitem X26-i?

Copy and paste relevant sections from the manuscript (include quotes in quotation marks "like this" to indicate direct quotes from your manuscript), or elaborate on this item by providing additional information not in the ms, or briefly explain why the item is not applicable/relevant for your study

您的回答

x26-ii) Outline informed consent procedures

Outline informed consent procedures e.g., if consent was obtained offline or online (how? Checkbox, etc.?), and what information was provided (see 4a-ii). See [6] for some items to be included in informed consent documents.

	1	2	3	4	5	
subitem not at all important	○	○	○	○	○	essential

Does your paper address subitem X26-ii?

Copy and paste relevant sections from the manuscript (include quotes in quotation marks "like this" to indicate direct quotes from your manuscript), or elaborate on this item by providing additional information not in the ms, or briefly explain why the item is not applicable/relevant for your study

您的回答

X26-iii) Safety and security procedures

Safety and security procedures, incl. privacy considerations, and any steps taken to reduce the likelihood or detection of harm (e.g., education and training, availability of a hotline)

	1	2	3	4	5	
subitem not at all important	○	○	○	○	○	essential

Does your paper address subitem X26-iii?

Copy and paste relevant sections from the manuscript (include quotes in quotation marks "like this" to indicate direct quotes from your manuscript), or elaborate on this item by providing additional information not in the ms, or briefly explain why the item is not applicable/relevant for your study

您的回答

RESULTS

13a) For each group, the numbers of participants who were randomly assigned, received intended treatment, and were analysed for the primary outcome

NPT: The number of care providers or centers performing the intervention in each group and the number of patients treated by each care provider in each center

Does your paper address CONSORT subitem 13a? *

Copy and paste relevant sections from the manuscript (include quotes in quotation marks "like this" to indicate direct quotes from your manuscript), or elaborate on this item by providing additional information not in the ms, or briefly explain why the item is not applicable/relevant for your study

"Yes, participant flow is reported in the Results section and depicted in Figure 1. A total of 380 participants were randomized (1:1 ratio). After excluding low-quality responses, 367 were analyzed (MLSG n=185; CG n=182). The intervention was delivered by a consistent team of trained research staff at a single center.""An additional 380 participants were recruited and randomly assigned... After excluding participants with low-quality responses, the final analysis included 185 participants in the MLSG (5 excluded) and 182 in the CG (8 excluded)."
"Study personnel remained consistent throughout the enrollment period... All participating medical staff completed a standardized 10-minute training session."

13b) For each group, losses and exclusions after randomisation, together with reasons

Does your paper address CONSORT subitem 13b? (NOTE: Preferably, this is shown in a CONSORT flow diagram) *

Copy and paste relevant sections from the manuscript (include quotes in quotation marks "like this" to indicate direct quotes from your manuscript), or elaborate on this item by providing additional information not in the ms, or briefly explain why the item is not applicable/relevant for your study

Yes, the manuscript addresses CONSORT subitem 13b. We detail the specific reasons for exclusion after randomization in the Methods section and visually depict these losses in the CONSORT flow diagram (Figure 1).
Relevant sections from the manuscript:
"After excluding 13 individuals due to low-quality responses—defined as ten consecutive identical scores or rhythmic scoring patterns—the remaining 507 participants formed the Model Establishment Group (MEG)... Subsequently, we conducted a parallel-group randomized controlled trial... After excluding participants with low-quality responses, the final analysis included 185 participants in the MLSG (5 excluded) and 182 in the CG (8 excluded)."
Figure 1 (Flowchart) clearly shows:
Allocated to MLSG (n=190) -> Excluded from analysis (n=5, due to low-quality data) -> Analyzed (n=185).
Allocated to CG (n=190) -> Excluded from analysis (n=8, due to low-quality data) -> Analyzed (n=182).

13b-i) Attrition diagram

Strongly recommended: An attrition diagram (e.g., proportion of participants still logging in or using the intervention/comparator in each group plotted over time, similar to a survival curve) or other figures or tables demonstrating usage/dose/engagement.

	1	2	3	4	5	
subitem not at all important	○	○	○	○	○	essential

Does your paper address subitem 13b-i?

Copy and paste relevant sections from the manuscript or cite the figure number if applicable (include quotes in quotation marks "like this" to indicate direct quotes from your manuscript), or elaborate on this item by providing additional information not in the ms, or briefly explain why the item is not applicable/relevant for your study

您的回答

14a) Dates defining the periods of recruitment and follow-up

Does your paper address CONSORT subitem 14a? *

Copy and paste relevant sections from the manuscript (include quotes in quotation marks "like this" to indicate direct quotes from your manuscript), or elaborate on this item by providing additional information not in the ms, or briefly explain why the item is not applicable/relevant for your study

Yes, the paper addresses CONSORT subitem 14a. The relevant section from the manuscript detailing the recruitment period is:"We recruited men eligible if they: (1) were aged ≥50 years, (2) presented for routine care between October 2017 and April 2018, and (3) were able to provide informed consent and communicate in Mandarin Chinese."

14a-i) Indicate if critical “secular events” fell into the study period

Indicate if critical “secular events” fell into the study period, e.g., significant changes in Internet resources available or “changes in computer hardware or Internet delivery resources”

	1	2	3	4	5	
subitem not at all important	○	○	○	○	○	essential

Does your paper address subitem 14a-i?

Copy and paste relevant sections from the manuscript (include quotes in quotation marks "like this" to indicate direct quotes from your manuscript), or elaborate on this item by providing additional information not in the ms, or briefly explain why the item is not applicable/relevant for your study

您的回答

14b) Why the trial ended or was stopped (early)

Does your paper address CONSORT subitem 14b? *

Copy and paste relevant sections from the manuscript (include quotes in quotation marks "like this" to indicate direct quotes from your manuscript), or elaborate on this item by providing additional information not in the ms, or briefly explain why the item is not applicable/relevant for your study

Yes
"A total of 520 participants were initially recruited for model development... Subsequently, we conducted a parallel-group randomized controlled trial... An additional 380 participants were recruited... After excluding participants with low-quality responses, the final analysis included 185 participants in the MLSG (5 excluded) and 182 in the CG (8 excluded)."
The trial ended upon reaching the target sample size as per the study protocol. No early stopping rules were invoked.

15) A table showing baseline demographic and clinical characteristics for each group

NPT: When applicable, a description of care providers (case volume, qualification, expertise, etc.) and centers (volume) in each group

Does your paper address CONSORT subitem 15? *

Copy and paste relevant sections from the manuscript (include quotes in quotation marks "like this" to indicate direct quotes from your manuscript), or elaborate on this item by providing additional information not in the ms, or briefly explain why the item is not applicable/relevant for your study

Yes
"A total of 367 participants were included in the analysis, consisting of 185 in the MLSG group and 182 in the CG group (Table 2). Detailed results are provided in Multimedia Appendix 12."
Table 2 in the manuscript presents baseline demographic and clinical characteristics for each group, including age, education, marital status, family history of prostate cancer, prior PSA knowledge (KnowPSA), and IPSS scores.
regarding care providers:
"Prior to study commencement, all participating medical staff completed a standardized 10-minute training session to ensure proficiency in navigating the DA... Study personnel remained consistent throughout the enrollment period."
The study was conducted at a single center (Taiwan St. Joseph Hospital), and the intervention was delivered by trained research staff rather than varying clinical providers, ensuring consistency across groups.

15-i) Report demographics associated with digital divide issues

In ehealth trials it is particularly important to report demographics associated with digital divide issues, such as age, education, gender, social-economic status, computer/Internet/ehealth literacy of the participants, if known.

	1	2	3	4	5	
subitem not at all important	○	○	○	○	○	essential

Does your paper address subitem 15-i? *

Copy and paste relevant sections from the manuscript (include quotes in quotation marks "like this" to indicate direct quotes from your manuscript), or elaborate on this item by providing additional information not in the ms, or briefly explain why the item is not applicable/relevant for your study

"Analyses were performed using SPSS version 21.0 and R version 3.4.2 (R package provided in Multimedia Appendix 10)."

"To address multiplicity, we prioritized the Total DCS score as the single primary endpoint; therefore, no adjustment was applied for this hypothesis. All other analyses, including subscales of the DCS, STAI, and Decision Satisfaction, were considered secondary and exploratory. For item-level comparisons within these scales, we controlled the False Discovery Rate (FDR) using the Benjamini–Hochberg procedure to minimize type I errors. Adjusted P-values are reported for these multiple comparisons."

16) For each group, number of participants (denominator) included in each analysis and whether the analysis was by original assigned groups

16-i) Report multiple “denominators” and provide definitions

Report multiple “denominators” and provide definitions: Report N’s (and effect sizes) “across a range of study participation [and use] thresholds” [1], e.g., N exposed, N consented, N used more than x times, N used more than y weeks, N participants “used” the intervention/comparator at specific pre-defined time points of interest (in absolute and relative numbers per group). Always clearly define “use” of the intervention.

	1	2	3	4	5	
subitem not at all important	○	○	○	○	○	essential

Does your paper address subitem 16-i? *

Copy and paste relevant sections from the manuscript (include quotes in quotation marks "like this" to indicate direct quotes from your manuscript), or elaborate on this item by providing additional information not in the ms, or briefly explain why the item is not applicable/relevant for your study

"We conducted a modified intent-to-treat (mITT) analysis including all randomized participants (N=367), excluding only 13 (3.4%) due to predetermined data quality criteria (invalid response patterns) applied blindly pre-analysis.""After excluding 13 individuals for low-quality responses, final analysis included 185 in MLSG and 182 in CG. Missing data was negligible (<1%) due to forced-entry design."

16-ii) Primary analysis should be intent-to-treat

Primary analysis should be intent-to-treat, secondary analyses could include comparing only “users”, with the appropriate caveats that this is no longer a randomized sample (see 18-i).

	1	2	3	4	5	
subitem not at all important	○	○	○	○	○	essential

Does your paper address subitem 16-ii?

Copy and paste relevant sections from the manuscript (include quotes in quotation marks "like this" to indicate direct quotes from your manuscript), or elaborate on this item by providing additional information not in the ms, or briefly explain why the item is not applicable/relevant for your study

您的回答

17a) For each primary and secondary outcome, results for each group, and the estimated effect size and its precision (such as 95% confidence interval)

Does your paper address CONSORT subitem 17a? *

Copy and paste relevant sections from the manuscript (include quotes in quotation marks "like this" to indicate direct quotes from your manuscript), or elaborate on this item by providing additional information not in the ms, or briefly explain why the item is not applicable/relevant for your study

Yes, our paper fully addresses this item. We report effect sizes (mean differences and Cohen's d) and 95% confidence intervals for all primary and secondary outcomes in the text and tables.

17a-i) Presentation of process outcomes such as metrics of use and intensity of use

In addition to primary/secondary (clinical) outcomes, the presentation of process outcomes such as metrics of use and intensity of use (dose, exposure) and their operational definitions is critical. This does not only refer to metrics of attrition (13-b) (often a binary variable), but also to more continuous exposure metrics such as “average session length”. These must be accompanied by a technical description how a metric like a “session” is defined (e.g., timeout after idle time) [1] (report under item 6a).

	1	2	3	4	5	
subitem not at all important	○	○	○	○	○	essential

Does your paper address subitem 17a-i?

Copy and paste relevant sections from the manuscript (include quotes in quotation marks "like this" to indicate direct quotes from your manuscript), or elaborate on this item by providing additional information not in the ms, or briefly explain why the item is not applicable/relevant for your study

您的回答

17b) For binary outcomes, presentation of both absolute and relative effect sizes is recommended

Does your paper address CONSORT subitem 17b? *

Copy and paste relevant sections from the manuscript (include quotes in quotation marks "like this" to indicate direct quotes from your manuscript), or elaborate on this item by providing additional information not in the ms, or briefly explain why the item is not applicable/relevant for your study

"For binary outcomes, presentation of both absolute and relative effect sizes is recommended" - Screening acceptance: MLSG "Accept" recommendation (34/67, 50.7%) vs CG (44/182, 24.2%; P<.001)

18) Results of any other analyses performed, including subgroup analyses and adjusted analyses, distinguishing pre-specified from exploratory

Does your paper address CONSORT subitem 18? *

Copy and paste relevant sections from the manuscript (include quotes in quotation marks "like this" to indicate direct quotes from your manuscript), or elaborate on this item by providing additional information not in the ms, or briefly explain why the item is not applicable/relevant for your study

"To address multiplicity, we prioritized the Total DCS score as the single primary endpoint; therefore, no adjustment was applied for this hypothesis. All other analyses, including subscales of the DCS, STAI, and Decision Satisfaction, were considered secondary and exploratory. For item-level comparisons within these scales, we controlled the False Discovery Rate (FDR) using the Benjamini–Hochberg procedure"

18-i) Subgroup analysis of comparing only users

A subgroup analysis of comparing only users is not uncommon in ehealth trials, but if done, it must be stressed that this is a self-selected sample and no longer an unbiased sample from a randomized trial (see 16-iii).

	1	2	3	4	5	
subitem not at all important	○	○	○	○	○	essential

Does your paper address subitem 18-i?

Copy and paste relevant sections from the manuscript (include quotes in quotation marks "like this" to indicate direct quotes from your manuscript), or elaborate on this item by providing additional information not in the ms, or briefly explain why the item is not applicable/relevant for your study

您的回答

19) All important harms or unintended effects in each group

(for specific guidance see CONSORT for harms)

Does your paper address CONSORT subitem 19? *

Copy and paste relevant sections from the manuscript (include quotes in quotation marks "like this" to indicate direct quotes from your manuscript), or elaborate on this item by providing additional information not in the ms, or briefly explain why the item is not applicable/relevant for your study

Relevant sections from manuscript:
"No adverse events or unintended effects were observed in either group. The intervention consisted of brief psychological assessments (STAI, DCS, Sa) and values clarification exercises (~20-25 minutes), with no physical procedures or medications administered."
This low-risk behavioral intervention had no prespecified harms monitoring as no serious adverse events were anticipated. All outcomes (anxiety, decisional conflict, satisfaction) improved in the intervention group.

19-i) Include privacy breaches, technical problems

Include privacy breaches, technical problems. This does not only include physical "harm" to participants, but also incidents such as perceived or real privacy breaches [1], technical problems, and other unexpected/unintended incidents. "Unintended effects" also includes unintended positive effects [2].

	1	2	3	4	5	
subitem not at all important	○	○	○	○	○	essential

Does your paper address subitem 19-i?

Copy and paste relevant sections from the manuscript (include quotes in quotation marks "like this" to indicate direct quotes from your manuscript), or elaborate on this item by providing additional information not in the ms, or briefly explain why the item is not applicable/relevant for your study

您的回答

19-ii) Include qualitative feedback from participants or observations from staff/researchers

Include qualitative feedback from participants or observations from staff/researchers, if available, on strengths and shortcomings of the application, especially if they point to unintended/unexpected effects or uses. This includes (if available) reasons for why people did or did not use the application as intended by the developers.

	1	2	3	4	5	
subitem not at all important	○	○	○	○	○	essential

Does your paper address subitem 19-ii?

Copy and paste relevant sections from the manuscript (include quotes in quotation marks "like this" to indicate direct quotes from your manuscript), or elaborate on this item by providing additional information not in the ms, or briefly explain why the item is not applicable/relevant for your study

您的回答

DISCUSSION

22) Interpretation consistent with results, balancing benefits and harms, and considering other relevant evidence

NPT: In addition, take into account the choice of the comparator, lack of or partial blinding, and unequal expertise of care providers or centers in each group

22-i) Restate study questions and summarize the answers suggested by the data, starting with primary outcomes and process outcomes (use)

Restate study questions and summarize the answers suggested by the data, starting with primary outcomes and process outcomes (use).

	1	2	3	4	5	
subitem not at all important	○	○	○	○	○	essential

Does your paper address subitem 22-i? *

Copy and paste relevant sections from the manuscript (include quotes in quotation marks "like this" to indicate direct quotes from your manuscript), or elaborate on this item by providing additional information not in the ms, or briefly explain why the item is not applicable/relevant for your study

Objective (Introduction):
"This study was to evaluate whether this ML-integrated strategy reduces decisional conflict (primary outcome) among middle-aged and older men compared to standard care."

Principal Findings (Discussion):
"Regarding the primary outcome, the Total DCS score was significantly lower in the MLSG compared with the CG (mean difference [MD] −3.77, 95% CI −5.55 to −1.99; Cohen d = −0.44; P < .001), indicating superior overall decision quality."

Process outcomes (Results 3.4):
"Decision satisfaction showed large improvement (Total Sa score: MD −7.38, 95% CI −8.54 to −6.18; d = −1.29; P < .001). ML recommendations strongly influenced behavior: 'Accept' recommendation group (34/67, 50.7%) vs CG (44/182, 24.2%; P<.001); 'Not Now' recommendation (21/118, 17.8%)."

22-ii) Highlight unanswered new questions, suggest future research

Highlight unanswered new questions, suggest future research.

	1	2	3	4	5	
subitem not at all important	○	○	○	○	○	essential

Does your paper address subitem 22-ii?

Copy and paste relevant sections from the manuscript (include quotes in quotation marks "like this" to indicate direct quotes from your manuscript), or elaborate on this item by providing additional information not in the ms, or briefly explain why the item is not applicable/relevant for your study

您的回答

20) Trial limitations, addressing sources of potential bias, imprecision, and, if relevant, multiplicity of analyses

20-i) Typical limitations in ehealth trials

Typical limitations in ehealth trials: Participants in ehealth trials are rarely blinded. Ehealth trials often look at a multiplicity of outcomes, increasing risk for a Type I error. Discuss biases due to non-use of the intervention/usability issues, biases through informed consent procedures, unexpected events.

	1	2	3	4	5	
subitem not at all important	○	○	○	○	○	essential

Does your paper address subitem 20-i? *

Copy and paste relevant sections from the manuscript (include quotes in quotation marks "like this" to indicate direct quotes from your manuscript), or elaborate on this item by providing additional information not in the ms, or briefly explain why the item is not applicable/relevant for your study

Several limitations should be noted. First, while we provided uniform technical assistance, we did not formally assess baseline eHealth literacy... Second, although our model demonstrated high discrimination (AUC > 0.90), calibration metrics were not assessed... Third, unmeasured confounding remains a possibility..."

21) Generalisability (external validity, applicability) of the trial findings

NPT: External validity of the trial findings according to the intervention, comparators, patients, and care providers or centers involved in the trial

21-i) Generalizability to other populations

Generalizability to other populations: In particular, discuss generalizability to a general Internet population, outside of a RCT setting, and general patient population, including applicability of the study results for other organizations

	1	2	3	4	5	
subitem not at all important	○	○	○	○	○	essential

Does your paper address subitem 21-i?

Copy and paste relevant sections from the manuscript (include quotes in quotation marks "like this" to indicate direct quotes from your manuscript), or elaborate on this item by providing additional information not in the ms, or briefly explain why the item is not applicable/relevant for your study

您的回答

21-ii) Discuss if there were elements in the RCT that would be different in a routine application setting

Discuss if there were elements in the RCT that would be different in a routine application setting (e.g., prompts/reminders, more human involvement, training sessions or other co-interventions) and what impact the omission of these elements could have on use, adoption, or outcomes if the intervention is applied outside of a RCT setting.

	1	2	3	4	5	
subitem not at all important	○	○	○	○	○	essential

Does your paper address subitem 21-ii?

Copy and paste relevant sections from the manuscript (include quotes in quotation marks "like this" to indicate direct quotes from your manuscript), or elaborate on this item by providing additional information not in the ms, or briefly explain why the item is not applicable/relevant for your study

您的回答

OTHER INFORMATION

23) Registration number and name of trial registry

Does your paper address CONSORT subitem 23? *

Copy and paste relevant sections from the manuscript (include quotes in quotation marks "like this" to indicate direct quotes from your manuscript), or elaborate on this item by providing additional information not in the ms, or briefly explain why the item is not applicable/relevant for your study

"The trial was registered in the Chinese Clinical Trial Registry (ChiCTR 2000034126)."
"Trial Registration: Chinese Clinical Trial Registry ChiCTR2000034126;
https://www.chictr.org.cn/showprojEN.html?proj=55682"Registration number and name: ChiCTR2000034126 (Chinese Clinical Trial Registry)

24) Where the full trial protocol can be accessed, if available

Does your paper address CONSORT subitem 24? *

Cite a Multimedia Appendix, other reference, or copy and paste relevant sections from the manuscript (include quotes in quotation marks "like this" to indicate direct quotes from your manuscript), or elaborate on this item by providing additional information not in the ms, or briefly explain why the item is not applicable/relevant for your study

"The full trial protocol is not publicly available. The study was approved by the Institutional Review Board of Fu Jen Catholic University (C105142) and registered at Chinese Clinical Trial Registry (ChiCTR2000034126)."

25) Sources of funding and other support (such as supply of drugs), role of funders

Does your paper address CONSORT subitem 25? *

Copy and paste relevant sections from the manuscript (include quotes in quotation marks "like this" to indicate direct quotes from your manuscript), or elaborate on this item by providing additional information not in the ms, or briefly explain why the item is not applicable/relevant for your study

"This study was supported by institutional research funds from Fu Jen Catholic University (IRB C105142). The funders had no role in study design, data collection, analysis, interpretation, or manuscript preparation."

X27) Conflicts of Interest (not a CONSORT item)

X27-i) State the relation of the study team towards the system being evaluated

In addition to the usual declaration of interests (financial or otherwise), also state the relation of the study team towards the system being evaluated, i.e., state if the authors/evaluators are distinct from or identical with the developers/sponsors of the intervention.

	1	2	3	4	5	
subitem not at all important	○	○	○	○	○	essential

Does your paper address subitem X27-i?

Copy and paste relevant sections from the manuscript (include quotes in quotation marks "like this" to indicate direct quotes from your manuscript), or elaborate on this item by providing additional information not in the ms, or briefly explain why the item is not applicable/relevant for your study

您的回答

About the CONSORT EHEALTH checklist

As a result of using this checklist, did you make changes in your manuscript? *

- (●) yes, major changes
- ( ) yes, minor changes
- ( ) no

What were the most important changes you made as a result of using this checklist?

您的回答

How much time did you spend on going through the checklist INCLUDING making changes in your manuscript *

2DAYS

As a result of using this checklist, do you think your manuscript has improved? *

- (●) yes
- ( ) no
- ( ) 其他：

Would you like to become involved in the CONSORT EHEALTH group?

This would involve for example becoming involved in participating in a workshop and writing an "Explanation and Elaboration" document

- yes
- no
- 其他：

Any other comments or questions on CONSORT EHEALTH

您的回答

STOP - Save this form as PDF before you click submit

To generate a record that you filled in this form, we recommend to generate a PDF of this page (on a Mac, simply select "print" and then select "print as PDF") before you submit it.

When you submit your (revised) paper to JMIR, please upload the PDF as supplementary file.

Don't worry if some text in the textboxes is cut off, as we still have the complete information in our database. Thank you!

Final step: Click submit !

Click submit so we have your answers in our database!

提交 清除表單

請勿利用 Google 表單送出密碼。

Google 並未認可或建立這項內容。 - 聯絡表單擁有者 - 服務條款 - 隱私權政策

這份表單很可疑嗎？ 報告

Google 表單
